# Effect of infundibulopelvic angle on outcomes of ureteroscopy: a systematic review and meta-analysis

**DOI:** 10.1007/s00345-024-05104-z

**Published:** 2024-07-16

**Authors:** James Leighton, Arran Dingwall, Sophie Whitehead, Angus Luk, Vineet Gauhar, Bhaskar Somani, Robert Geraghty

**Affiliations:** 1 Department of Urology, Freeman Hospital, Freeman Road, Newcastle upon Tyne, UK; 2 Department of Urology, Ng Teng Fong General Hospital, Singapore, Singapore; 3 Department of Urology, University Hospital Southampton, Tremona Road, Southampton, UK; 4 Biosciences Institute, Newcastle University, Newcastle upon Tyne, UK

**Keywords:** Ureteroscopy, Lower Pole, Infundibulopelvic angle, RIRS

## Abstract

**Purpose:**

The infundibulo-pelvic angle (IPA) is reportedly a predictor of successful ureteroscopy for lower pole renal stones, however there is uncertainty at which IPA success is likely. We therefore aimed to perform a meta-analysis and determine at which the angle of likely success and failure.

**Methods:**

We performed a systematic review and meta-analysis as per Cochrane guidelines in accordance to the PRISMA statement. The review was registered with PROSPERO prior to commencement (ID: CRD42022296732). We included studies reporting on outcomes of ureteroscopy for lower pole stones, with IPA. We excluded patients undergoing alternative treatments for lower pole stones, anatomical abnormalities and studies with < 10 patients. We assessed bias with the Newcastle-Ottawa scale. We performed meta-analysis in R, and summarised the findings as per GRADE.

**Results:**

Overall, there were 13 studies included, with 10 included for meta-analysis. These studies covered *n* = 1964 patients (71% stone free). Overall, the stone free patients had a significantly less acute mean IPA (52^o^ ± 9^o^), compared to the non-stone free patients (39^o^ ± 7^o^), on meta-analysis (REM MD = -13.0, 95% CI: -18.7 to -7.2, *p* < 0.001). On examination of forest plots, at IPA < 30^o^ no patients were stone free, whilst > 50^o^ all were stone free. Risk of bias was moderate, and certainty of evidence was ‘very low’.

**Conclusion:**

With a very low certainty of evidence, we demonstrate that at an IPA of < 30^o^ no patient is stone free, whilst > 50^o^ all patients (in this review) are stone free. More evidence is therefore needed.

**Supplementary Information:**

The online version contains supplementary material available at 10.1007/s00345-024-05104-z.

## Introduction

Kidney stone disease is a prevalent [1] and costly condition [2], which is increasingly being managed with ureteroscopy over other forms of intervention throughout the world [3]. Lower pole stones (LPS) are a technical challenge, and thus many aspects of the lower pole anatomy have been investigated to identify positive and negative predictors of success [4]. One such aspect is the infundibulopelvic angle (IPA), which is often defined as “the inner angle formed at the intersection of the ureteropelvic axis and the central axis of the lower pole infundibulum” [5, 6]. It has been suggested that the IPA is a predictive factor for achieving stone free status following ureteroscopy [7]. Although, the definition of ‘stone free’ varies from no fragments to fragments < 4 mm [8]. Given the complexity of accessing the lower pole, there are concerns that stone free rates are lower, with a reciprocal rise in operative time and complication rates. We therefore aimed to perform a systematic review and meta-analysis of all studies comparing stone free rates (SFR – study defined) in relation to the IPA, along with secondary analyses of operative time and complication rate.

## Methods and materials

This systematic review and meta-analysis was performed according to the Preferred Reporting Items for Systematic reviews and Meta-analyses (PRISMA) statement. See Appendix 1 for PRISMA statement.

### Literature search

A literature search was performed using Medline/PubMed, EMBASE, CINAHL and Cochrane Library, while registered randomized controlled trial protocols were screened from clinicaltrial.gov from 1970 to 01/02/2024. The reference lists of all manuscripts reviewed as full-text, were also screened for eligible studies. Two independent authors (JL, AD) screened the databases and disagreements were resolved upon consensus with a senior author (RG).

Literature search was carried out based on a search strategy consisting of the following search terms: retrograde intrarenal surgery, RIRS, retrograde Ureteroscopy, Ureteroscopy, URS, ureterorenoscopy, pelvicalyceal anatomy, calculi, stone, infundibular width, infundibular height, pelvicalyceal angle, IPA, Renal Infundibulopelvic angle, RIPA, and infundibular pelvic angle.

### Study eligibility criteria

The initial protocol was registered on the PROSPERO Database (ID: CRD42022296732). We included randomised clinical trials (RCT) and comparative non-randomised studies. We prioritised RCTs and prospective studies, with the use of retrospective studies only in the circumstance of no higher level study availability.

Our inclusion criteria were: English language articles, adults (> 18 years), either sex, studies on RIRS for LPS with information on pelvicalyceal anatomy in particular IPA. We excluded patients with urogenital abnormalities (horseshoe kidneys etc.), studies with < 10 patients, studies reporting on PCNL or ESWL, animal studies, technical studies and case reports.

Our primary outcome was stone free status dependant on infundibulopelvic angle (IPA), with secondary outcomes of operative time and complication rate (as graded by Clavien Dindo classification [9]). Our primary aim was to ascertain the IPA at which ureteroscopy is not feasible, with a secondary outcome of determining above which IPA is ureteroscopy likely to be successful (the ‘angle of likely success’).

### Data collection

Two authors (JL, AD) independently extracted data from eligible studies, using a pre-defined Excel sheet, with any conflicts resolved by the senior author (RG). Study characteristics (author, country/centre, period, retrospective/prospective design, ) and patient baseline characteristics (number of patients, age, male proportion, treatment modality, mean IPA, type of flexible URS, type of lithotripsy). We also extracted outcome data for: stone free rate, stone free definition and post operative imaging.

If mean and standard deviation were unavailable, then these were estimated from sample size, median and range/inter-quartile range if provided as per Wan et al. [10].

### Risk of bias assessment

Two authors (JL, RG) assessed the risk of bias of studies individually with the Cochrane risk of bias assessment tool for RCTs [11] and the Newcastle-Ottawa scale for observational studies [12].

### Publication bias assessment

Publication bias was assessed via visual inspection of Funnel plots and statistically compared with Cochran’s Q, and trim and fill adjustment (if the number of studies available for analysis was more than 3).

### Statistical analysis

All statistical analyses were performed using R (R Statistical software, Vienna, Austria) with the ‘‘meta’’ package [13]. Meta-analyses were performed using a random-effects model if there was concern over heterogeneity of outcome, with fixed-effects reserved for analyses with no concerns as per the Cochrane reviews guidelines [14]. Heterogeneity was assessed using I^2^, tau^2^, and Cochran’s Q. We present the meta-analysis result (mean differences – MD) according to the random- or fixed-effect model as above. In the context of angle, an overall meta-analysis is not informative, and visual inspection of the summary statistics was felt to be more helpful for our primary outcome. We performed trim and fill analyses to statistically assess publication bias. Adjusted values for the trim and fill analyses are presented along with the calculated number of missing studies. For statistical code see Appendix 2.

### Assessment of certainty of evidence

The certainty of presented evidence was assessed using the Grading of Recommendations Assessment, Development and Evaluation (GRADE) system for primary outcomes, as described above. Five factors were considered: risk of bias, imprecision, inconsistency, indirectness and publication bias. As instructed by the tool, we reduced the level of evidence from RCTs by one for serious risk of bias (or by two for very serious, indirectness of evidence, serious inconsistency, imprecision or high risk for publication bias). If analyses are amenable, then GRADE summaries were produced using the GRADEpro tool [15].

## Results

### Study/patient demographics

Overall, there were 13 [5, 16–27] studies meeting inclusion criteria [see supplementary Fig. 1]. These studies included *n* = 1964 patients [see Table 1], of whom *n* = 1397 (71%) were stone free. The studies were published between 1998 and 2022. They originated from a range of centres around the world. These studies were all retrospective cohort studies, as no RCTs or prospective comparative cohort studies were found. The studies used a variety of scope types and stone free definitions [Table 1], which we have sub-analysed [see below]. All the studies used a similar definition of the IPA, albeit in differing modalities of imaging [see Table 2]. Of these 13 studies, 3 exclusively examined angle cut-offs and the resultant stone free rates [17, 24, 27] whilst a further study detailed segregated outcomes by both stone free status and angle cut-off [23] [see Table 2], whilst the remainder compared the IPA of stone free patients to those who were not stone free. The 3 studies exclusively examining angle cut-offs were not included in the meta-analysis, but provide useful information and therefore were included.

**Table 1:** Study demographics

Author	Country	Study Type	Total *N* (% Men)	Mean Age ± SD (Range)	Overall mean stone size ± SD (Range)	Stone Free (%)	Stones > 20 mm treated	Stone Free Definition
Sari et al. 2017 [11]	Turkey	Retrospective cohort	132 (60%)	Success: 47 ± 14 Failure: 50 ± 12	Success: 10 (6–54) Failure: 19 (8–45)	78	Yes	0 visible on XR (opaque) US (non Opaque) 24 h after procedure
Resorlu et al. 2012 (a) [12]	Turkey	Retrospective cohort	88 (54%)	33 ± 19	16 ± 4 (8–40)	78	Yes	< 1 mm on US day 1, CT 1 month
Elbahnasy et al. 1998 [22]	USA	Retrospective cohort	13 (54%)	52 (23–77)	11 (5–15)	62	No	No fragments on XR +/- IVP at 4 weeks and 3 months
Resorlu et al. 2012 (b) [13]	Turkey	Retrospective cohort	67 (64%)	36 ± 17	17 ± 3 (13–67)	81	Yes	< 4 mm at 2 months on AXR + US +/- CT
Jessen et al. 2014 [14]	Germany	Retrospective cohort	111 (23%)	51 ± 16	Success: 7 ± 3 Failure: 12 ± 7	88	No	No fragments intraoperatively by radiologic and endoscopic control + US day 1
Geavlete et al. 2008 [19]	Romania	Retrospective cohort	47 (N/A)	N/A	9 (5–12)	70	No	< 2 mm, modality not specified
Inoue et al. 2005 [15]	Japan	Retrospective cohort	77 (N/A)	Success: 61 ± 13 Failure: 59 ± 10	Success: 27 ± 11 Failure: 29 ± 11	71	Yes	No fragments on XR and US at 3 months
Tastemur et al. 2022 [16]	Germany	Retrospective cohort	168 (63%)	46 ± 14	Success: 13 ± 5 Failure: 19 ± 8	63	Yes	1 month CT or US - no fragments or asymptomatic < 2 mm residual stones
Wang et al. 2021 [17]	China	Retrospective cohort	147 (79%)	52 ± 21 (40–61)	Success: 12 ± 8 Failure: 16 ± 14	71	Yes	1 month CT – no fragments
Richard et al. 2020 [18]	France	Retrospective cohort	669 (62%)	53 ± 17	12 ± 9	72	Yes	6 month CT no fragments
Karim et al.2019 [2]	UK	Retrospective cohort	108 (40%)	55 (18–86)	Success: 9 ± 5 Failure: 15 ± 6	94	No	< 2 mm fragments on 2–3 month CT or US
Kilicarslan et al. 2015 [20]	Turkey	Retrospective cohort	36 (56%)	Success: 46 ± 10 Failure: 47 ± 13	Success: 10 (5–35) Failure: 12 (5–15)	47	Yes	No fragments on XR (Radio-opaque stones) or CT (radiolucent stones)
Xiao et al. 2017 [21]	China	Retrospective cohort	382 (%)	52 ± 12	Success: 12 ± 8 Failure: 25 ± 11	74	Yes	Fragments < 2 mm at 1 month on XR

SD = standard deviation

**Table 2:** Equipment demographics and angle definitions

Author	Scope Used	Laser Used	IP Angle Imaging	IP Angle Measure Definition	States Explicity Definition as per Elbahnasy et al. 1998?
Sari et al. 2017 [11]	Flex X2	HoYAG; Manufacturer NOS; 200micron fibre	XR IVU	“IPA is the angle formed when the axis that passes through lower calyx and the axis that passes through ureteropelvic junction intersect”	No
Resorlu et al. 2012 (a) [12]	7.5Fr KS or 8.4Fr Olympus	HoYAG Manufacturer NOS; Fibre NOS	XR IVU, CTU or CT KUB	“The infundibulopelvic angle (IPA) was measured as an inner angle formed at the intersection of the ureteropelvic axis defined by Elbahnasy and associates and the central axis of the lower pole infundibulum”	Yes
Elbahnasy et al. 1998 [22]	9.5Fr or 7.5Fr	HoYAG; Manufacturer NOS; Fibre NOS	XR IVU	“Using an antero- posterior radiograph from the IVP the inner angle between this line and the central axis of the lower pole infundibulum was measured (infundibulopelvic angle)”	Yes
Resorlu et al. 2012 (b) [13]	7.5Fr KS or 8.5Fr Olympus	HoYAG; Manufacturer NOS; 273micron fibre	XR IVU	“The IPA of the lower calyces was measured as an inner angle formed at the intersection of the ureteropelvic axis defined by Elbahnasy et al and the central axis of the lower pole infundibulum”	Yes
Jessen et al. 2014 [14]	Flex X2	HoYAG; Auriga XL; Fibre NOS	XR IVU or RPG	“IPA was determined as the inner angle at the intersection of ureteropelvic axis and central axis of lower infundibulum”	Yes
Geavlete et al. 2008 [19]	7.5fr KS	HoYAG; Manufacturer NOS; 200micron fibre	XR IVU or RPG	“The infundibulopelvic angle was measured between the inferior calyx axis and the ureteropelvic one”	Yes
Inoue et al. 2005 [15]	Flex X2 or Olympus	HoYAG; Odyssey 30, Fibre NOS	XR IVU	“The IPA was measured as the inner angle formed at the intersection of the ureteropelvic axis”	Yes
Tastemur et al. 2022 [16]	7.5fr KS	HoYAG Manufacturer NOS; 200 micron fibre	CTU	“IPA was measured as the internal angle formed by the intersection of the ureteropelvic axis and central axis of the infundibulum of the lower pole”	No
Wang et al. 2021 [17]	Undocumented	HoYAG Manufacturer NOS; Fibre NOS	CTU	“The renal infundibulopelvic angle (RIPA) of the inferior pole stone was defined as the inner angle of the intersection of ureteropelvic axis and the axis of the lower renal calyx”	No
Richard et al. 2020 [18]	Flex X2 or Olympus URF-V	HoYAG Manufacturer NOS; 272micron fibre	CTU	As per RUSS score	No
Karim et al.2019 [2]	Flex X2	HoYAG Manufacturer NOS; Fibre NOS	CT KUB or RPG	“Measurement of the IPA was completed using the Elbahnasy method.”	Yes
Kilicarslan et al. 2015 [20]	Flex X2	HoYAG; Lumenis; 272micron fibre	XR IVU	“The IPA of lower calyx was measured as the inner angle formed at the intersection of the ureteropelvic and central axis of the lower pole infundibulum”	Yes
Xiao et al. 2017 [21]	Olympus URF-V	HoYAG Manufacturer NOS; Fibre NOS	CTU	“The renal infundibulopelvic angle (RIPA) of the inferior pole stone was defined as the inner angle of the intersection of ureteropelvic axis and the axis of the lower renal calyx”	No

KS = Karl Storz, HoYAG = Holmium Yttrium Aluminium Garnet, NOS = not otherwise specified, XR = X-Ray, IVU = Intravenous urogram, RPG = retrograde pyelogram, CTU = computed tomography urogram, IP = infundibulo-pelvic

Unfortunately, too few studies subdivided their complication rates by either angle or stone free status, and therefore meta-analysis was not feasible. Overall complication rates for each study are detailed in supplementary Table 1.

### Meta-analysis of infundibulopelvic angle by stone free status

There were 10 included studies [5, 16, 18–23, 25, 26], with *n* = 1357 patients of whom *n* = 974 (72%) were stone free. The means and standard deviations of each study are detailed in supplement 1 within the summary forest plot (Sect. 4.2). Overall, the stone free patients had a mean IPA of 52^o^ ± 9^o^, compared to the non-stone free patients, who had a mean IPA of 39^o^ ± 7^o^ (see Appendix 2 - Sect. 4.2).

On meta-analysis there was a significant difference between these angles (REM MD = -13.0, 95% CI: -18.7 to -7.2, *p* < 0.001). The results of the sensitivity analyses suggested significant heterogeneity and statistical evidence of publication bias (see Appendix 2 - Sect. 4.3).

**Fig. 1:**
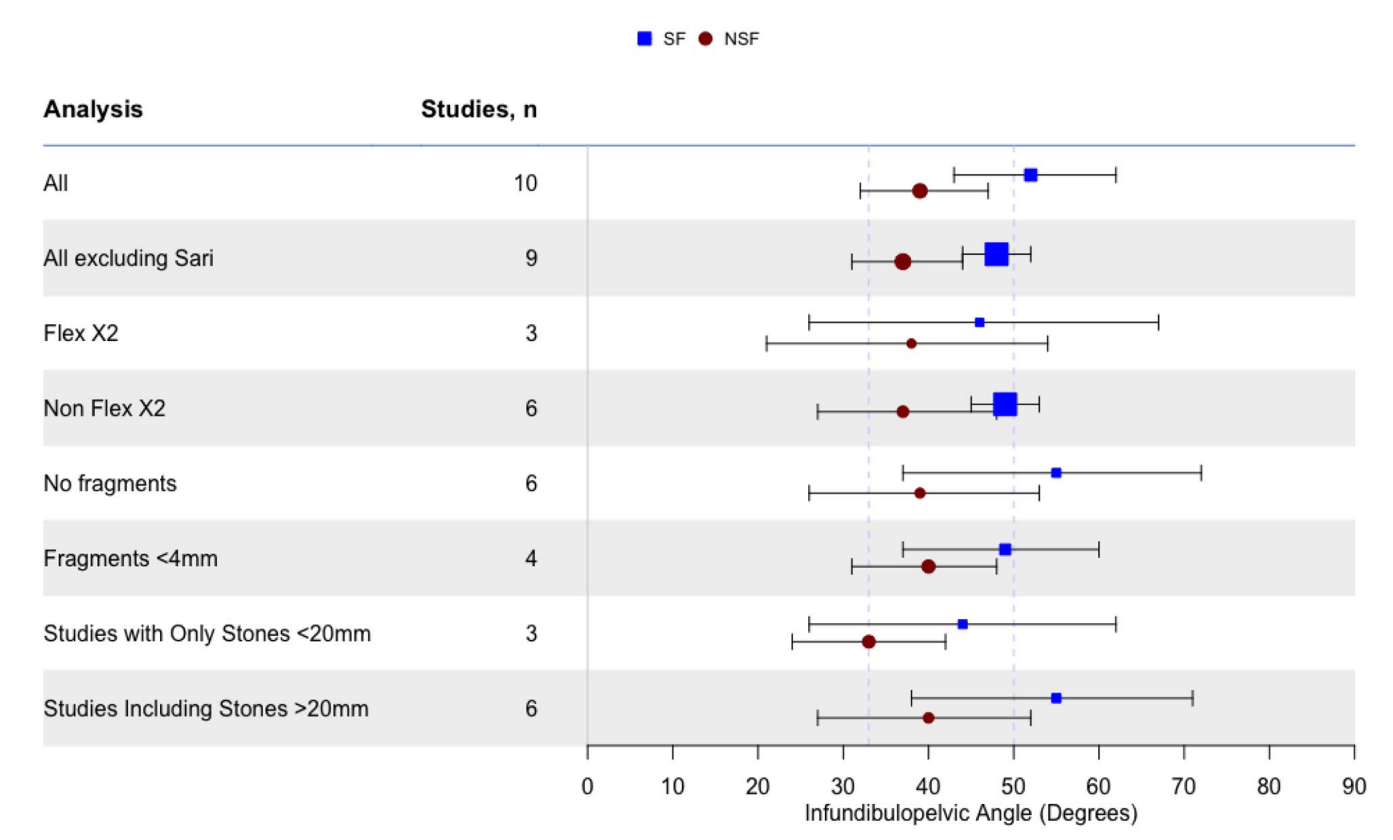
Summary forest plot of mean angles from meta-analyses as detailed in Analysis column, subdivided by stone free and not stone free

We performed further sensitivity analyses (all detailed in Appendix 2) by removing Sari et al. 2017 from the overall analysis (outlier on Baujat plot – see Sect. 5), and sub-analyses for scope type (Storz Flex X2 vs. non Flex X2 – see Sects. 6&7), stone free definition (no fragments vs. fragments < 4 mm – see Sects. 8&9), large stones inclusion (> 2 cm stones included or not – see Sects. 10&11). All of these analyses replicated the significant findings of the primary analysis, with those who were stone free having significantly shallower angles than those who were not stone free. Similar findings were found for heterogeneity and statistical evidence of publication bias. We have summarised the results for the mean angles for each of our analyses in Fig. 1.

### Stone free status by pre-defined infundibulopelvic angle cut-off

There were 4 studies [17, 23, 24, 27] that detailed stone free status by IPA cut-off [see supplementary Table 2]. Unfortunately, due to the heterogeneity of the study-defined angle cut-offs and inclusion of stones > 2 cm in some studies, it was felt that meta-analysis was not appropriate. On review of Table 4, it seems that steeper (more acute) angles have lower stone free rates.

### Operative time

There were 5 studies that detailed operative time and stone free status [5, 16, 21, 25, 26]. One further study detailed operative time [24] by angle which, within the study, demonstrated a significant difference between steep (72.6 ± 49.2 min) and shallow (48.2 ± 26.5 min) angles. They used a cut-off of 70^o^.

For the 5 studies examining stone free status and operative time, there was a small, but significant difference in operative time between stone free and non-stone free groups (48 ± 6 min vs. 59 ± 16 min, REM MD = 7.8, 95% CI: 2.0-13.7, *p* = 0.01). There was minimal evidence of heterogeneity and no statistical detection of publication bias (see Appendix 2 – Sect. 14).

### Bias analysis and GRADE assessment

Overall, there was a moderate risk of bias [see supplementary Table 3]. Studies were mainly downgraded for not adhering to stringent follow-up protocols. Due to the low numbers of studies with angle cut-off data, it was not possible to generate a GRADE summary [15] for stone free status (no risk ratio). However, GRADE summaries were generated for IPA by stone free status, and operative time [see supplementary Table 4], but are graded as very low and low certainty of evidence, respectively.

## Discussion

### Meaning of our study

In this study we examined patients with lower pole stones treated with ureteroscopy. In patients who are stone free, the infundibulopelvic angle is significantly shallower (i.e. higher angle) than those who were not stone free. This is a finding conserved across sensitivity and sub-analyses. The optimum angle seems to be above ∼ 50^o^ (all patients appear to be stone free) whilst the opposite is true of angles under ∼ 30^o^. Operative time is also significantly less in these stone free patients, implying that shallower angles lead to decreased operative times. Unfortunately, there was not enough data to analyse complication rates.

### Strengths, limitations and areas of future research

The major strength of this study is the robust methodology employed to ascertain and analyse the data. It is notable that in the sensitivity / sub-analyses the main findings were replicated. The finding of the 30^o^ cut-off has previously been replicated in both primary studies [27] and previous reviews [5]. However, there is more debate in the literature about the upper limit i.e. the ‘angle of likely success’. This review has shown the range of upper limits demonstrated in the literature (see Table 4). By closely examining the mean angles of stone free and non-stone free, it is possible to ascertain a minimum ‘angle of likely success’ ( > ∼ 50^o^).

There are several limitations to this review. As with all similar studies, this review is dependent on the data available. As demonstrated in the bias analysis and GRADE summary, the level of evidence is poor. The heterogeneity of reporting also makes this a difficult area to analyse. The main two examples are the definitions of stone free status and the infundibulopelvic angle. There is much debate in the literature about what the ‘gold standard’ stone free status ascertainment would look like. The generally accepted current gold standard is no stone fragments on non-contrast CT at 3 months [28]. However, the European Association of Urology have recently published guidelines suggesting that x-ray and/or ultrasound may be used instead [29], although the ‘no fragments’ part of the definition remains. This is a pragmatic approach, as CT may not be available or practical in all countries. In this review, there were a wide range of stone free definitions including a range of modalities, fragment sizes and follow-up intervals. There is less certainty in the literature about the gold standard definition of the IPA or lower pole angle [7]. To date there are five methods of ascertaining the IPA, all of which differ slightly [26]. The lack of standardisation may interfere with the clinical application of IPA as a predictive factor. In this review, most of the studies used Elbahnasy’s method, which diminishes the potential impact heterogeneity may have on the outcome.

In this review we have not considered other anatomical features have been suggested to impact on stone clearance rates. These include infundibulopelvic length and infundibulopelvic width [5]. This makes logical sense, in that a long, narrow and steep lower pole is unlikely to be amenable to ureteroscopy, and therefore a percutaneous approach ought to be considered. A recent Cochrane review has suggested that PCNL may improve the SFR compared to ureteroscopy, with little effect on major complications and reduced need for secondary interventions, albeit with low certainty of evidence [30]. However, the IPA was not considered and therefore there may yet be a role for ureteroscopy. If we use this study as a guide, ureteroscopy could be considered in those with > 30^o^ IPA.

Over the past two decades reusable ureteroscopic technology has transitioned from fibre-optic to digital scopes. The newer digital scopes seem to have poorer ‘end-tip’ deflection than their fibre-optic counterparts [31]. However, single-use ureteroscopes have recently entered the market, which seem to negate this issue with end-tip deflection, although one sacrifices visual characteristics [32]. None of the studies used single-use ureteroscopes, with the majority using digital reusable scopes (Olympus URV-F or Karl Storz Flex X2). These have a maximum deflection of 270^o^ and 275^o^, respectively. However, to achieve access to a < 30^o^ IPA lower pole, a very high deflection angle needs to be achieved (ideally > 330^o^). Therefore it is theoretically impossible for these scopes to access a lower pole with an IPA of < 30^o^. For IPA > 50^o^ the deflection needed is < 310^o^, which may be achieved using a J-manouvre [31]. Other anatomical features will help with this such as a wide infundibular width. This is clearly a novel area that needs to be explored with up to date studies.

Of note, the PUrE trial protocol was published in 2020 [33] and abstracts presented at both AUA and EAU (2024) [34]. These abstracts suggest that in < 10 mm stones ESWL is more cost effective despite higher stone free rates in ureteroscopy, and in 10–25 mm stones PCNL is more cost and clinically effective than ureteroscopy. We await the full results, as it is unclear if IPA data has been included in their analyses.

As the evidence base is clearly heterogenous and poor, the recommendation must be for a dedicated randomised controlled trial. This should take into account metrics of lower pole anatomy including IPA, but also IW and IL, as well as stone size. However, using this study to guide clinical practice within the context of the other studies mentioned above we suggest the following: ureteroscopy should not be offered to those with an IPA < 30^o^, with PCNL being a reasonable alternative. For angles above 30^o^ the likelihood of success rises, with those > 50^o^ having the highest stone free rates. In this scenario stone, anatomical and patient factors should be taken into account and a pragmatic discussion had with the patient to inform their decision making.

## Conclusion

In this systematic review and meta-analysis, we suggest that the upper and lower cut-off values of the infundibulo-pelvic angle for determining stone free status following ureteroscopy for a lower pole stone should be 50^o^ and 30^o^, respectively. The evidence certainty is graded as very low and superior trial designs are needed to accurately answer this pertinent question. However, we make the following interpretations: At the lower cut-off ureteroscopy is unlikely to be successful and therefore alternative approaches should be considered. Above the lower cut-off, a pragmatic discussion should be had with each patient on the likelihood of success taking into account patient, stone and anatomical features.

## Electronic supplementary material

Below is the link to the electronic supplementary material.

Supplementary Material 1

Supplementary Material 2
