# Supplementary material for: Effect of infundibulopelvic angle on outcomes of ureteroscopy: a systematic review and meta-analysis
Source: World J Urol. 2024 Jul 16;42(1):413. doi: 10.1007/s00345-024-05104-z (PMC11252207; doi:10.1007/s00345-024-05104-z)

URS for Lower Pole stones - analysis of the LP Angle

R Geraghty

2022-12-21

Contents

1 Setup **3**

2 Data Exploration **4**
2.1 Plot missing data 4

3 Summary Table **5**

4 SF cut-off **6**
4.1 Sort Data 6
4.1.1 Overall Number of patients 7
4.1.2 SF - Number of patients 8
4.1.3 NSF - Number of patients 9
4.2 Summary forestplot for SF 10
4.3 Meta-Analysis 13
4.3.1 Result 13
4.3.2 Forest plot 14
4.3.3 Trim and Fill 15
4.3.4 Baujat 16

5 Exclude Sari **18**
5.0.1 Summary forest plot 18
5.0.2 Result 21
5.0.3 Forest plot 22
5.0.4 Trim and Fill 23
5.0.5 Baujat 24

6 SF cut-off for Flex X2 **26**
6.1 Sort Data 26
6.1.1 Overall Number of patients 27
6.1.2 SF - Number of patients 28
6.1.3 NSF - Number of patients 29
6.2 Summary foresplot for SF 30
6.3 Meta-Analysis 33
6.3.1 Result 33
6.3.2 Forest plot 34
6.3.3 Trim and Fill 35
6.3.4 Baujat 36

7 SF cut-off for Non Flex X2 **37**
7.1 Sort Data 37
7.1.1 Overall Number of patients 38

7.1.2 SF - Number of patients . . . 39
7.1.3 NSF - Number of patients . . . 40
7.2 Summary forestplot for SF . . . 41
7.3 Meta-Analysis . . . 44
7.3.1 Result . . . 44
7.3.2 Forest plot . . . 45
7.3.3 Trim and Fill . . . 46
7.3.4 Baujat . . . 47

8 SF cut-off for No fragments **49**
8.1 Sort Data . . . 49
8.1.1 Overall Number of patients . . . 50
8.1.2 SF - Number of patients . . . 51
8.1.3 NSF - Number of patients . . . 52
8.2 Summary forestplot for SF . . . 53
8.3 Meta-Analysis . . . 56
8.3.1 Result . . . 56
8.3.2 Forest plot . . . 57
8.3.3 Trim and Fill . . . 58
8.3.4 Baujat . . . 59

9 SF cut-off for <4mm fragments (any accepted fragment size) **61**
9.1 Sort Data . . . 61
9.1.1 Overall Number of patients . . . 62
9.1.2 SF - Number of patients . . . 63
9.1.3 NSF - Number of patients . . . 64
9.2 Summary forestplot for SF . . . 65
9.3 Meta-Analysis . . . 68
9.3.1 Result . . . 68
9.3.2 Forest plot . . . 69
9.3.3 Trim and Fill . . . 70
9.3.4 Baujat . . . 71

10 SF for LP stones <2cm **72**
10.1 Sort Data . . . 72
10.1.1 Overall Number of patients . . . 73
10.1.2 SF - Number of patients . . . 74
10.1.3 NSF - Number of patients . . . 75
10.2 Summary forestplot for SF . . . 76
10.3 Meta-Analysis . . . 79
10.3.1 Result . . . 79
10.3.2 Forest plot . . . 80
10.3.3 Trim and Fill . . . 81
10.3.4 Baujat . . . 82

11 SF for LP stones >2cm **83**
11.1 Sort Data . . . 83
11.1.1 Overall Number of patients . . . 84
11.1.2 SF - Number of patients . . . 85
11.1.3 NSF - Number of patients . . . 86
11.2 Summary forestplot for SF . . . 87
11.3 Meta-Analysis . . . 90
11.3.1 Result . . . 90
11.3.2 Forest plot . . . 91
11.3.3 Trim and Fill . . . 92

11.3.4 Baujat . . . 93

12 Summary Forest plot for Overall Findings 95

13 Angle cut-off 97
13.1 Sort data . . . 97
13.2 Summary table . . . 98

14 Operative Time 99
14.1 How many studies detail Operative time? . . . 99
14.2 Analyse Operative time . . . 100
14.2.1 Overall Number of patients . . . 101
14.2.2 SF - Number of patients . . . 102
14.2.3 NSF - Number of patients . . . 103
14.3 Summary forestplot for SF . . . 104
14.4 Meta-Analysis . . . 107
14.4.1 Result . . . 107
14.4.2 Forest plot . . . 108
14.4.3 Trim and Fill . . . 109
14.4.4 Baujat . . . 110

1 Setup

2 Data Exploration

2.1 Plot missing data

```r
plot_missing(lpa_data)
```

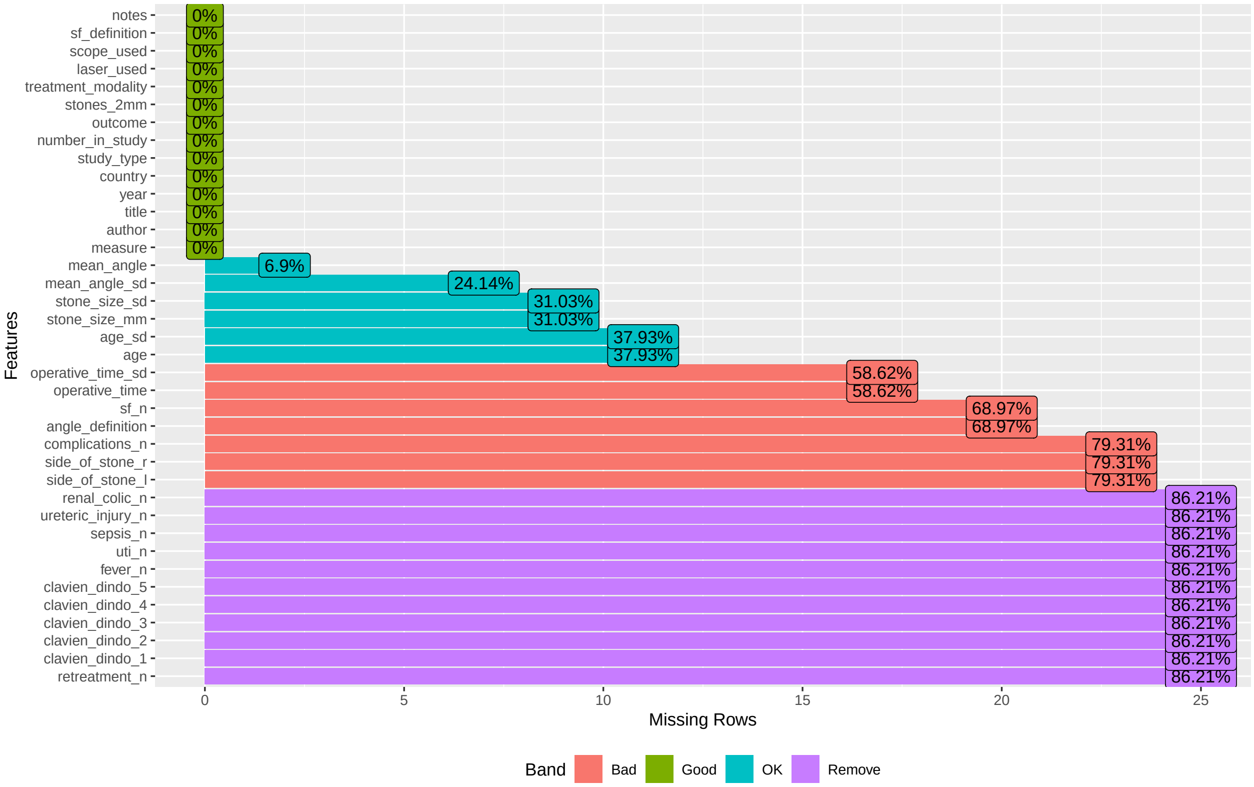

3 Summary Table

```r
lpa_data %>% subset(select = c(author,
                                year,
                                country,
                                study_type,
                                number_in_study,
                                outcome,
                                treatment_modality,
                                laser_used,
                                scope_used,
                                sf_definition)) %>% gt()
```

author	year	country	study_type	number_in_study	outcome	treatment_modality
Sari	2017	Turkey	Retrospective observational	103	SF	RIRS
Sari	2017	Turkey	Retrospective observational	29	NSF	RIRS
Resorlu	2012	Turkey	Retrospective observational	69	Steep	RIRS
Resorlu	2012	Turkey	Retrospective observational	19	Shallow	RIRS
Elbahnasy	1998	USA	Retrospective observational	8	SF	RIRS
Elbahnasy	1998	USA	Retrospective observational	5	NSF	RIRS
Resorlu	2012	Turkey	Retrospective observational	54	SF	RIRS
Resorlu	2012	Turkey	Retrospective observational	13	NSF	RIRS
Jessen	2014	Germany	Retrospective observational	98	SF	RIRS
Jessen	2014	Germany	Retrospective observational	13	NSF	RIRS
Geavlete	2008	Romania	Retrospective observational	4	Steep	RIRS
Geavlete	2008	Romania	Retrospective observational	35	Intermediate	RIRS
Geavlete	2008	Romania	Retrospective observational	8	Shallow	RIRS
Inoue	2015	Japan	Retrospective observational	22	SF	RIRS
Inoue	2015	Japan	Retrospective observational	8	NSF	RIRS
Tastemur	2022	Germany	Retrospective observational	105	SF	RIRS
Tastemur	2022	Germany	Retrospective observational	63	NSF	RIRS
Wang	2021	China	Retrospective observational	105	SF	RIRS
Wang	2021	China	Retrospective observational	42	NSF	RIRS
Richard	2020	France	Retrospective observational	308	SF	RIRS
Richard	2020	France	Retrospective observational	128	NSF	RIRS
Richard	2020	France	Retrospective observational	114	Steep	RIRS
Richard	2020	France	Retrospective observational	322	Shallow	RIRS
Karim	2019	UK	Retrospective observational	102	SF	RIRS
Karim	2019	UK	Retrospective observational	6	NSF	RIRS
Xiao	2017	China	Retrospective observational	69	SF	RIRS
Xiao	2017	China	Retrospective observational	76	NSF	RIRS
Kilicarslan	2015	Turkey	Retrospective observational	19	Steep	RIRS
Kilicarslan	2015	Turkey	Retrospective observational	17	Shallow	RIRS

4 SF cut-off

4.1 Sort Data

```
lpa_data$outcome <- as.factor(lpa_data$outcome)
lpa_data$stones_2mm <- as.factor(lpa_data$stones_2mm)

sf_data <- lpa_data %>% filter(measure != "ANGLE")
sf_data$mean_angle_sd <- as.numeric(sf_data$mean_angle_sd)
sf_data$mean_angle <- as.numeric(sf_data$mean_angle)

sf_data <- sf_data %>% drop_na(mean_angle_sd)

sf_data_nsf <- sf_data %>% filter(outcome !="SF") %>% mutate("mean_angle_nsf" = mean_angle,
                                                         "mean_angle_sd_nsf" = mean_angle_sd,
                                                         "n.e" = number_in_study)
sf_data_sf <- sf_data %>% filter(outcome !="NSF") %>% mutate("mean_angle_sf" = mean_angle,
                                                         "mean_angle_sd_sf" = mean_angle_sd,
                                                         "n.c" = number_in_study)

sf_data_reconfig <- cbind(sf_data_nsf, sf_data_sf)
sf_data_reconfig$mean_angle_nsf<-as.numeric(sf_data_reconfig$mean_angle_nsf)
sf_data_reconfig$mean_angle_sd_nsf<-as.numeric(sf_data_reconfig$mean_angle_sd_nsf)
sf_data_reconfig$mean_angle_sf<-as.numeric(sf_data_reconfig$mean_angle_sf)
sf_data_reconfig$mean_angle_sd_sf<-as.numeric(sf_data_reconfig$mean_angle_sd_sf)
sf_data_reconfig$n.e<-as.numeric(sf_data_reconfig$n.e)
sf_data_reconfig$n.c<-as.numeric(sf_data_reconfig$n.c)
```

4.1.1 Overall Number of patients

```r
sum(sf_data$number_in_study)
```

```
## [1] 1357
```

4.1.2 SF - Number of patients

```
sum(sf_data_sf$number_in_study)
```

```
## [1] 974
```

4.1.3 NSF - Number of patients

```r
sum(sf_data_nsf$number_in_study)
```

```
## [1] 383
```

4.2 Summary forestplot for SF

Above 67o all patients are stone free Below 30o no patients are stone free

These cut-offs fit with Kilicarslan (>70) and Geavlete (<30) respectively (see below)

By excluding Sari et al., we move the angle at which all patients become SF to ~60 Need to check how SF defined - is definition accounting for difference in SFR between Sari and remainder?

```
sf_data <- sf_data %>% mutate("lower" = mean_angle - mean_angle_sd,
                              "upper" = mean_angle + mean_angle_sd,
                              "angle" = mean_angle)

sf_data$lower <- as.numeric(sf_data$lower)
sf_data$upper <- as.numeric(sf_data$upper)
sf_data$angle <- as.numeric(sf_data$angle)
sf_data$outcome <- as.factor(sf_data$outcome)

sf_data <- sf_data %>% mutate(stud_lab = paste(author,
                                               year,
                                               sep = ", "))

sf_data_sf_mean <- metamean(n = number_in_study,
                  mean = mean_angle,
                  sd = mean_angle_sd,
                  studlab = author,
                  data = subset(sf_data,
                                outcome == "SF"),
                  sm = "MRAW",
                  fixed = FALSE,
                  random = TRUE,
                  method.tau = "REML",
                  hakn = TRUE,
                  title = "SF Mean")
sf_data_nsf_mean <-metamean(n = number_in_study,
                  mean = mean_angle,
                  sd = mean_angle_sd,
                  studlab = author,
                  data = subset(sf_data,
                                outcome == "NSF"),
                  sm = "MRAW",
                  fixed = FALSE,
                  random = TRUE,
                  method.tau = "REML",
                  hakn = TRUE,
                  title = "NSF Mean")

sf_data_minimised <- sf_data %>% subset(select = c(stud_lab,
                                                   outcome,
                                                   angle,
                                                   lower,
                                                   upper)) %>% rbind(c(
                                                     "Overall",
                                                     "SF",
                                                     round(sf_data_sf_mean$TE.random),
                                                     round(sf_data_sf_mean$lower.random),
```

```r
                                                               round(sf_data_sf_mean$upper.random)
                                                             )) %>% rbind(c(
                                                               "Overall",
                                                               "NSF",
                                                               round(sf_data_nsf_mean$TE.random),
                                                               round(sf_data_nsf_mean$lower.random),
                                                               round(sf_data_nsf_mean$upper.random)
                                                             ))

sf_data_minimised$outcome <- factor(sf_data_minimised$outcome,
                                    levels = c("SF",
                                               "NSF"))
sf_data_minimised$angle<- as.numeric(sf_data_minimised$angle)
sf_data_minimised$lower<- as.numeric(sf_data_minimised$lower)
sf_data_minimised$upper<- as.numeric(sf_data_minimised$upper)
sf_data_minimised$stud_lab <- as.character(sf_data_minimised$stud_lab)

sf_data_minimised %>% group_by(outcome) %>% forestplot(
  mean = angle,
  lower = lower,
  upper = upper,
  labeltext = stud_lab,
  fn.ci_norm = c(fpDrawNormalCI, fpDrawCircleCI),
  zero = 67,
  vertices = TRUE,
  cex = 2,
  lineheight = "auto",
  xlab = "Infundibulopelvic Angle (Degrees)",
  xticks = c(0, 10, 20, 30, 40, 50, 60, 70, 80, 90, 100),
) %>% fp_set_style(
  box = c("blue", "darkred"),
  line = "black",
  txt_gp = fpTxtGp(
    ticks = gpar(fontfamily = "", cex = 1),
    xlab  = gpar(fontfamily = "", cex = 1)
  )
) %>% fp_add_lines("steelblue") %>%
  fp_add_header("Outcome") %>% fp_set_zebra_style("#EFEFEFEF")
```

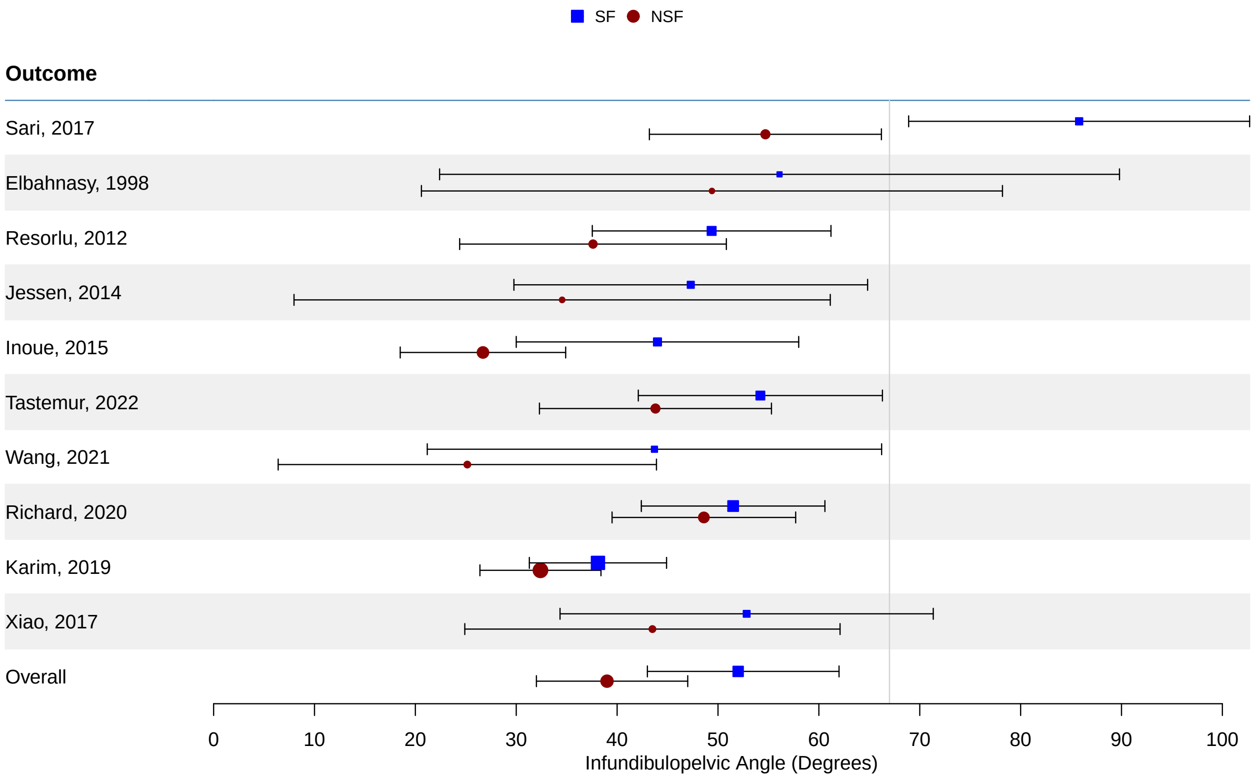

SF
NSF
Outcome
Sari, 2017
Elbahnasy, 1998
Resorlu, 2012
Jessen, 2014
Inoue, 2015
Tastemur, 2022
Wang, 2021
Richard, 2020
Karim, 2019
Xiao, 2017
Overall
0
10
20
30
40
50
60
70
80
90
100
Infundibulopelvic Angle (Degrees)

4.3 Meta-Analysis

4.3.1 Result

```r
sf_rma <- metacont(data = sf_data_reconfig,
                   mean.e = mean_angle_nsf,
                   sd.e = mean_angle_sd_nsf,
                   n.e = n.e,
                   mean.c = mean_angle_sf,
                   sd.c = mean_angle_sd_sf,
                   n.c = n.c,
                   studlab = paste(author, year, sep = ", ")
                   )
sf_rma
```

```
## Number of studies: k = 10
## Number of observations: o = 1357
##
##                              MD              95%-CI      z  p-value
## Common effect model   -7.8503 [ -9.2393; -6.4613] -11.08 < 0.0001
## Random effects model -12.9748 [-18.7108; -7.2388]  -4.43 < 0.0001
##
## Quantifying heterogeneity:
##  tau^2 = 66.5696 [22.9487; 204.4927]; tau = 8.1590 [4.7905; 14.3001]
##  I^2 = 92.4% [88.1%; 95.1%]; H = 3.63 [2.90; 4.54]
##
## Test of heterogeneity:
##       Q d.f.  p-value
##  118.49    9 < 0.0001
##
## Details on meta-analytical method:
## - Inverse variance method
## - Restricted maximum-likelihood estimator for tau^2
## - Q-Profile method for confidence interval of tau^2 and tau
```

4.3.2 Forest plot

```r
forest(sf_rma,
       sortvar = TE)
```

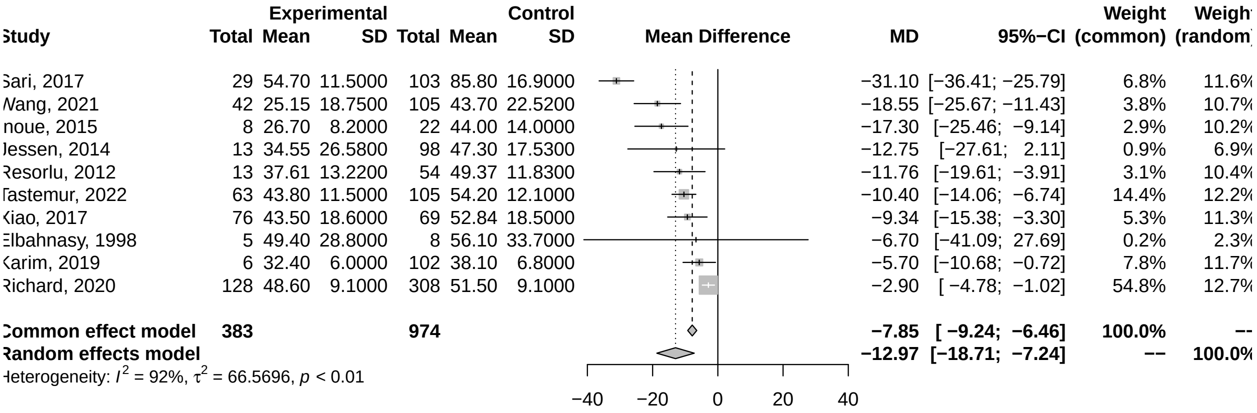

4.3.3 Trim and Fill

```
trimfill(sf_rma)
```

```
## Number of studies: k = 15 (with 5 added studies)
## Number of observations: o = 1844
##
##                              MD             95%-CI     z p-value
## Random effects model -5.5804 [-12.5468; 1.3860] -1.57  0.1164
##
## Quantifying heterogeneity:
##  tau^2 = 165.7400 [77.3354; 400.3065]; tau = 12.8740 [8.7941; 20.0077]
##  I^2 = 94.5% [92.4%; 96.1%]; H = 4.28 [3.64; 5.04]
##
## Test of heterogeneity:
##       Q d.f.  p-value
##  256.61   14 < 0.0001
##
## Details on meta-analytical method:
## - Inverse variance method
## - Restricted maximum-likelihood estimator for tau^2
## - Q-Profile method for confidence interval of tau^2 and tau
## - Trim-and-fill method to adjust for funnel plot asymmetry (L-estimator)
```

```
funnel(trimfill(sf_rma))
```

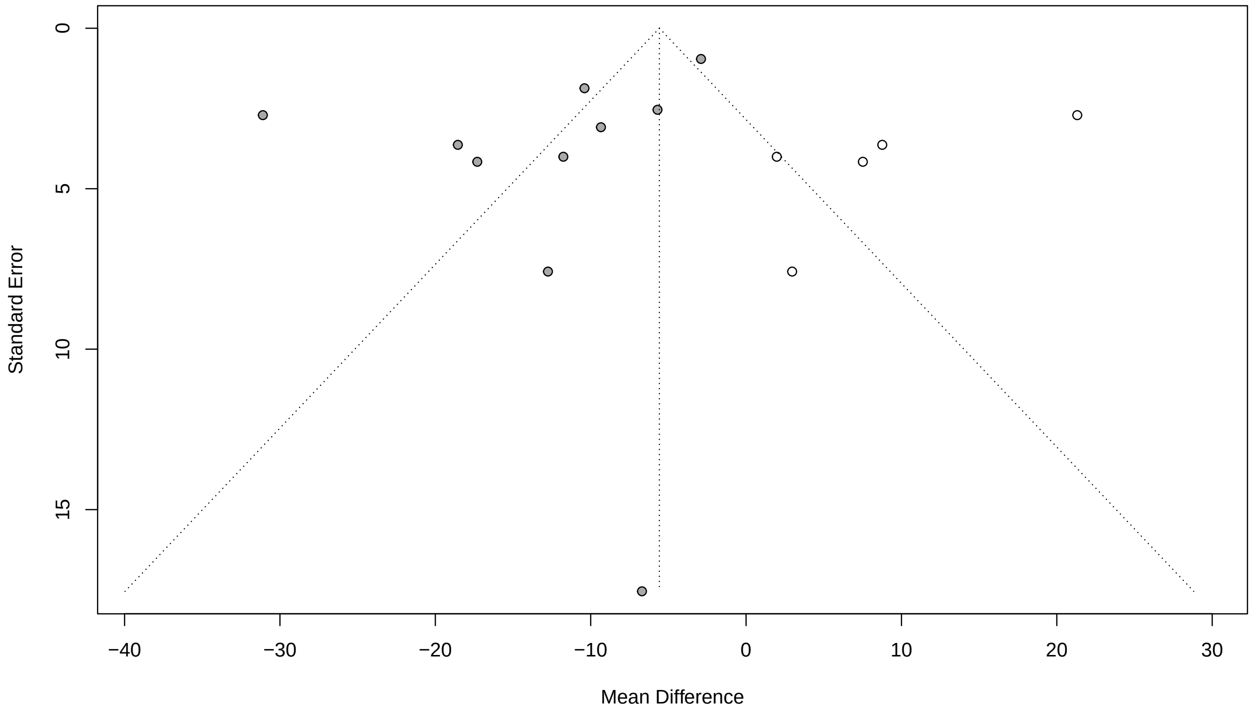


```
forest(trimfill(sf_rma),
       sortvar = TE)
```

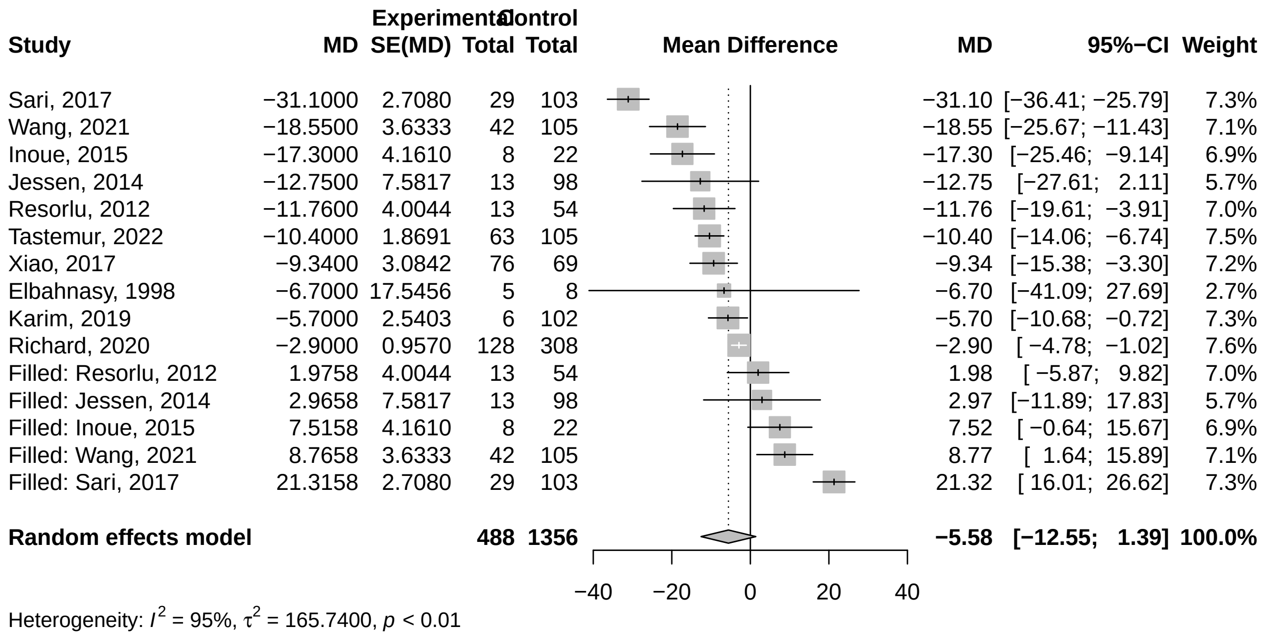

4.3.4 Baujat

```
baujat(sf_rma)
```

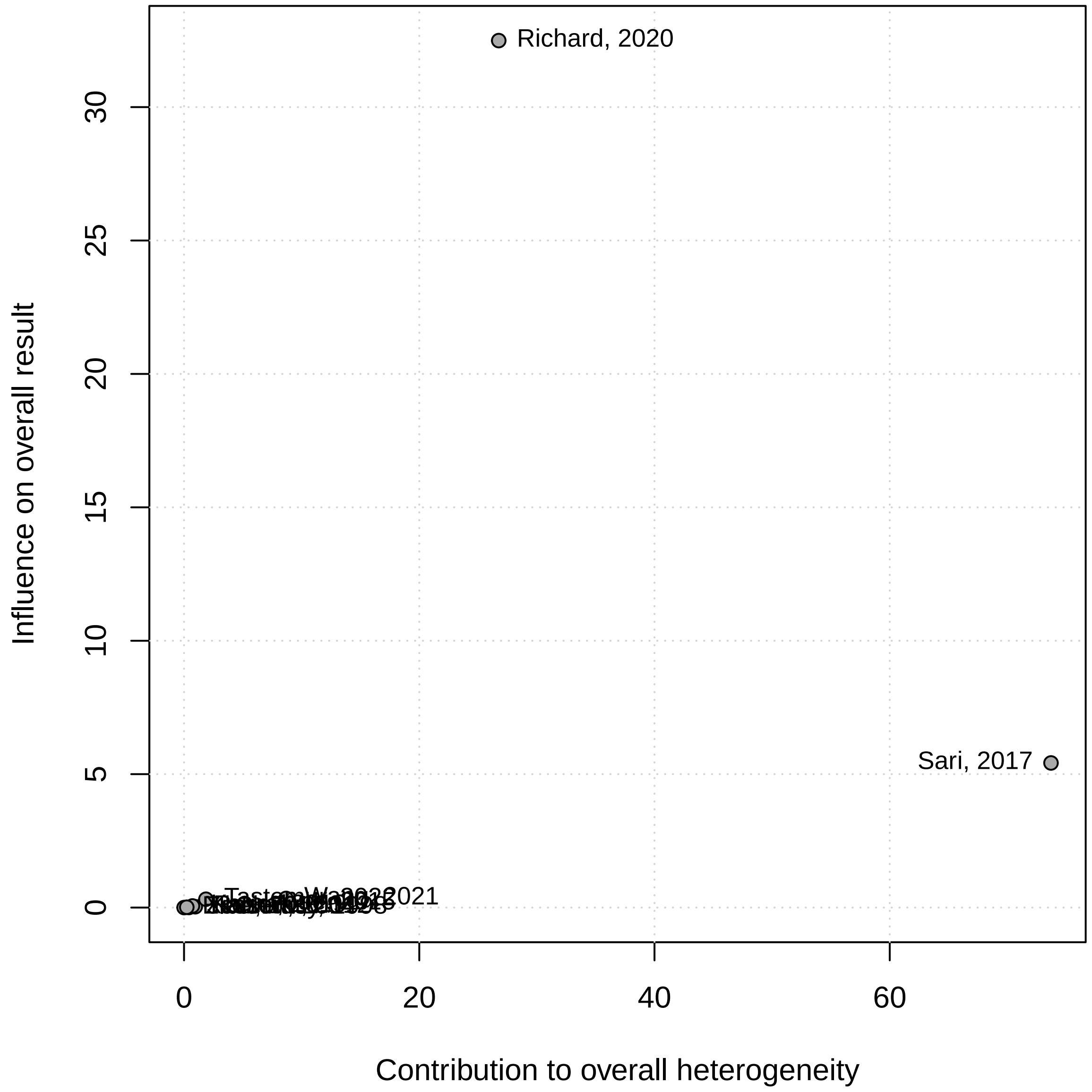

Richard, 2020
Sari, 2017
Influence on overall result
Contribution to overall heterogeneity
0
5
10
15
20
25
30
0
20
40
60

5 Exclude Sari

5.0.1 Summary forest plot

```
sf_data2 <- sf_data %>% subset(author != "Sari")

sf_data_nsf2 <- sf_data2 %>% filter(outcome !="SF") %>% mutate("mean_angle_nsf" = mean_angle,
                                                        "mean_angle_sd_nsf" = mean_angle_sd,
                                                        "n.e" = number_in_study)
sf_data_sf2 <- sf_data2 %>% filter(outcome !="NSF") %>% mutate("mean_angle_sf" = mean_angle,
                                                        "mean_angle_sd_sf" = mean_angle_sd,
                                                        "n.c" = number_in_study)

sf_data_reconfig2 <- cbind(sf_data_nsf2, sf_data_sf2)
sf_data_reconfig2$mean_angle_nsf<-as.numeric(sf_data_reconfig2$mean_angle_nsf)
sf_data_reconfig2$mean_angle_sd_nsf<-as.numeric(sf_data_reconfig2$mean_angle_sd_nsf)
sf_data_reconfig2$mean_angle_sf<-as.numeric(sf_data_reconfig2$mean_angle_sf)
sf_data_reconfig2$mean_angle_sd_sf<-as.numeric(sf_data_reconfig2$mean_angle_sd_sf)
sf_data_reconfig2$n.e<-as.numeric(sf_data_reconfig2$n.e)
sf_data_reconfig2$n.c<-as.numeric(sf_data_reconfig2$n.c)

sf_data_sf_mean_exc_Sari <- metamean(n = number_in_study,
                   mean = mean_angle,
                   sd = mean_angle_sd,
                   studlab = author,
                   data = (subset(sf_data,
                                 outcome == "SF") %>% subset(author != "Sari")),
                   sm = "MRAW",
                   fixed = FALSE,
                   random = TRUE,
                   method.tau = "REML",
                   hakn = TRUE,
                   title = "SF Mean")
sf_data_nsf_mean_exc_Sari <-metamean(n = number_in_study,
                   mean = mean_angle,
                   sd = mean_angle_sd,
                   studlab = author,
                   data = (subset(sf_data,
                                 outcome == "NSF") %>% subset(author != "Sari")),
                   sm = "MRAW",
                   fixed = FALSE,
                   random = TRUE,
                   method.tau = "REML",
                   hakn = TRUE,
                   title = "NSF Mean")

sf_data_minimised_exc_Sari <- sf_data %>% subset(author != "Sari") %>% subset(select = c(stud_lab,
                                                   outcome,
                                                   angle,
                                                   lower,
                                                   upper)) %>% rbind(c(
                                                     "Overall",
                                                     "SF",
                                                     round(sf_data_sf_mean_exc_Sari$TE.random),
```

```
                                                    round(sf_data_sf_mean_exc_Sari$lower.random),
                                                    round(sf_data_sf_mean_exc_Sari$upper.random)
                                                  )) %>% rbind(c(
                                                    "Overall",
                                                    "NSF",
                                                    round(sf_data_nsf_mean_exc_Sari$TE.random),
                                                    round(sf_data_nsf_mean_exc_Sari$lower.random),
                                                    round(sf_data_nsf_mean_exc_Sari$upper.random)
                                                  ))

sf_data_minimised_exc_Sari$outcome <- factor(sf_data_minimised_exc_Sari$outcome,
                                    levels = c("SF",
                                               "NSF"))
sf_data_minimised_exc_Sari$angle<- as.numeric(sf_data_minimised_exc_Sari$angle)
sf_data_minimised_exc_Sari$lower<- as.numeric(sf_data_minimised_exc_Sari$lower)
sf_data_minimised_exc_Sari$upper<- as.numeric(sf_data_minimised_exc_Sari$upper)

sf_data_minimised_exc_Sari %>% group_by(outcome) %>% forestplot(
  mean = angle,
  lower = lower,
  upper = upper,
  labeltext = stud_lab,
  fn.ci_norm = c(fpDrawNormalCI, fpDrawCircleCI),
  zero = 67,
  vertices = TRUE,
  cex = 2,
  lineheight = "auto",
  xlab = "Infundibulopelvic Angle (Degrees)",
  xticks = c(0, 10, 20, 30, 40, 50, 60, 70, 80, 90, 100),
) %>% fp_set_style(
  box = c("blue", "darkred"),
  line = "black",
  txt_gp = fpTxtGp(
    ticks = gpar(fontfamily = "", cex = 1),
    xlab  = gpar(fontfamily = "", cex = 1)
  )
) %>% fp_add_lines("steelblue") %>%
  fp_add_header("Outcome") %>% fp_set_zebra_style("#EFEFEFEF")
```

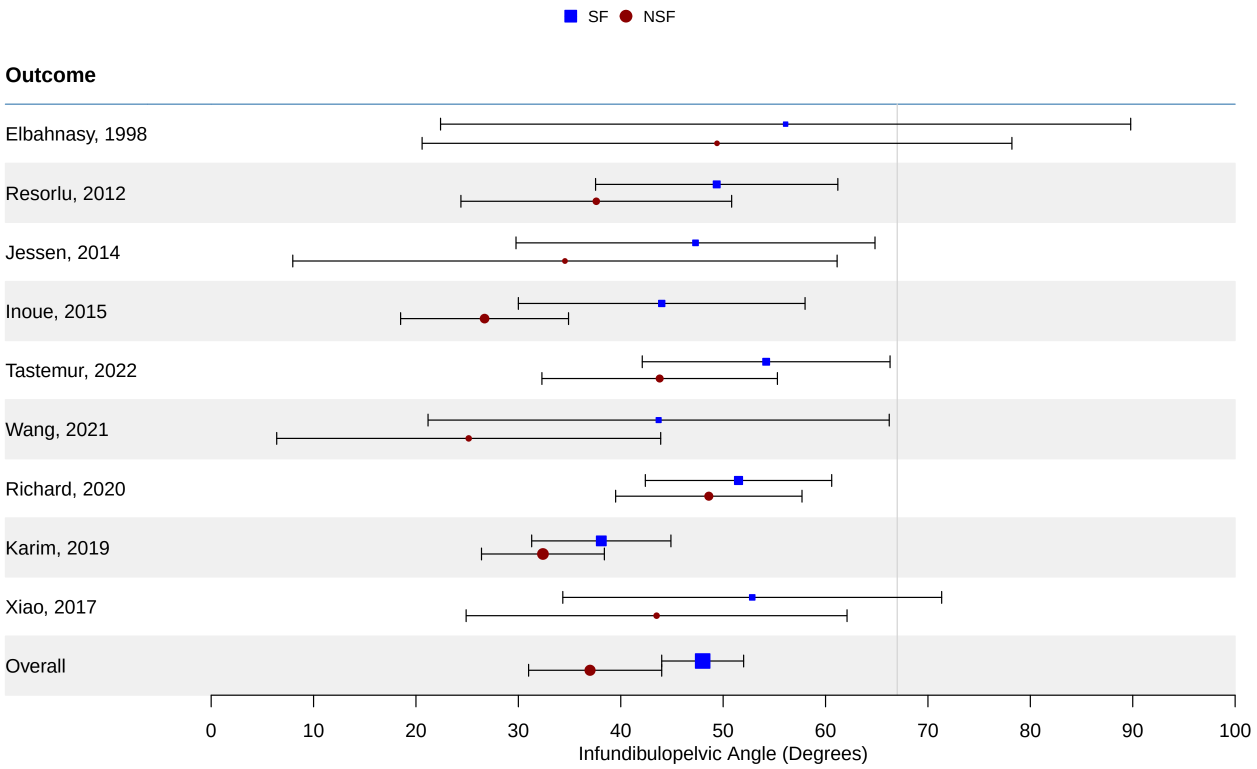
SF
NSF
Outcome
Elbahnasy, 1998
Resorlu, 2012
Jessen, 2014
Inoue, 2015
Tastemur, 2022
Wang, 2021
Richard, 2020
Karim, 2019
Xiao, 2017
Overall
0
10
20
30
40
50
60
70
80
90
100
Infundibulopelvic Angle (Degrees)

5.0.2 Result

```
sf_rma2 <- metacont(data = sf_data_reconfig2,
                   mean.e = mean_angle_nsf,
                   sd.e = mean_angle_sd_nsf,
                   n.e = n.e,
                   mean.c = mean_angle_sf,
                   sd.c = mean_angle_sd_sf,
                   n.c = n.c,
                   studlab = paste(author, year, sep = ", ")
                   )
sf_rma2
```

```
## Number of studies: k = 9
## Number of observations: o = 1225
##
##                             MD              95%-CI     z  p-value
## Common effect model   -6.1409 [ -7.5801; -4.7018] -8.36 < 0.0001
## Random effects model -10.1528 [-14.1501; -6.1554] -4.98 < 0.0001
##
## Quantifying heterogeneity:
##  tau^2 = 22.6495 [4.2235; 83.1089]; tau = 4.7591 [2.0551; 9.1164]
##  I^2 = 79.7% [62.0%; 89.1%]; H = 2.22 [1.62; 3.03]
##
## Test of heterogeneity:
##      Q d.f.  p-value
##  39.35    8 < 0.0001
##
## Details on meta-analytical method:
## - Inverse variance method
## - Restricted maximum-likelihood estimator for tau^2
## - Q-Profile method for confidence interval of tau^2 and tau
```

5.0.3 Forest plot

```r
forest(sf_rma2,
       sortvar = TE)
```

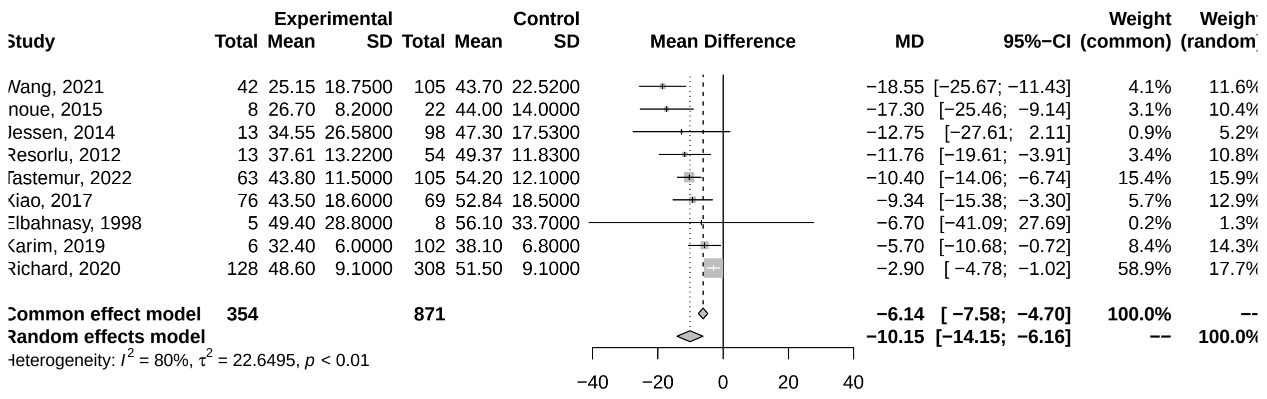

5.0.4 Trim and Fill

```r
trimfill(sf_rma2)
```

```
## Number of studies: k = 14 (with 5 added studies)
## Number of observations: o = 1748
##
##                              MD             95%-CI     z p-value
## Random effects model -4.3252 [-9.5926; 0.9421] -1.61  0.1075
##
## Quantifying heterogeneity:
##  tau^2 = 80.3798 [32.5165; 225.3008]; tau = 8.9655 [5.7023; 15.0100]
##  I^2 = 86.4% [78.8%; 91.3%]; H = 2.71 [2.17; 3.38]
##
## Test of heterogeneity:
##      Q d.f.  p-value
##  95.60   13 < 0.0001
##
## Details on meta-analytical method:
## - Inverse variance method
## - Restricted maximum-likelihood estimator for tau^2
## - Q-Profile method for confidence interval of tau^2 and tau
## - Trim-and-fill method to adjust for funnel plot asymmetry (L-estimator)
```

```r
funnel(trimfill(sf_rma2))
```

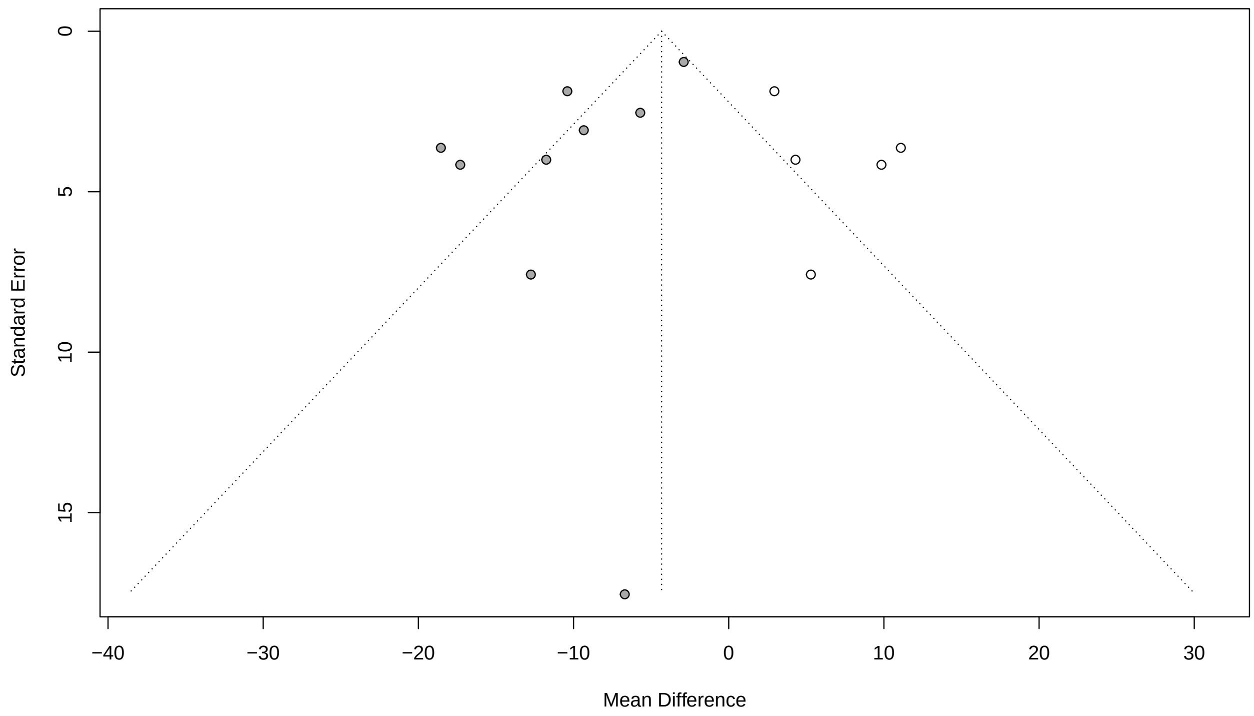


```r
forest(trimfill(sf_rma2))
```

Study	MD	SE(MD)	Experimental Total	Control Total	MD	95%−CI	Weight
Elbahnasy, 1998	−6.7000	17.5456	5	8	−6.70	[−41.09; 27.69]	1.9%
Resorlu, 2012	−11.7600	4.0044	13	54	−11.76	[−19.61; −3.91]	7.5%
Jessen, 2014	−12.7500	7.5817	13	98	−12.75	[−27.61; 2.11]	5.2%
Inoue, 2015	−17.3000	4.1610	8	22	−17.30	[−25.46; −9.14]	7.4%
Tastemur, 2022	−10.4000	1.8691	63	105	−10.40	[−14.06; −6.74]	8.6%
Wang, 2021	−18.5500	3.6333	42	105	−18.55	[−25.67; −11.43]	7.7%
Richard, 2020	−2.9000	0.9570	128	308	−2.90	[−4.78; −1.02]	8.9%
Karim, 2019	−5.7000	2.5403	6	102	−5.70	[−10.68; −0.72]	8.3%
Xiao, 2017	−9.3400	3.0842	76	69	−9.34	[−15.38; −3.30]	8.0%
Filled: Tastemur, 2022	2.9424	1.8691	63	105	2.94	[−0.72; 6.61]	8.6%
Filled: Resorlu, 2012	4.3024	4.0044	13	54	4.30	[−3.55; 12.15]	7.5%
Filled: Jessen, 2014	5.2924	7.5817	13	98	5.29	[−9.57; 20.15]	5.2%
Filled: Inoue, 2015	9.8424	4.1610	8	22	9.84	[1.69; 18.00]	7.4%
Filled: Wang, 2021	11.0924	3.6333	42	105	11.09	[3.97; 18.21]	7.7%
**Random effects model**			**493**	**1255**	**−4.33**	**[−9.59; 0.94]**	**100.0%**

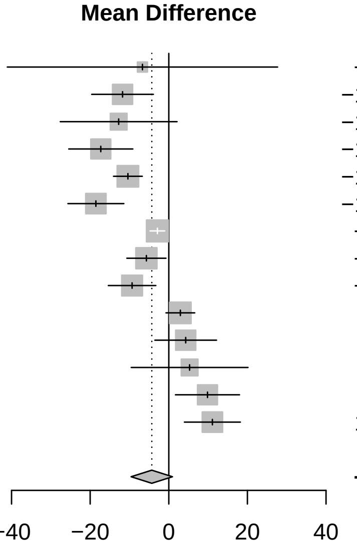

Heterogeneity: $I^2 = 86\%$, $\tau^2 = 80.3798$, $p < 0.01$

5.0.5 Baujat

```
baujat(sf_rma2)
```

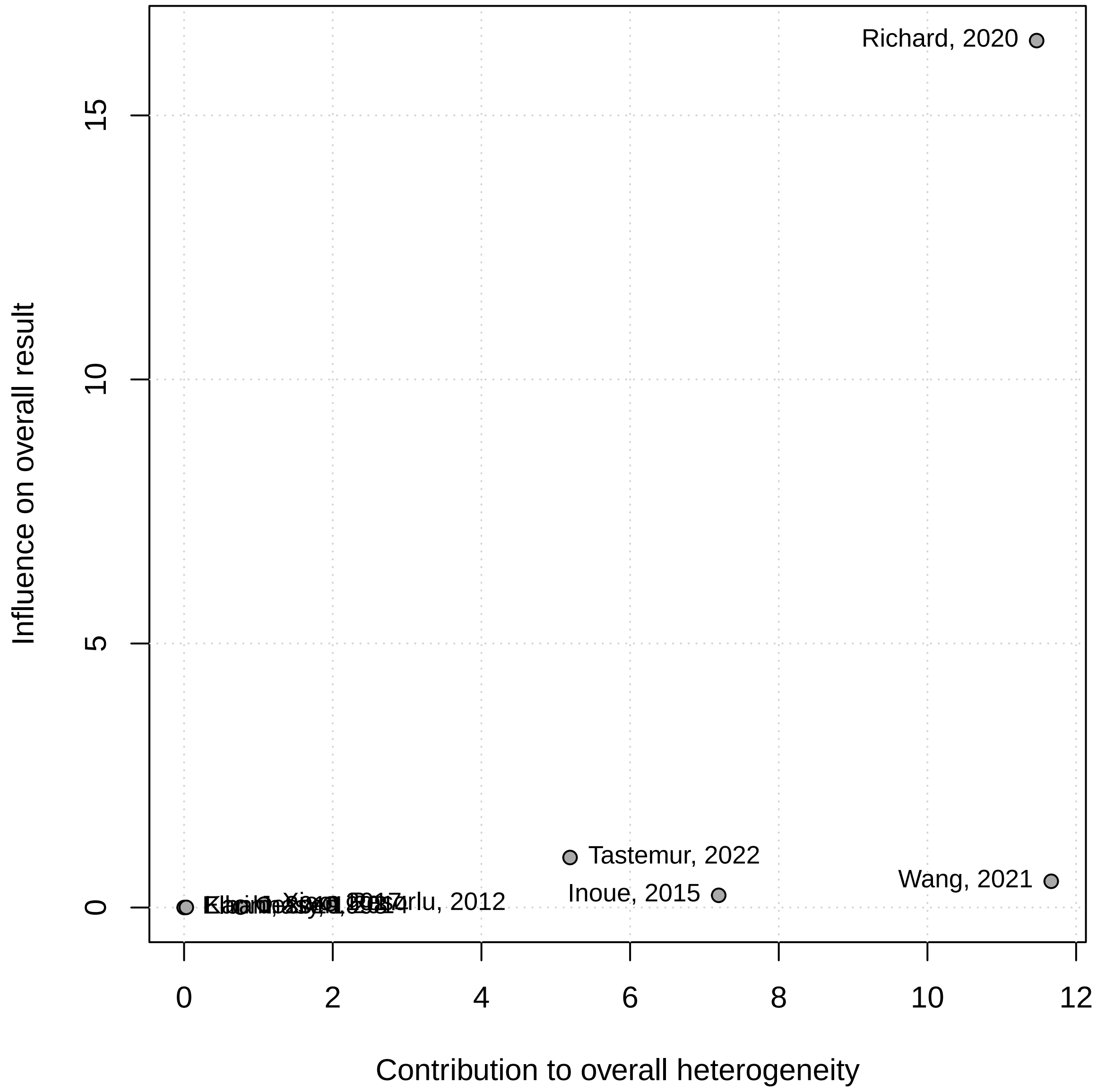
Richard, 2020
Tastemur, 2022
Inoue, 2015
Wang, 2021
Influence on overall result
Contribution to overall heterogeneity
0
5
10
15
0
2
4
6
8
10
12

6 SF cut-off for Flex X2

6.1 Sort Data

```r
sf_data$scope_used <- as.factor(sf_data$scope_used)

sf_data_x2_1 <- sf_data %>% subset(scope_used == "Flex X2")
sf_data_x2_2 <- sf_data %>% subset(scope_used == "Flex X2 or Olympus")
sf_data_x2 <- rbind(sf_data_x2_1,
                    sf_data_x2_2)

sf_data_x2_nsf <- sf_data_x2 %>% filter(outcome !="SF") %>% mutate("mean_angle_nsf" = mean_angle,
                                                       "mean_angle_sd_nsf" = mean_angle_sd,
                                                       "n.e" = number_in_study)
sf_data_x2_sf <- sf_data_x2 %>% filter(outcome !="NSF") %>% mutate("mean_angle_sf" = mean_angle,
                                                       "mean_angle_sd_sf" = mean_angle_sd,
                                                       "n.c" = number_in_study)

sf_data_x2_reconfig <- cbind(sf_data_x2_nsf, sf_data_x2_sf)
sf_data_x2_reconfig$mean_angle_nsf<-as.numeric(sf_data_x2_reconfig$mean_angle_nsf)
sf_data_x2_reconfig$mean_angle_sd_nsf<-as.numeric(sf_data_x2_reconfig$mean_angle_sd_nsf)
sf_data_x2_reconfig$mean_angle_sf<-as.numeric(sf_data_x2_reconfig$mean_angle_sf)
sf_data_x2_reconfig$mean_angle_sd_sf<-as.numeric(sf_data_x2_reconfig$mean_angle_sd_sf)
sf_data_x2_reconfig$n.e<-as.numeric(sf_data_x2_reconfig$n.e)
sf_data_x2_reconfig$n.c<-as.numeric(sf_data_x2_reconfig$n.c)
```

6.1.1 Overall Number of patients

```
sum(sf_data_x2$number_in_study)
```

```
## [1] 519
```

6.1.2 SF - Number of patients

```
sum(sf_data_x2_sf$number_in_study)
```

```
## [1] 408
```

6.1.3 NSF - Number of patients

```
sum(sf_data_x2_nsf$number_in_study)
```

```
## [1] 111
```

6.2 Summary foresplot for SF

Above 67o all patients are stone free Below 30o no patients are stone free

These cut-offs fit with Kilicarslan (>70) and Geavlete (<30) respectively (see below)

By excluding Sari et al., we move the angle at which all patients become SF to ~60 Need to check how SF defined - is definition accounting for difference in SFR between Sari and remainder?

```
sf_data_x2 <- sf_data_x2 %>% mutate("lower" = mean_angle - mean_angle_sd,
                             "upper" = mean_angle + mean_angle_sd,
                             "angle" = mean_angle)

sf_data_x2$lower <- as.numeric(sf_data_x2$lower)
sf_data_x2$upper <- as.numeric(sf_data_x2$upper)
sf_data_x2$angle <- as.numeric(sf_data_x2$angle)
sf_data_x2$outcome <- as.factor(sf_data_x2$outcome)

sf_data_x2 <- sf_data_x2 %>% mutate(stud_lab = paste(author,
                                                 year,
                                                 sep = ", ")) %>% subset(author != "Sari")



sf_data_sf_x2 <- metamean(n = number_in_study,
                   mean = mean_angle,
                   sd = mean_angle_sd,
                   studlab = author,
                   data = subset(sf_data_x2,
                                 outcome == "SF"),
                   sm = "MRAW",
                   fixed = FALSE,
                   random = TRUE,
                   method.tau = "REML",
                   hakn = TRUE,
                   title = "SF Mean")
sf_data_nsf_x2 <-metamean(n = number_in_study,
                   mean = mean_angle,
                   sd = mean_angle_sd,
                   studlab = author,
                   data = subset(sf_data_x2,
                                 outcome == "NSF"),
                   sm = "MRAW",
                   fixed = FALSE,
                   random = TRUE,
                   method.tau = "REML",
                   hakn = TRUE,
                   title = "NSF Mean")

sf_data_x2_minimised <- sf_data_x2 %>% subset(select = c(stud_lab,
                                                     outcome,
                                                     angle,
                                                     lower,
                                                     upper)) %>% rbind(c(
                                                       "Overall",
                                                       "SF",
```

```
                                                    round(sf_data_sf_x2$TE.random),
                                                    round(sf_data_sf_x2$lower.random),
                                                    round(sf_data_sf_x2$upper.random)
                                                  )) %>% rbind(c(
                                                    "Overall",
                                                    "NSF",
                                                    round(sf_data_nsf_x2$TE.random),
                                                    round(sf_data_nsf_x2$lower.random),
                                                    round(sf_data_nsf_x2$upper.random)
                                                  ))

sf_data_x2_minimised$outcome <- factor(sf_data_x2_minimised$outcome,
                                   levels = c("SF",
                                              "NSF"))
sf_data_x2_minimised$angle<- as.numeric(sf_data_x2_minimised$angle)
sf_data_x2_minimised$lower<- as.numeric(sf_data_x2_minimised$lower)
sf_data_x2_minimised$upper<- as.numeric(sf_data_x2_minimised$upper)

sf_data_x2_minimised %>% group_by(outcome) %>% forestplot(
  mean = angle,
  lower = lower,
  upper = upper,
  labeltext = stud_lab,
  fn.ci_norm = c(fpDrawNormalCI, fpDrawCircleCI),
  zero = 67,
  vertices = TRUE,
  cex = 2,
  lineheight = "auto",
  xlab = "Infundibulopelvic Angle (Degrees)",
  xticks = c(0, 10, 20, 30, 40, 50, 60, 70, 80, 90, 100),
) %>% fp_set_style(
  box = c("blue", "darkred"),
  line = "black",
  txt_gp = fpTxtGp(
    ticks = gpar(fontfamily = "", cex = 1),
    xlab  = gpar(fontfamily = "", cex = 1)
  )
) %>% fp_add_lines("steelblue") %>%
  fp_add_header("Outcome") %>% fp_set_zebra_style("#EFEFEFEF")
```

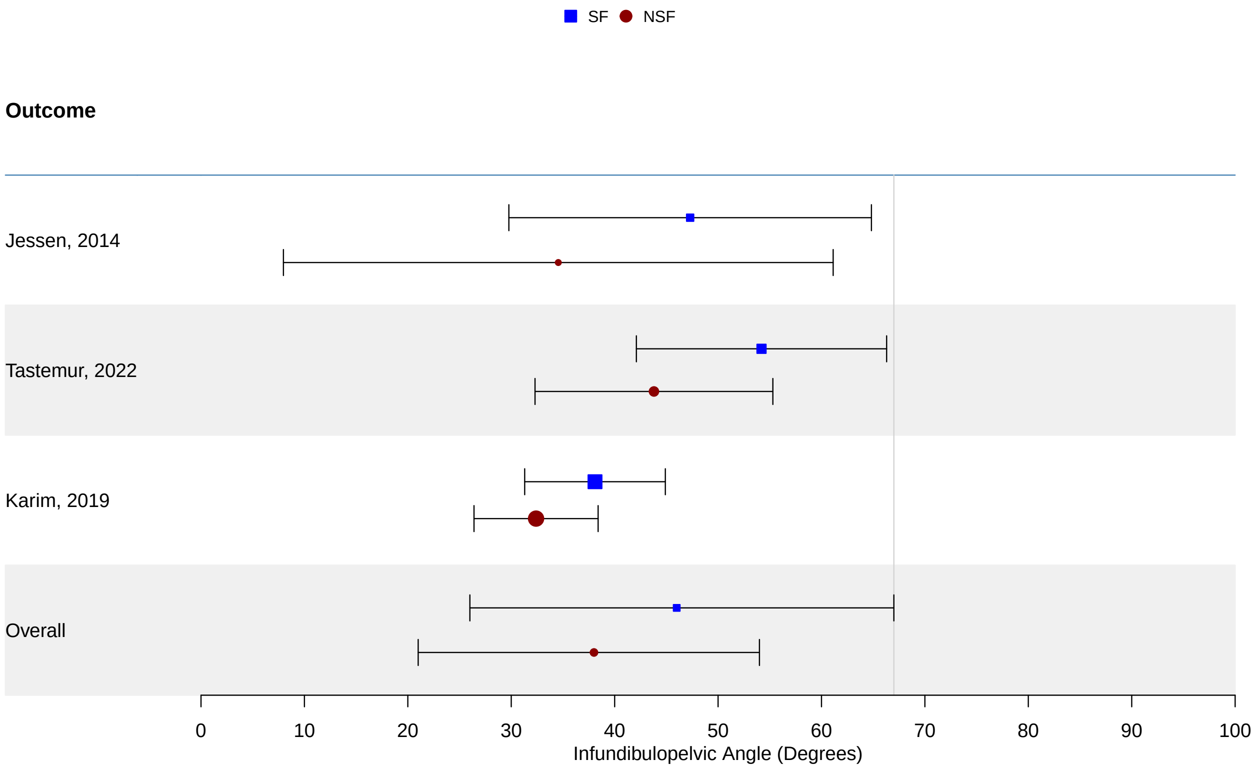
SF
NSF
Outcome
Jessen, 2014
Tastemur, 2022
Karim, 2019
Overall
0
10
20
30
40
50
60
70
80
90
100
Infundibulopelvic Angle (Degrees)

6.3 Meta-Analysis

6.3.1 Result

```
sf_x2_rma <- metacont(data = sf_data_x2_reconfig,
                   mean.e = mean_angle_nsf,
                   sd.e = mean_angle_sd_nsf,
                   n.e = n.e,
                   mean.c = mean_angle_sf,
                   sd.c = mean_angle_sd_sf,
                   n.c = n.c,
                   studlab = paste(author, year, sep = ", ")
                   )
sf_x2_rma
```

```
## Number of studies: k = 4
## Number of observations: o = 519
##
##                            MD              95%-CI      z  p-value
## Common effect model  -13.9890 [-16.5300; -11.4480] -10.79 < 0.0001
## Random effects model -15.0979 [-26.7329;  -3.4628]  -2.54   0.0110
##
## Quantifying heterogeneity:
##  tau^2 = 125.2427 [32.5916; >1252.4266]; tau = 11.1912 [5.7089; >35.3896]
##  I^2 = 94.5% [88.9%; 97.2%]; H = 4.25 [3.01; 6.02]
##
## Test of heterogeneity:
##      Q d.f.  p-value
##  54.29    3 < 0.0001
##
## Details on meta-analytical method:
## - Inverse variance method
## - Restricted maximum-likelihood estimator for tau^2
## - Q-Profile method for confidence interval of tau^2 and tau
```

6.3.2 Forest plot

```r
forest(sf_x2_rma,
       sortvar = TE)
```

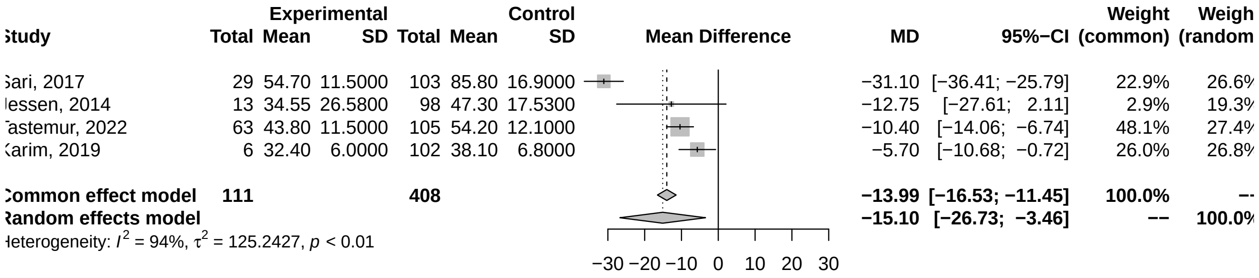

6.3.3 Trim and Fill

```
trimfill(sf_x2_rma)
```

```
## Number of studies: k = 4 (with 0 added studies)
## Number of observations: o = 519
##
##                              MD              95%-CI     z p-value
## Random effects model -15.0979 [-26.7329; -3.4628] -2.54  0.0110
##
## Quantifying heterogeneity:
##  tau^2 = 125.2427 [32.5916; >1252.4266]; tau = 11.1912 [5.7089; >35.3896]
##  I^2 = 94.5% [88.9%; 97.2%]; H = 4.25 [3.01; 6.02]
##
## Test of heterogeneity:
##      Q d.f.  p-value
##  54.29    3 < 0.0001
##
## Details on meta-analytical method:
## - Inverse variance method
## - Restricted maximum-likelihood estimator for tau^2
## - Q-Profile method for confidence interval of tau^2 and tau
## - Trim-and-fill method to adjust for funnel plot asymmetry (L-estimator)
```

```
funnel(trimfill(sf_x2_rma))
```

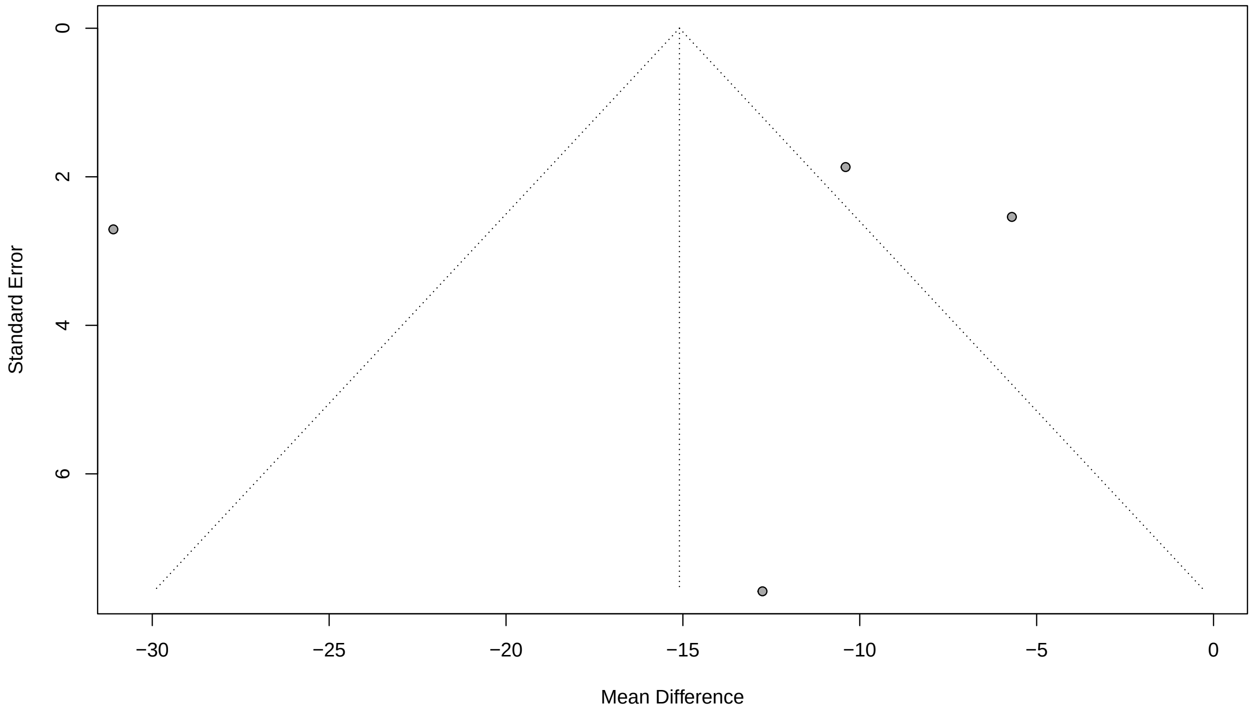


```
forest(trimfill(sf_x2_rma),
       sortvar = TE)
```

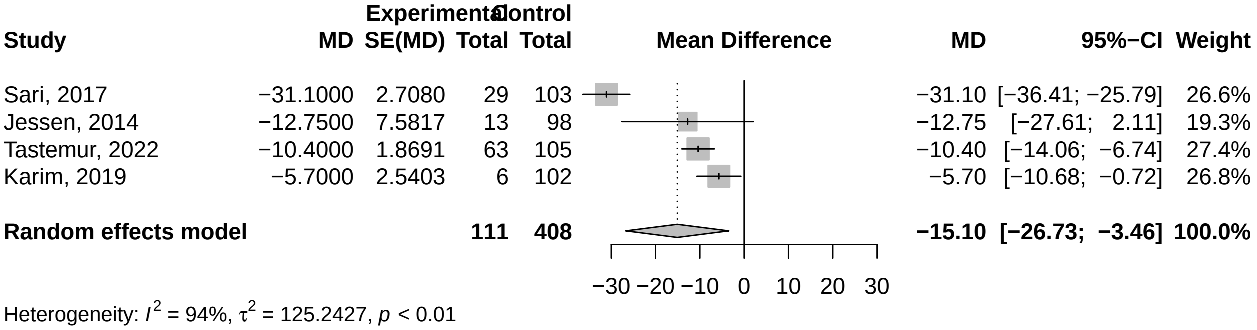

6.3.4 Baujat

```
baujat(sf_x2_rma)
```

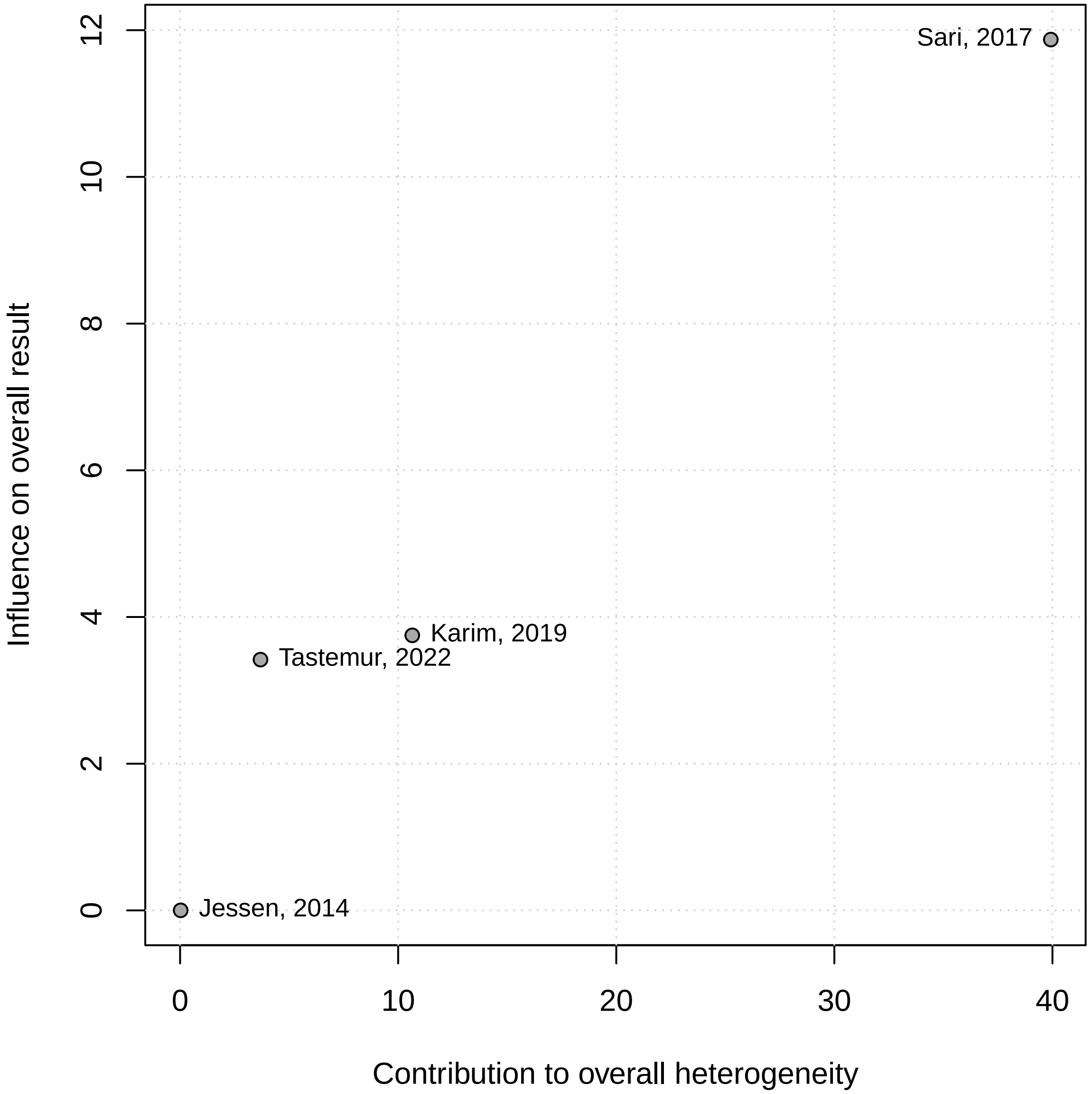

7 SF cut-off for Non Flex X2

7.1 Sort Data

```
sf_data$scope_used <- as.factor(sf_data$scope_used)

sf_data_non_x2_1 <- sf_data %>% subset(scope_used != "Flex X2")
sf_data_non_x2 <- sf_data_non_x2_1 %>% subset(scope_used != "Flex X2 or Olympus")


sf_data_non_x2_nsf <- sf_data_non_x2 %>% filter(outcome !="SF") %>% mutate("mean_angle_nsf" = mean_angl
                                                       "mean_angle_sd_nsf" = mean_angle_sd,
                                                       "n.e" = number_in_study)
sf_data_non_x2_sf <- sf_data_non_x2 %>% filter(outcome !="NSF") %>% mutate("mean_angle_sf" = mean_angle
                                                       "mean_angle_sd_sf" = mean_angle_sd,
                                                       "n.c" = number_in_study)

sf_data_non_x2_reconfig <- cbind(sf_data_non_x2_nsf, sf_data_non_x2_sf)
sf_data_non_x2_reconfig$mean_angle_nsf<-as.numeric(sf_data_non_x2_reconfig$mean_angle_nsf)
sf_data_non_x2_reconfig$mean_angle_sd_nsf<-as.numeric(sf_data_non_x2_reconfig$mean_angle_sd_nsf)
sf_data_non_x2_reconfig$mean_angle_sf<-as.numeric(sf_data_non_x2_reconfig$mean_angle_sf)
sf_data_non_x2_reconfig$mean_angle_sd_sf<-as.numeric(sf_data_non_x2_reconfig$mean_angle_sd_sf)
sf_data_non_x2_reconfig$n.e<-as.numeric(sf_data_non_x2_reconfig$n.e)
sf_data_non_x2_reconfig$n.c<-as.numeric(sf_data_non_x2_reconfig$n.c)
```

7.1.1 Overall Number of patients

```
sum(sf_data_non_x2$number_in_study)
```

```
## [1] 838
```

7.1.2 SF - Number of patients

```
sum(sf_data_non_x2_sf$number_in_study)
```

```
## [1] 566
```

7.1.3 NSF - Number of patients

```
sum(sf_data_non_x2_nsf$number_in_study)
```

```
## [1] 272
```

7.2 Summary forestplot for SF

Above 67o all patients are stone free Below 30o no patients are stone free

These cut-offs fit with Kilicarslan (>70) and Geavlete (<30) respectively (see below)

By excluding Sari et al., we move the angle at which all patients become SF to ~60 Need to check how SF defined - is definition accounting for difference in SFR between Sari and remainder?

```
sf_data_non_x2 <- sf_data_non_x2 %>% mutate("lower" = mean_angle - mean_angle_sd,
                              "upper" = mean_angle + mean_angle_sd,
                              "angle" = mean_angle)

sf_data_non_x2$lower <- as.numeric(sf_data_non_x2$lower)
sf_data_non_x2$upper <- as.numeric(sf_data_non_x2$upper)
sf_data_non_x2$angle <- as.numeric(sf_data_non_x2$angle)
sf_data_non_x2$outcome <- as.factor(sf_data_non_x2$outcome)

sf_data_non_x2 <- sf_data_non_x2 %>% mutate(stud_lab = paste(author,
                                               year,
                                               sep = ", "))

sf_data_sf_non_x2 <- metamean(n = number_in_study,
                   mean = mean_angle,
                   sd = mean_angle_sd,
                   studlab = author,
                   data = subset(sf_data_non_x2,
                                 outcome == "SF"),
                   sm = "MRAW",
                   fixed = FALSE,
                   random = TRUE,
                   method.tau = "REML",
                   hakn = TRUE,
                   title = "SF Mean")
sf_data_nsf_non_x2 <-metamean(n = number_in_study,
                   mean = mean_angle,
                   sd = mean_angle_sd,
                   studlab = author,
                   data = subset(sf_data_non_x2,
                                 outcome == "NSF"),
                   sm = "MRAW",
                   fixed = FALSE,
                   random = TRUE,
                   method.tau = "REML",
                   hakn = TRUE,
                   title = "NSF Mean")

sf_data_non_x2_minimised <- sf_data_non_x2 %>% subset(select = c(stud_lab,
                                                   outcome,
                                                   angle,
                                                   lower,
                                                   upper)) %>% rbind(c(
                                                     "Overall",
                                                     "SF",
                                                     round(sf_data_sf_non_x2$TE.random),
                                                     round(sf_data_sf_non_x2$lower.random),
```

```r
                                                          round(sf_data_sf_non_x2$upper.random)
                                                        )) %>% rbind(c(
                                                          "Overall",
                                                          "NSF",
                                                          round(sf_data_nsf_non_x2$TE.random),
                                                          round(sf_data_nsf_non_x2$lower.random),
                                                          round(sf_data_nsf_non_x2$upper.random)
                                                        ))

sf_data_non_x2_minimised$outcome <- factor(sf_data_non_x2_minimised$outcome,
                                    levels = c("SF",
                                               "NSF"))
sf_data_non_x2_minimised$angle<- as.numeric(sf_data_non_x2_minimised$angle)
sf_data_non_x2_minimised$lower<- as.numeric(sf_data_non_x2_minimised$lower)
sf_data_non_x2_minimised$upper<- as.numeric(sf_data_non_x2_minimised$upper)

sf_data_non_x2_minimised %>% group_by(outcome) %>% forestplot(
  mean = angle,
  lower = lower,
  upper = upper,
  labeltext = stud_lab,
  fn.ci_norm = c(fpDrawNormalCI, fpDrawCircleCI),
  zero = 67,
  vertices = TRUE,
  cex = 2,
  lineheight = "auto",
  xlab = "Infundibulopelvic Angle (Degrees)",
  xticks = c(0, 10, 20, 30, 40, 50, 60, 70, 80, 90, 100),
) %>% fp_set_style(
  box = c("blue", "darkred"),
  line = "black",
  txt_gp = fpTxtGp(
    ticks = gpar(fontfamily = "", cex = 1),
    xlab  = gpar(fontfamily = "", cex = 1)
  )
) %>% fp_add_lines("steelblue") %>%
  fp_add_header("Outcome") %>% fp_set_zebra_style("#EFEFEFEF")
```

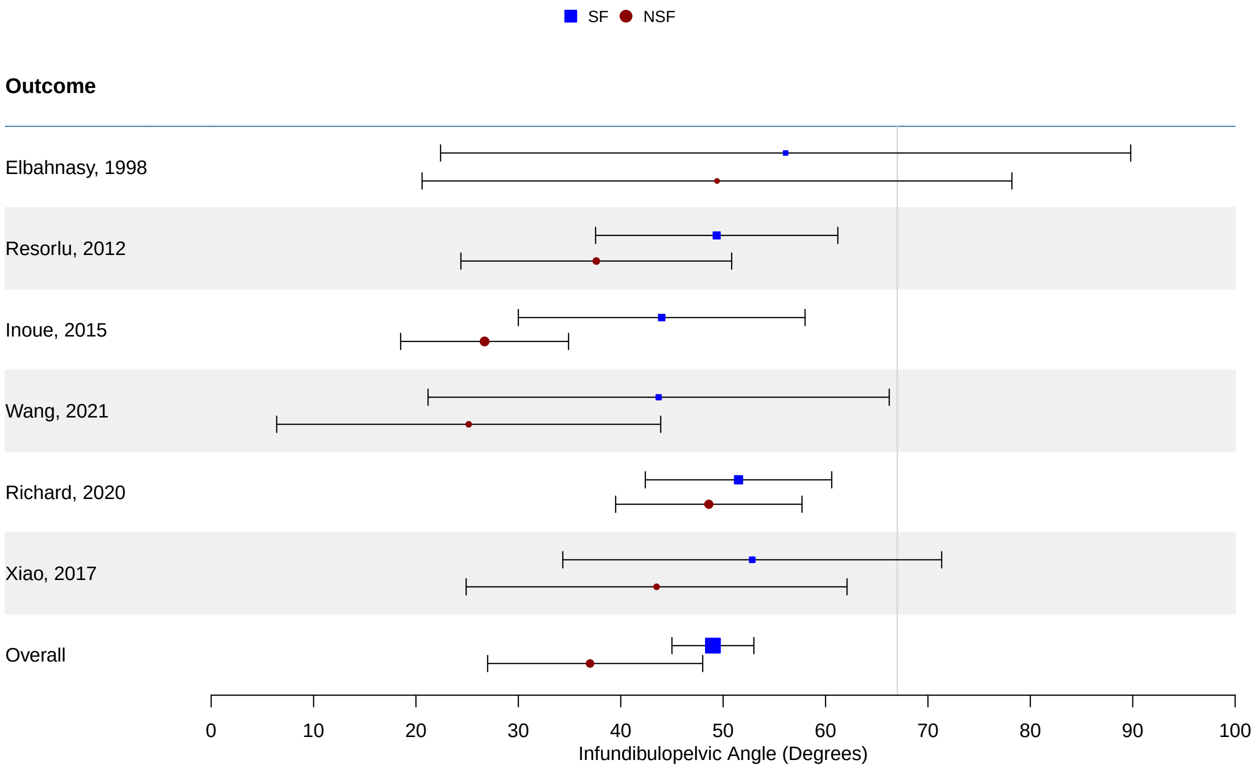
SF
NSF
Outcome
Elbahnasy, 1998
Resorlu, 2012
Inoue, 2015
Wang, 2021
Richard, 2020
Xiao, 2017
Overall
0
10
20
30
40
50
60
70
80
90
100
Infundibulopelvic Angle (Degrees)

7.3 Meta-Analysis

7.3.1 Result

```
sf_non_x2_rma <- metacont(data = sf_data_non_x2_reconfig,
                   mean.e = mean_angle_nsf,
                   sd.e = mean_angle_sd_nsf,
                   n.e = n.e,
                   mean.c = mean_angle_sf,
                   sd.c = mean_angle_sd_sf,
                   n.c = n.c,
                   studlab = paste(author, year, sep = ", ")
                   )
sf_non_x2_rma
```

```
## Number of studies: k = 6
## Number of observations: o = 838
##
##                              MD              95%-CI     z  p-value
## Common effect model   -5.2343 [ -6.8931; -3.5756] -6.18 < 0.0001
## Random effects model -11.1552 [-17.0284; -5.2819] -3.72   0.0002
##
## Quantifying heterogeneity:
##  tau^2 = 35.5408 [6.5049; 198.1079]; tau = 5.9616 [2.5505; 14.0751]
##  I^2 = 84.5% [67.9%; 92.5%]; H = 2.54 [1.77; 3.65]
##
## Test of heterogeneity:
##      Q d.f.  p-value
##  32.22    5 < 0.0001
##
## Details on meta-analytical method:
## - Inverse variance method
## - Restricted maximum-likelihood estimator for tau^2
## - Q-Profile method for confidence interval of tau^2 and tau
```

7.3.2 Forest plot

```
forest(sf_non_x2_rma,
       sortvar = TE)
```

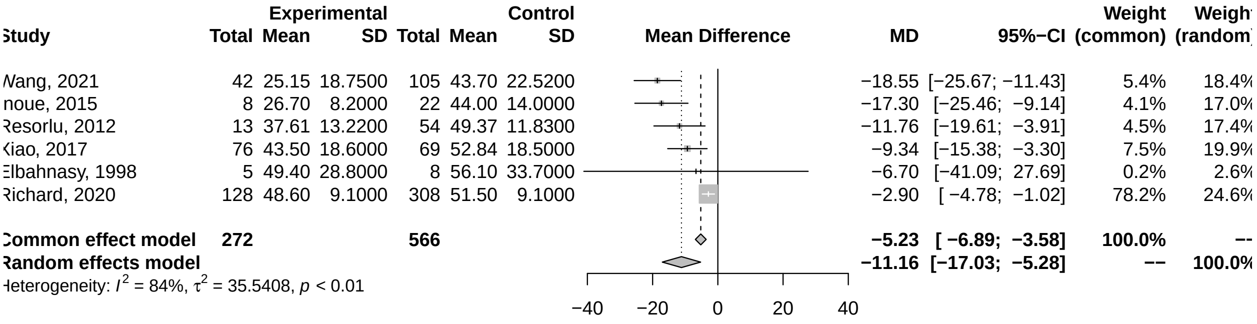

7.3.3 Trim and Fill

```r
trimfill(sf_non_x2_rma)
```

```
## Number of studies: k = 9 (with 3 added studies)
## Number of observations: o = 1082
##
##                           MD              95%-CI     z p-value
## Random effects model -4.2422 [-12.0720; 3.5876] -1.06  0.2883
##
## Quantifying heterogeneity:
##  tau^2 = 119.8904 [42.4424; 447.9282]; tau = 10.9494 [6.5148; 21.1643]
##  I^2 = 88.4% [80.2%; 93.2%]; H = 2.94 [2.25; 3.84]
##
## Test of heterogeneity:
##      Q d.f.  p-value
##  69.09    8 < 0.0001
##
## Details on meta-analytical method:
## - Inverse variance method
## - Restricted maximum-likelihood estimator for tau^2
## - Q-Profile method for confidence interval of tau^2 and tau
## - Trim-and-fill method to adjust for funnel plot asymmetry (L-estimator)
```

```r
funnel(trimfill(sf_non_x2_rma))
```

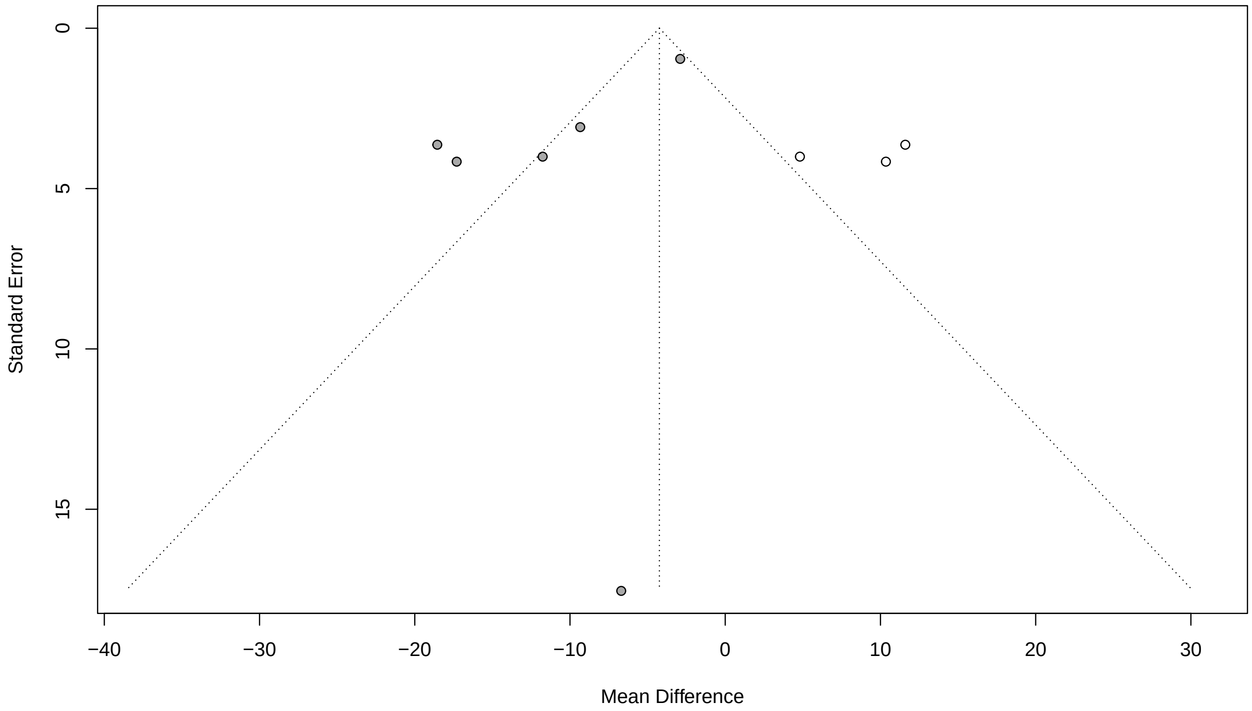


```r
forest(trimfill(sf_non_x2_rma),
       sortvar = TE)
```

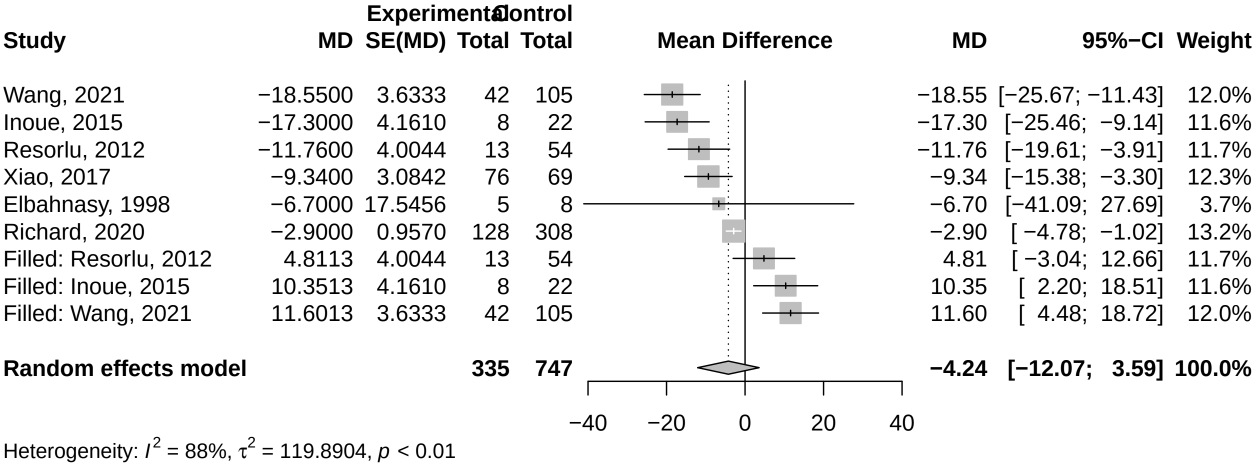

7.3.4 Baujat

```
baujat(sf_non_x2_rma)
```

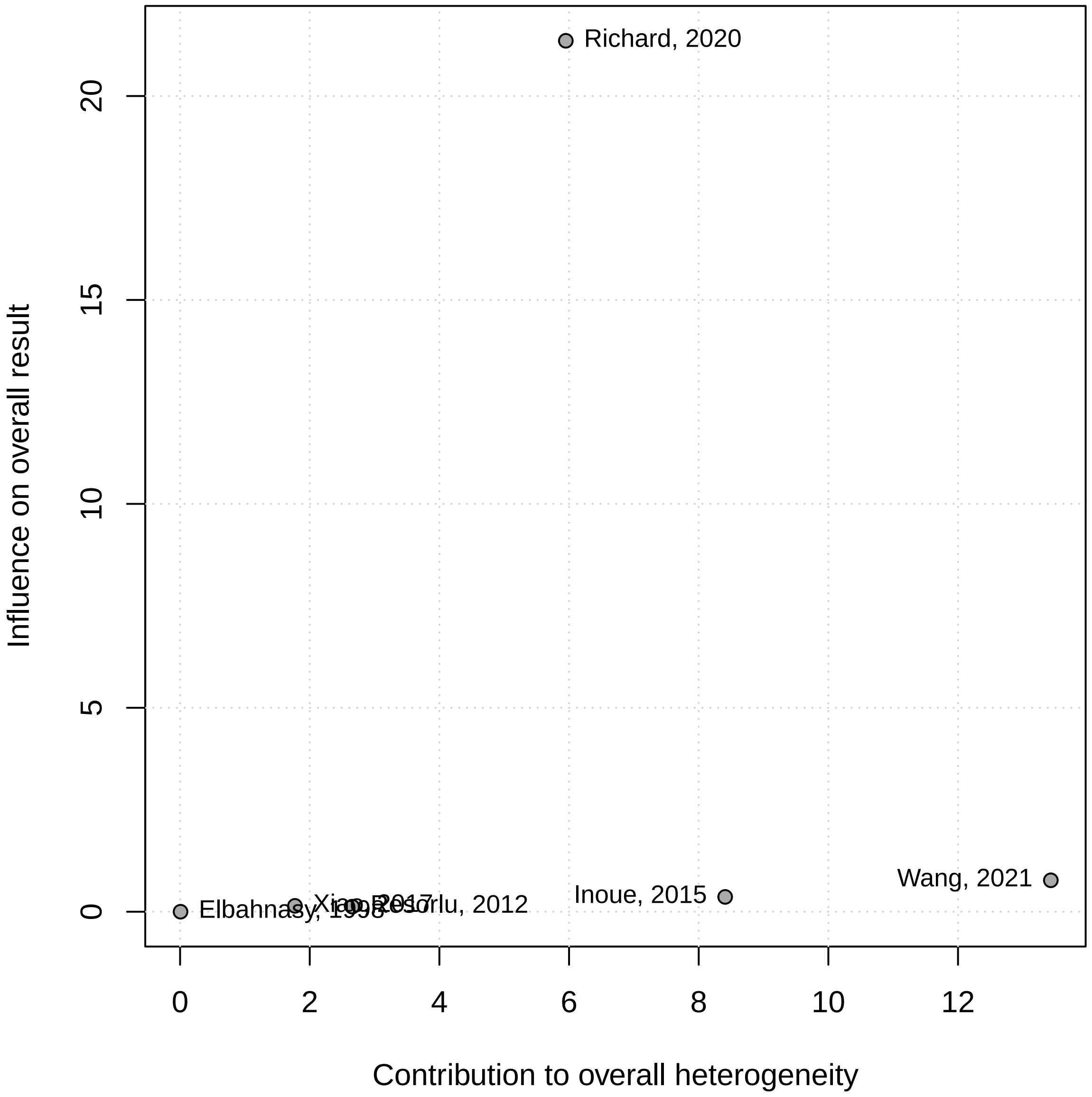
Richard, 2020
Wang, 2021
Inoue, 2015
Elbahnasy, 1998
Xiao, 2017
Resorlu, 2012
Influence on overall result
Contribution to overall heterogeneity
0
5
10
15
20
0
2
4
6
8
10
12

8 SF cut-off for No fragments

8.1 Sort Data

```
sf_data_no_frags <- sf_data %>%
  filter(author %in% c("Sari", "Elbahnasy", "Jessen", "Inoue", "Wang", "Richard", "Kilicarslan"))



sf_data_no_frags_nsf <- sf_data_non_x2 %>% filter(outcome !="SF") %>% mutate("mean_angle_nsf" = mean_ang
                                                       "mean_angle_sd_nsf" = mean_angle_sd,
                                                       "n.e" = number_in_study)
sf_data_no_frags_sf <- sf_data_non_x2 %>% filter(outcome !="NSF") %>% mutate("mean_angle_sf" = mean_angl
                                                       "mean_angle_sd_sf" = mean_angle_sd,
                                                       "n.c" = number_in_study)

sf_data_no_frags_reconfig <- cbind(sf_data_no_frags_nsf, sf_data_no_frags_sf)
sf_data_no_frags_reconfig$mean_angle_nsf<-as.numeric(sf_data_no_frags_reconfig$mean_angle_nsf)
sf_data_no_frags_reconfig$mean_angle_sd_nsf<-as.numeric(sf_data_no_frags_reconfig$mean_angle_sd_nsf)
sf_data_no_frags_reconfig$mean_angle_sf<-as.numeric(sf_data_no_frags_reconfig$mean_angle_sf)
sf_data_no_frags_reconfig$mean_angle_sd_sf<-as.numeric(sf_data_no_frags_reconfig$mean_angle_sd_sf)
sf_data_no_frags_reconfig$n.e<-as.numeric(sf_data_no_frags_reconfig$n.e)
sf_data_no_frags_reconfig$n.c<-as.numeric(sf_data_no_frags_reconfig$n.c)
```

8.1.1 Overall Number of patients

```
sum(sf_data_no_frags$number_in_study)
```

```
## [1] 869
```

8.1.2 SF - Number of patients

```
sum(sf_data_no_frags_sf$number_in_study)
```

```
## [1] 566
```

8.1.3 NSF - Number of patients

```
sum(sf_data_no_frags_nsf$number_in_study)
```

```
## [1] 272
```

8.2 Summary forestplot for SF

Above 67o all patients are stone free Below 30o no patients are stone free

These cut-offs fit with Kilicarslan (>70) and Geavlete (<30) respectively (see below)

By excluding Sari et al., we move the angle at which all patients become SF to ~60 Need to check how SF defined - is definition accounting for difference in SFR between Sari and remainder?

```
sf_data_no_frags <- sf_data_no_frags %>% mutate("lower" = mean_angle - mean_angle_sd,
                              "upper" = mean_angle + mean_angle_sd,
                              "angle" = mean_angle)

sf_data_no_frags$lower <- as.numeric(sf_data_no_frags$lower)
sf_data_no_frags$upper <- as.numeric(sf_data_no_frags$upper)
sf_data_no_frags$angle <- as.numeric(sf_data_no_frags$angle)
sf_data_no_frags$outcome <- as.factor(sf_data_no_frags$outcome)

sf_data_no_frags <- sf_data_no_frags %>% mutate(stud_lab = paste(author,
                                              year,
                                              sep = ", "))

sf_data_sf_no_frags <- metamean(n = number_in_study,
                   mean = mean_angle,
                   sd = mean_angle_sd,
                   studlab = author,
                   data = subset(sf_data_no_frags,
                                 outcome == "SF"),
                   sm = "MRAW",
                   fixed = FALSE,
                   random = TRUE,
                   method.tau = "REML",
                   hakn = TRUE,
                   title = "SF Mean")
sf_data_nsf_no_frags <-metamean(n = number_in_study,
                   mean = mean_angle,
                   sd = mean_angle_sd,
                   studlab = author,
                   data = subset(sf_data_no_frags,
                                 outcome == "NSF"),
                   sm = "MRAW",
                   fixed = FALSE,
                   random = TRUE,
                   method.tau = "REML",
                   hakn = TRUE,
                   title = "NSF Mean")

sf_data_no_frags_minimised <- sf_data_no_frags %>% subset(select = c(stud_lab,
                                                   outcome,
                                                   angle,
                                                   lower,
                                                   upper)) %>% rbind(c(
                                                     "Overall",
                                                     "SF",
                                                     round(sf_data_sf_no_frags$TE.random),
                                                     round(sf_data_sf_no_frags$lower.random),
```

```
                                                     round(sf_data_sf_no_frags$upper.random)
                                                   )) %>% rbind(c(
                                                     "Overall",
                                                     "NSF",
                                                     round(sf_data_nsf_no_frags$TE.random),
                                                     round(sf_data_nsf_no_frags$lower.random),
                                                     round(sf_data_nsf_no_frags$upper.random)
                                                   ))

sf_data_no_frags_minimised$outcome <- factor(sf_data_no_frags_minimised$outcome,
                                    levels = c("SF",
                                               "NSF"))
sf_data_no_frags_minimised$angle<- as.numeric(sf_data_no_frags_minimised$angle)
sf_data_no_frags_minimised$lower<- as.numeric(sf_data_no_frags_minimised$lower)
sf_data_no_frags_minimised$upper<- as.numeric(sf_data_no_frags_minimised$upper)

sf_data_no_frags_minimised %>% group_by(outcome) %>% forestplot(
  mean = angle,
  lower = lower,
  upper = upper,
  labeltext = stud_lab,
  fn.ci_norm = c(fpDrawNormalCI, fpDrawCircleCI),
  zero = 67,
  vertices = TRUE,
  cex = 2,
  lineheight = "auto",
  xlab = "Infundibulopelvic Angle (Degrees)",
  xticks = c(0, 10, 20, 30, 40, 50, 60, 70, 80, 90, 100),
) %>% fp_set_style(
  box = c("blue", "darkred"),
  line = "black",
  txt_gp = fpTxtGp(
    ticks = gpar(fontfamily = "", cex = 1),
    xlab  = gpar(fontfamily = "", cex = 1)
  )
) %>% fp_add_lines("steelblue") %>%
  fp_add_header("Outcome") %>% fp_set_zebra_style("#EFEFEFEF")
```

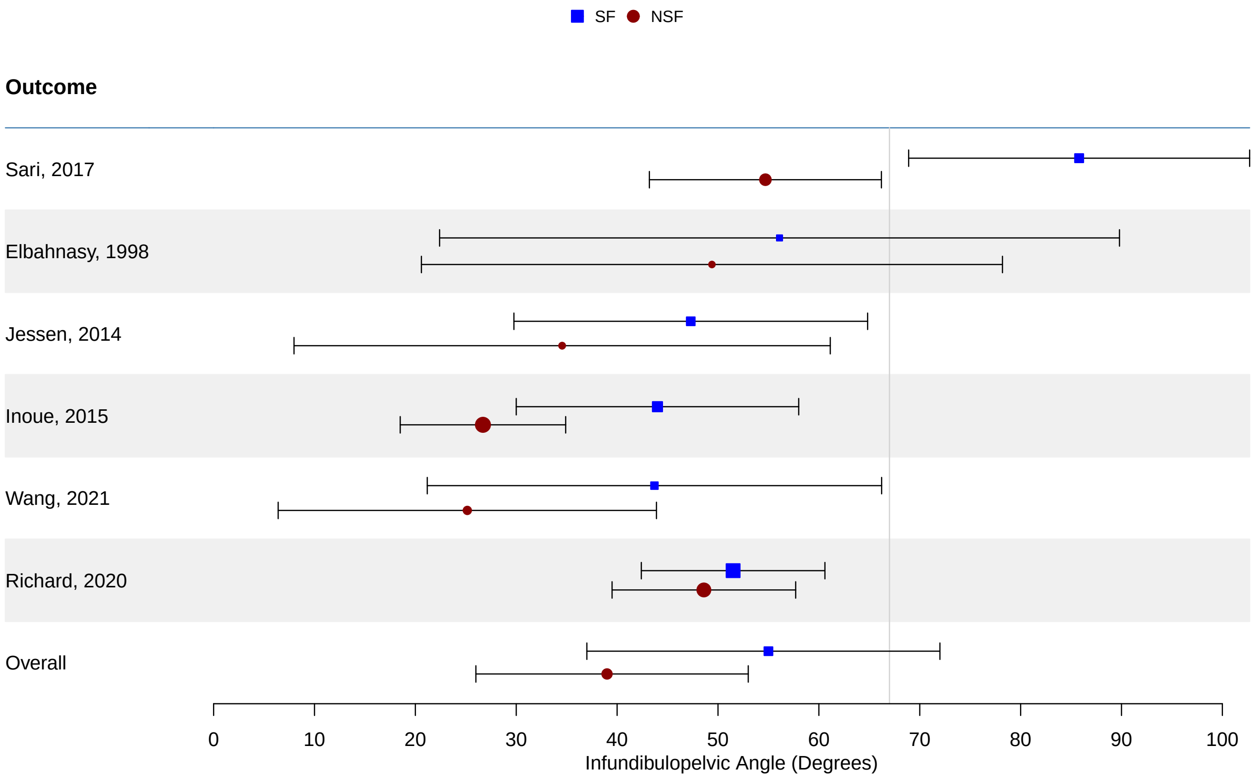
SF
NSF
Outcome
Sari, 2017
Elbahnasy, 1998
Jessen, 2014
Inoue, 2015
Wang, 2021
Richard, 2020
Overall
0
10
20
30
40
50
60
70
80
90
100
Infundibulopelvic Angle (Degrees)

8.3 Meta-Analysis

8.3.1 Result

```
sf_no_frags_rma <- metacont(data = sf_data_no_frags_reconfig,
                   mean.e = mean_angle_nsf,
                   sd.e = mean_angle_sd_nsf,
                   n.e = n.e,
                   mean.c = mean_angle_sf,
                   sd.c = mean_angle_sd_sf,
                   n.c = n.c,
                   studlab = paste(author, year, sep = ", ")
                   )
sf_no_frags_rma
```

```
## Number of studies: k = 6
## Number of observations: o = 838
##
##                              MD               95%-CI     z  p-value
## Common effect model   -5.2343 [ -6.8931; -3.5756] -6.18 < 0.0001
## Random effects model -11.1552 [-17.0284; -5.2819] -3.72   0.0002
##
## Quantifying heterogeneity:
##  tau^2 = 35.5408 [6.5049; 198.1079]; tau = 5.9616 [2.5505; 14.0751]
##  I^2 = 84.5% [67.9%; 92.5%]; H = 2.54 [1.77; 3.65]
##
## Test of heterogeneity:
##      Q d.f.  p-value
##  32.22    5 < 0.0001
##
## Details on meta-analytical method:
## - Inverse variance method
## - Restricted maximum-likelihood estimator for tau^2
## - Q-Profile method for confidence interval of tau^2 and tau
```

8.3.2 Forest plot

```r
forest(sf_no_frags_rma,
       sortvar = TE)
```

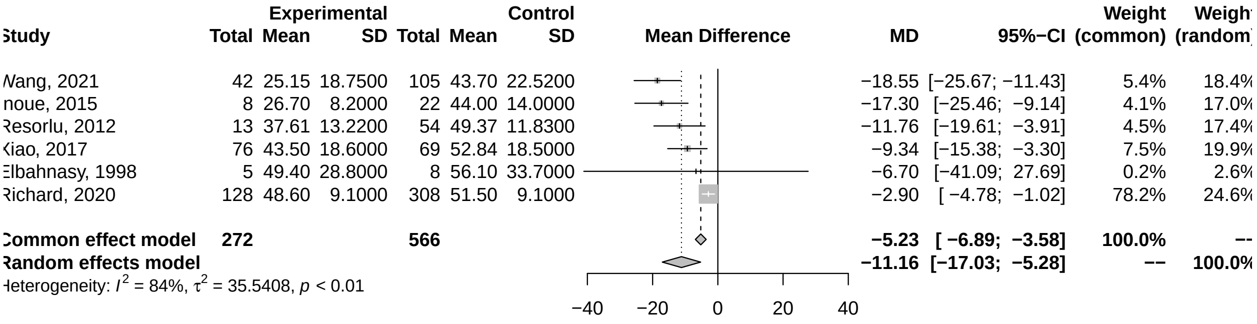

8.3.3 Trim and Fill

```r
trimfill(sf_no_frags_rma)
```

```
## Number of studies: k = 9 (with 3 added studies)
## Number of observations: o = 1082
##
##                              MD              95%-CI     z p-value
## Random effects model -4.2422 [-12.0720; 3.5876] -1.06  0.2883
##
## Quantifying heterogeneity:
##  tau^2 = 119.8904 [42.4424; 447.9282]; tau = 10.9494 [6.5148; 21.1643]
##  I^2 = 88.4% [80.2%; 93.2%]; H = 2.94 [2.25; 3.84]
##
## Test of heterogeneity:
##      Q d.f.  p-value
##  69.09    8 < 0.0001
##
## Details on meta-analytical method:
## - Inverse variance method
## - Restricted maximum-likelihood estimator for tau^2
## - Q-Profile method for confidence interval of tau^2 and tau
## - Trim-and-fill method to adjust for funnel plot asymmetry (L-estimator)
```

```r
funnel(trimfill(sf_no_frags_rma))
```

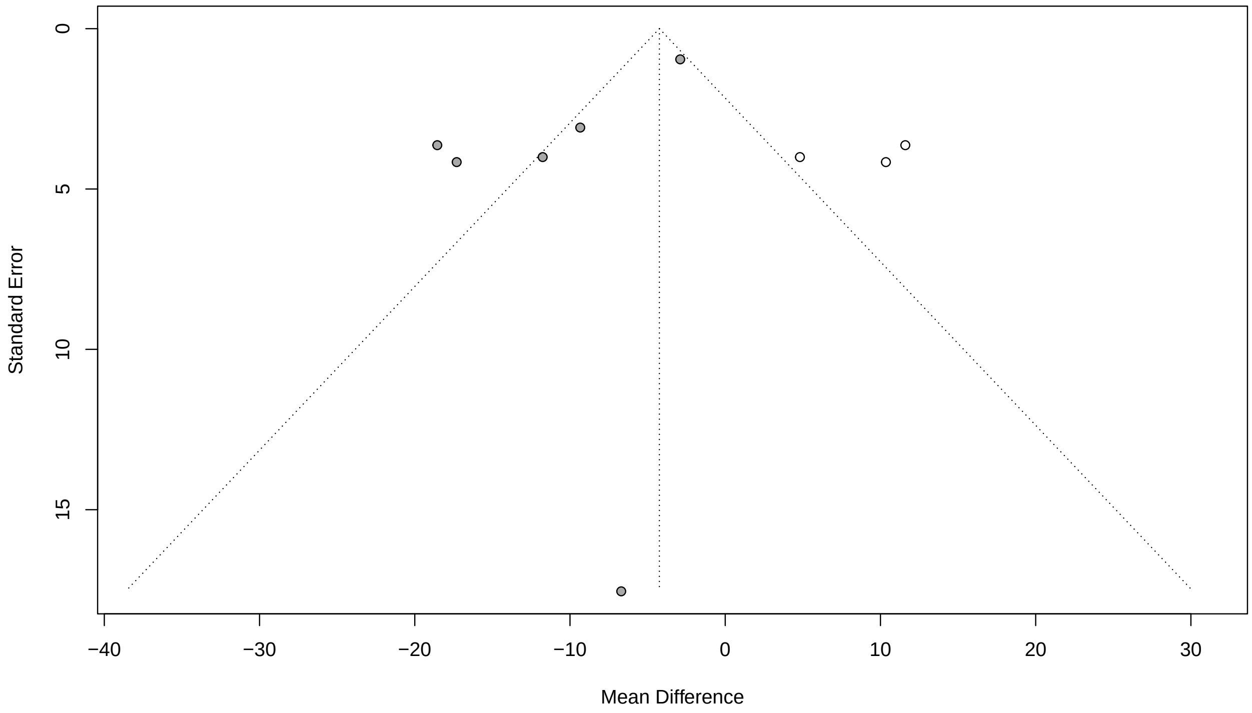


```r
forest(trimfill(sf_no_frags_rma),
       sortvar = TE)
```

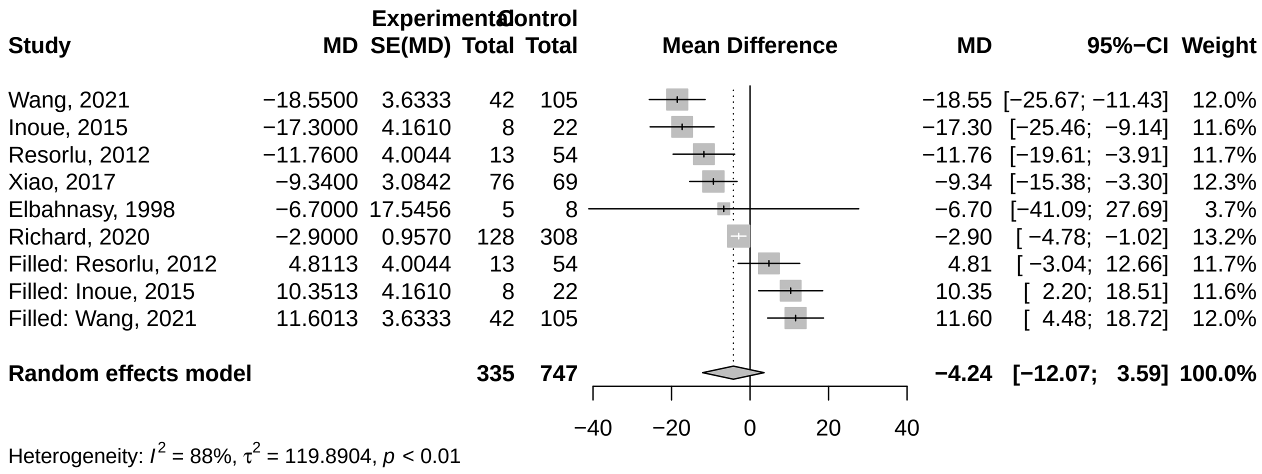

8.3.4 Baujat

```
baujat(sf_no_frags_rma)
```

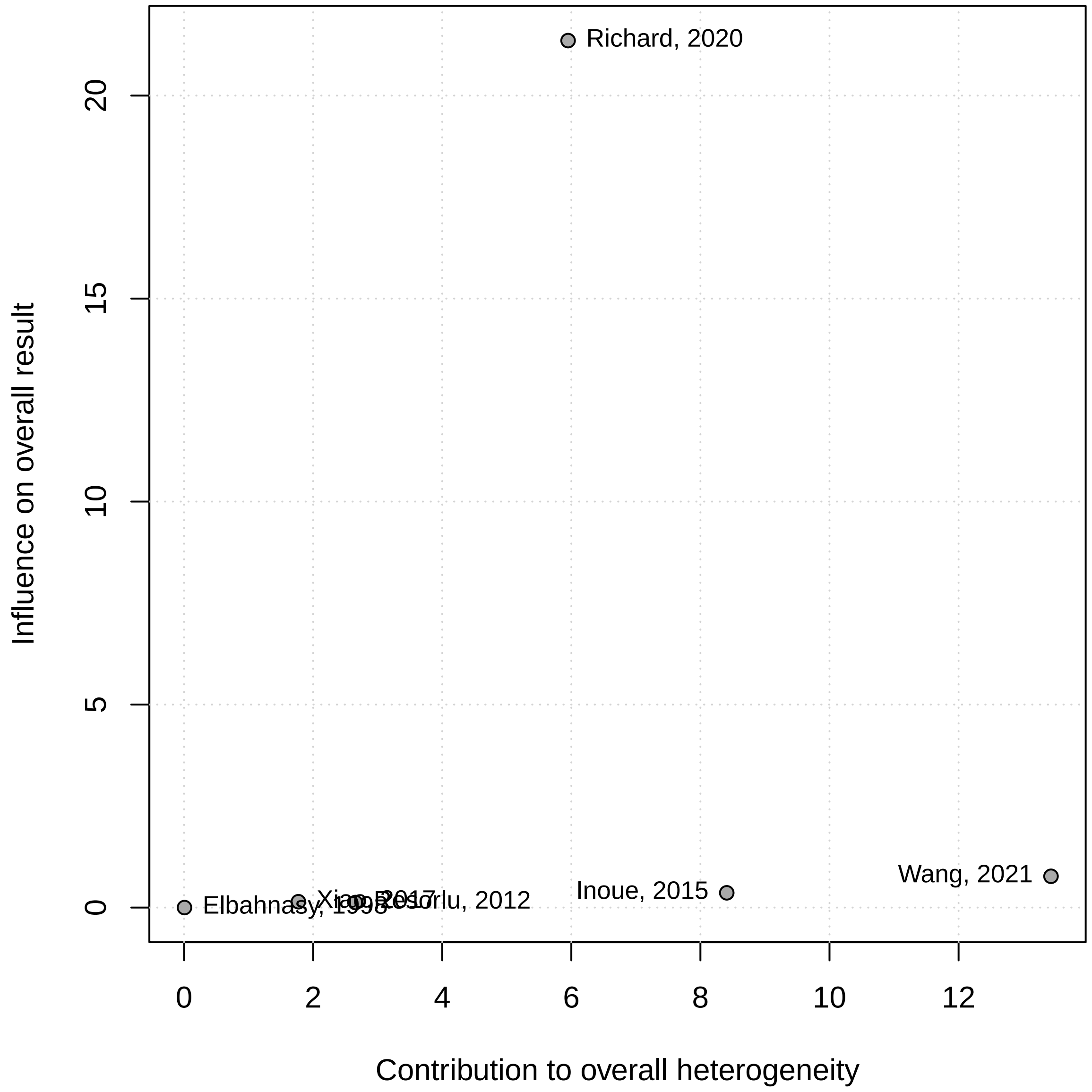
Richard, 2020
Wang, 2021
Inoue, 2015
Xiao, 2017
Resorlu, 2012
Elbahnasy, 1998
Influence on overall result
Contribution to overall heterogeneity
0
5
10
15
20
0
2
4
6
8
10
12

9 SF cut-off for <4mm fragments (any accepted fragment size)

9.1 Sort Data

```
sf_data_frags <- sf_data %>%
  filter(!author %in% c("Sari", "Elbahnasy", "Jessen", "Inoue", "Wang", "Richard", "Kilicarslan"))



sf_data_frags_nsf <- sf_data_frags %>% filter(outcome !="SF") %>% mutate("mean_angle_nsf" = mean_angle,
                                                     "mean_angle_sd_nsf" = mean_angle_sd,
                                                     "n.e" = number_in_study)
sf_data_frags_sf <- sf_data_frags %>% filter(outcome !="NSF") %>% mutate("mean_angle_sf" = mean_angle,
                                                     "mean_angle_sd_sf" = mean_angle_sd,
                                                     "n.c" = number_in_study)

sf_data_frags_reconfig <- cbind(sf_data_frags_nsf, sf_data_frags_sf)
sf_data_frags_reconfig$mean_angle_nsf<-as.numeric(sf_data_frags_reconfig$mean_angle_nsf)
sf_data_frags_reconfig$mean_angle_sd_nsf<-as.numeric(sf_data_frags_reconfig$mean_angle_sd_nsf)
sf_data_frags_reconfig$mean_angle_sf<-as.numeric(sf_data_frags_reconfig$mean_angle_sf)
sf_data_frags_reconfig$mean_angle_sd_sf<-as.numeric(sf_data_frags_reconfig$mean_angle_sd_sf)
sf_data_frags_reconfig$n.e<-as.numeric(sf_data_frags_reconfig$n.e)
sf_data_frags_reconfig$n.c<-as.numeric(sf_data_frags_reconfig$n.c)
```

9.1.1 Overall Number of patients

```
sum(sf_data_frags$number_in_study)
```

```
## [1] 488
```

9.1.2 SF - Number of patients

```
sum(sf_data_frags_sf$number_in_study)
```

```
## [1] 330
```

9.1.3 NSF - Number of patients

```
sum(sf_data_frags_nsf$number_in_study)
```

```
## [1] 158
```

9.2 Summary forestplot for SF

Above 67o all patients are stone free Below 30o no patients are stone free

These cut-offs fit with Kilicarslan (>70) and Geavlete (<30) respectively (see below)

By excluding Sari et al., we move the angle at which all patients become SF to ~60 Need to check how SF defined - is definition accounting for difference in SFR between Sari and remainder?

```
sf_data_frags <- sf_data_frags %>% mutate("lower" = mean_angle - mean_angle_sd,
                              "upper" = mean_angle + mean_angle_sd,
                              "angle" = mean_angle)

sf_data_frags$lower <- as.numeric(sf_data_frags$lower)
sf_data_frags$upper <- as.numeric(sf_data_frags$upper)
sf_data_frags$angle <- as.numeric(sf_data_frags$angle)
sf_data_frags$outcome <- as.factor(sf_data_frags$outcome)

sf_data_frags <- sf_data_frags %>% mutate(stud_lab = paste(author,
                                               year,
                                               sep = ", "))

sf_data_sf_frags <- metamean(n = number_in_study,
                   mean = mean_angle,
                   sd = mean_angle_sd,
                   studlab = author,
                   data = subset(sf_data_frags,
                                 outcome == "SF"),
                   sm = "MRAW",
                   fixed = FALSE,
                   random = TRUE,
                   method.tau = "REML",
                   hakn = TRUE,
                   title = "SF Mean")
sf_data_nsf_frags <-metamean(n = number_in_study,
                   mean = mean_angle,
                   sd = mean_angle_sd,
                   studlab = author,
                   data = subset(sf_data_frags,
                                 outcome == "NSF"),
                   sm = "MRAW",
                   fixed = FALSE,
                   random = TRUE,
                   method.tau = "REML",
                   hakn = TRUE,
                   title = "NSF Mean")

sf_data_frags_minimised <- sf_data_frags %>% subset(select = c(stud_lab,
                                                   outcome,
                                                   angle,
                                                   lower,
                                                   upper)) %>% rbind(c(
                                                     "Overall",
                                                     "SF",
                                                     round(sf_data_sf_frags$TE.random),
                                                     round(sf_data_sf_frags$lower.random),
```

```r
                                                    round(sf_data_sf_frags$upper.random)
                                                  )) %>% rbind(c(
                                                    "Overall",
                                                    "NSF",
                                                    round(sf_data_nsf_frags$TE.random),
                                                    round(sf_data_nsf_frags$lower.random),
                                                    round(sf_data_nsf_frags$upper.random)
                                                  ))

sf_data_frags_minimised$outcome <- factor(sf_data_frags_minimised$outcome,
                                    levels = c("SF",
                                               "NSF"))
sf_data_frags_minimised$angle<- as.numeric(sf_data_frags_minimised$angle)
sf_data_frags_minimised$lower<- as.numeric(sf_data_frags_minimised$lower)
sf_data_frags_minimised$upper<- as.numeric(sf_data_frags_minimised$upper)

sf_data_frags_minimised %>% group_by(outcome) %>% forestplot(
  mean = angle,
  lower = lower,
  upper = upper,
  labeltext = stud_lab,
  fn.ci_norm = c(fpDrawNormalCI, fpDrawCircleCI),
  zero = 67,
  vertices = TRUE,
  cex = 2,
  lineheight = "auto",
  xlab = "Infundibulopelvic Angle (Degrees)",
  xticks = c(0, 10, 20, 30, 40, 50, 60, 70, 80, 90, 100),
) %>% fp_set_style(
  box = c("blue", "darkred"),
  line = "black",
  txt_gp = fpTxtGp(
    ticks = gpar(fontfamily = "", cex = 1),
    xlab  = gpar(fontfamily = "", cex = 1)
  )
) %>% fp_add_lines("steelblue") %>%
  fp_add_header("Outcome") %>% fp_set_zebra_style("#EFEFEFEF")
```

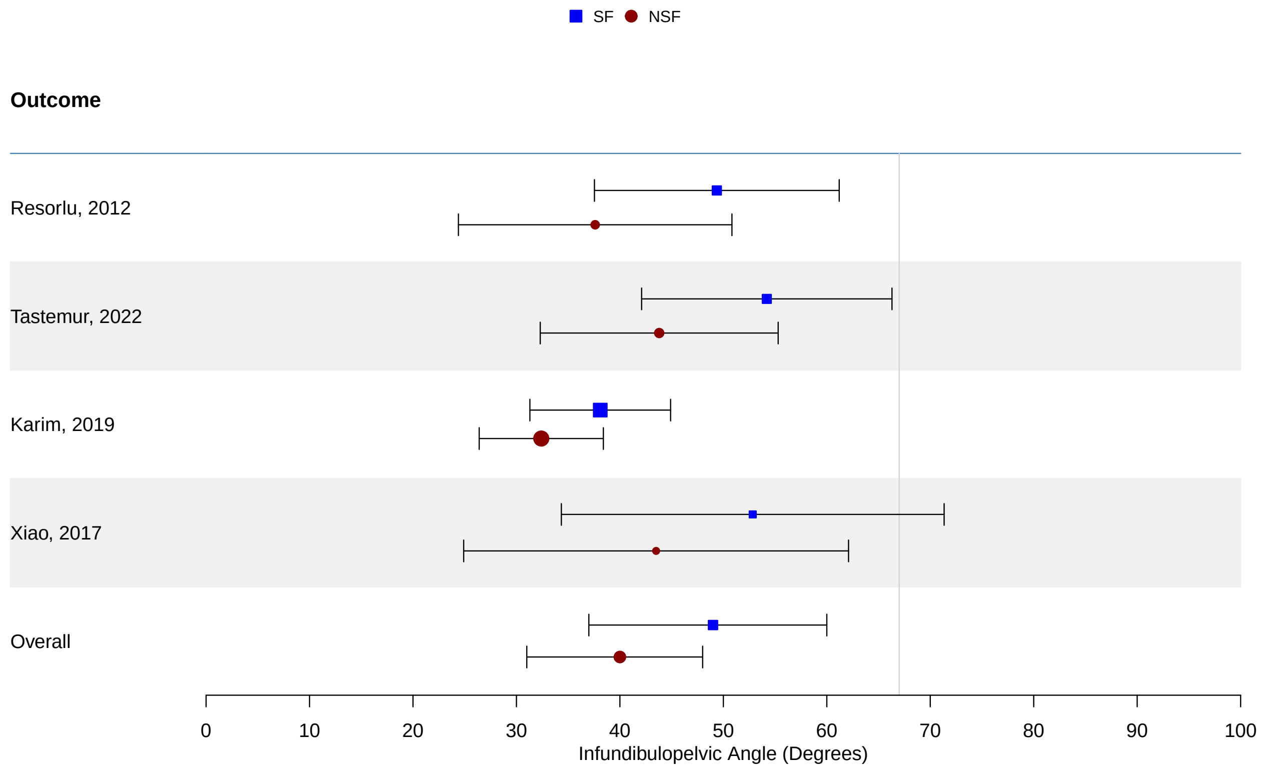
SF
NSF
Outcome
Resorlu, 2012
Tastemur, 2022
Karim, 2019
Xiao, 2017
Overall
0
10
20
30
40
50
60
70
80
90
100
Infundibulopelvic Angle (Degrees)

9.3 Meta-Analysis

9.3.1 Result

```
sf_frags_rma <- metacont(data = sf_data_frags_reconfig,
                    mean.e = mean_angle_nsf,
                    sd.e = mean_angle_sd_nsf,
                    n.e = n.e,
                    mean.c = mean_angle_sf,
                    sd.c = mean_angle_sd_sf,
                    n.c = n.c,
                    studlab = paste(author, year, sep = ", ")
                    )
sf_frags_rma
```

```
## Number of studies: k = 4
## Number of observations: o = 488
##
##                             MD               95%-CI     z  p-value
## Common effect model  -9.1597 [-11.6719; -6.6476] -7.15 < 0.0001
## Random effects model -9.1438 [-11.7430; -6.5446] -6.89 < 0.0001
##
## Quantifying heterogeneity:
##  tau^2 = 0.3595 [0.0000; 84.7361]; tau = 0.5996 [0.0000; 9.2052]
##  I^2 = 0.0% [0.0%; 84.7%]; H = 1.00 [1.00; 2.56]
##
## Test of heterogeneity:
##     Q d.f. p-value
##  2.72    3  0.4368
##
## Details on meta-analytical method:
## - Inverse variance method
## - Restricted maximum-likelihood estimator for tau^2
## - Q-Profile method for confidence interval of tau^2 and tau
```

9.3.2 Forest plot

```r
forest(sf_frags_rma,
       sortvar = TE)
```

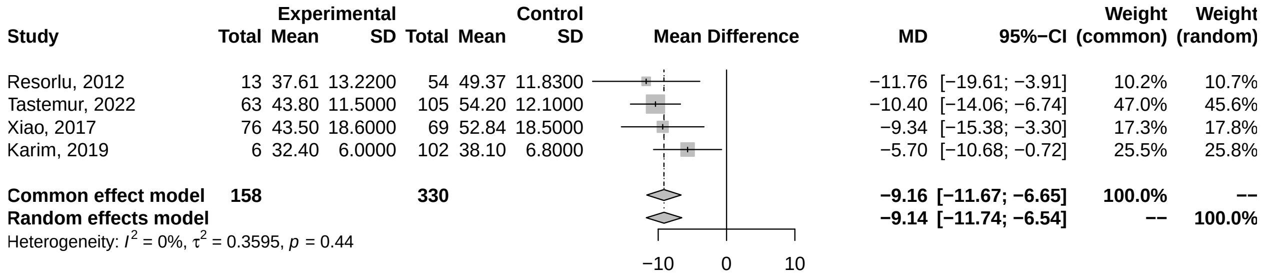

9.3.3 Trim and Fill

```r
trimfill(sf_frags_rma)
```

```
## Number of studies: k = 5 (with 1 added studies)
## Number of observations: o = 555
##
##                          MD              95%-CI     z  p-value
## Random effects model -8.8505 [-11.2776; -6.4235] -7.15 < 0.0001
##
## Quantifying heterogeneity:
##  tau^2 = 0.1570 [0.0000; 48.8105]; tau = 0.3963 [0.0000; 6.9865]
##  I^2 = 0.0% [0.0%; 79.2%]; H = 1.00 [1.00; 2.19]
##
## Test of heterogeneity:
##     Q d.f. p-value
##  3.30    4  0.5094
##
## Details on meta-analytical method:
## - Inverse variance method
## - Restricted maximum-likelihood estimator for tau^2
## - Q-Profile method for confidence interval of tau^2 and tau
## - Trim-and-fill method to adjust for funnel plot asymmetry (L-estimator)
```

```r
funnel(trimfill(sf_frags_rma))
```

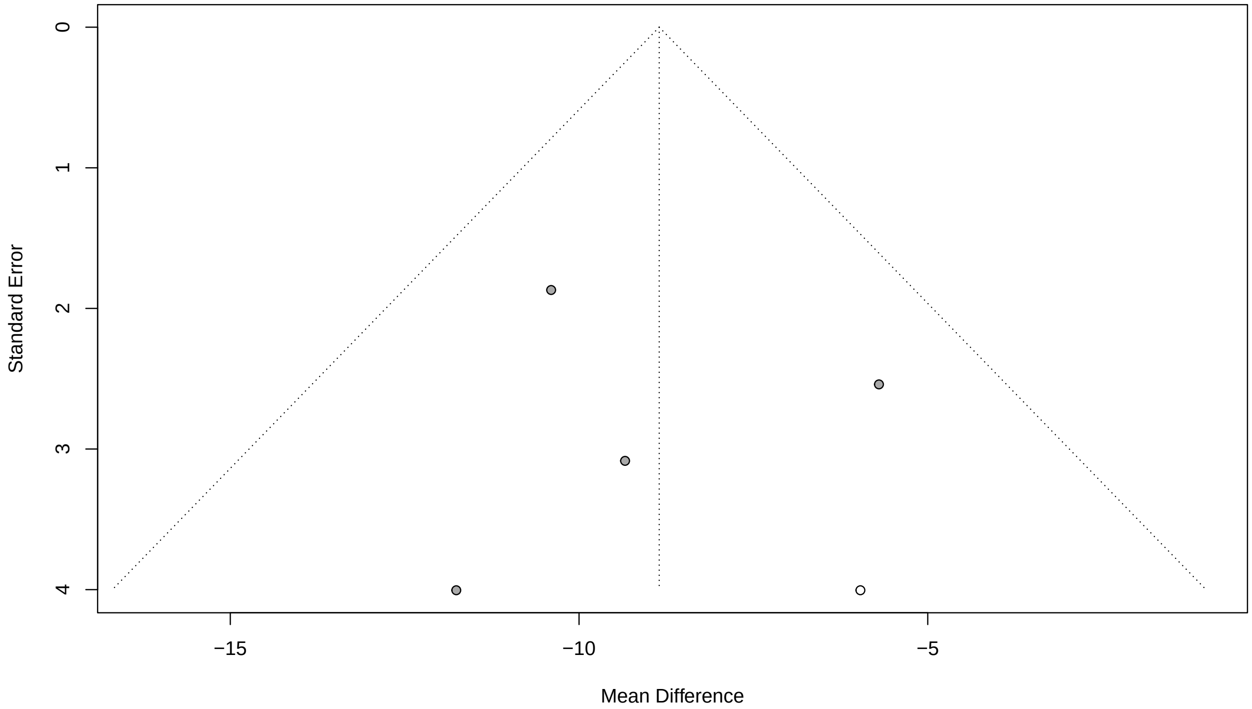


```r
forest(trimfill(sf_frags_rma),
       sortvar = TE)
```

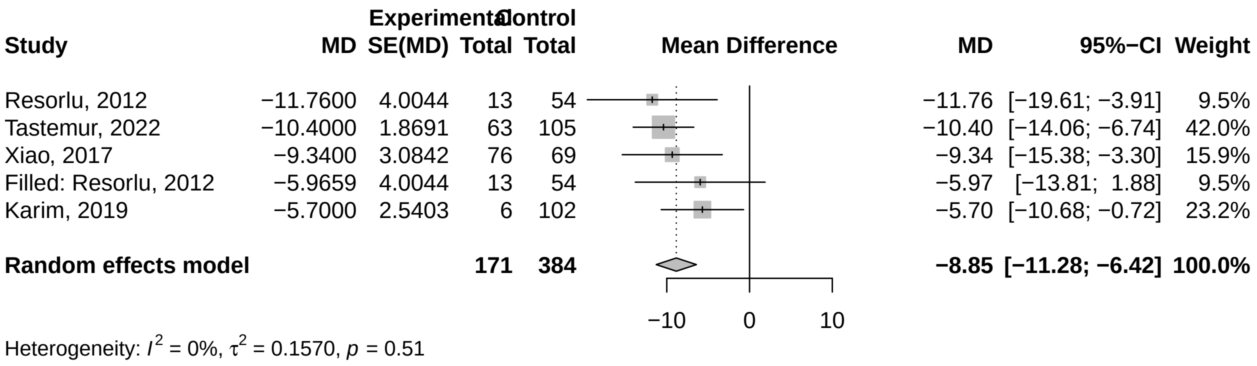

9.3.4 Baujat

```
baujat(sf_frags_rma)
```

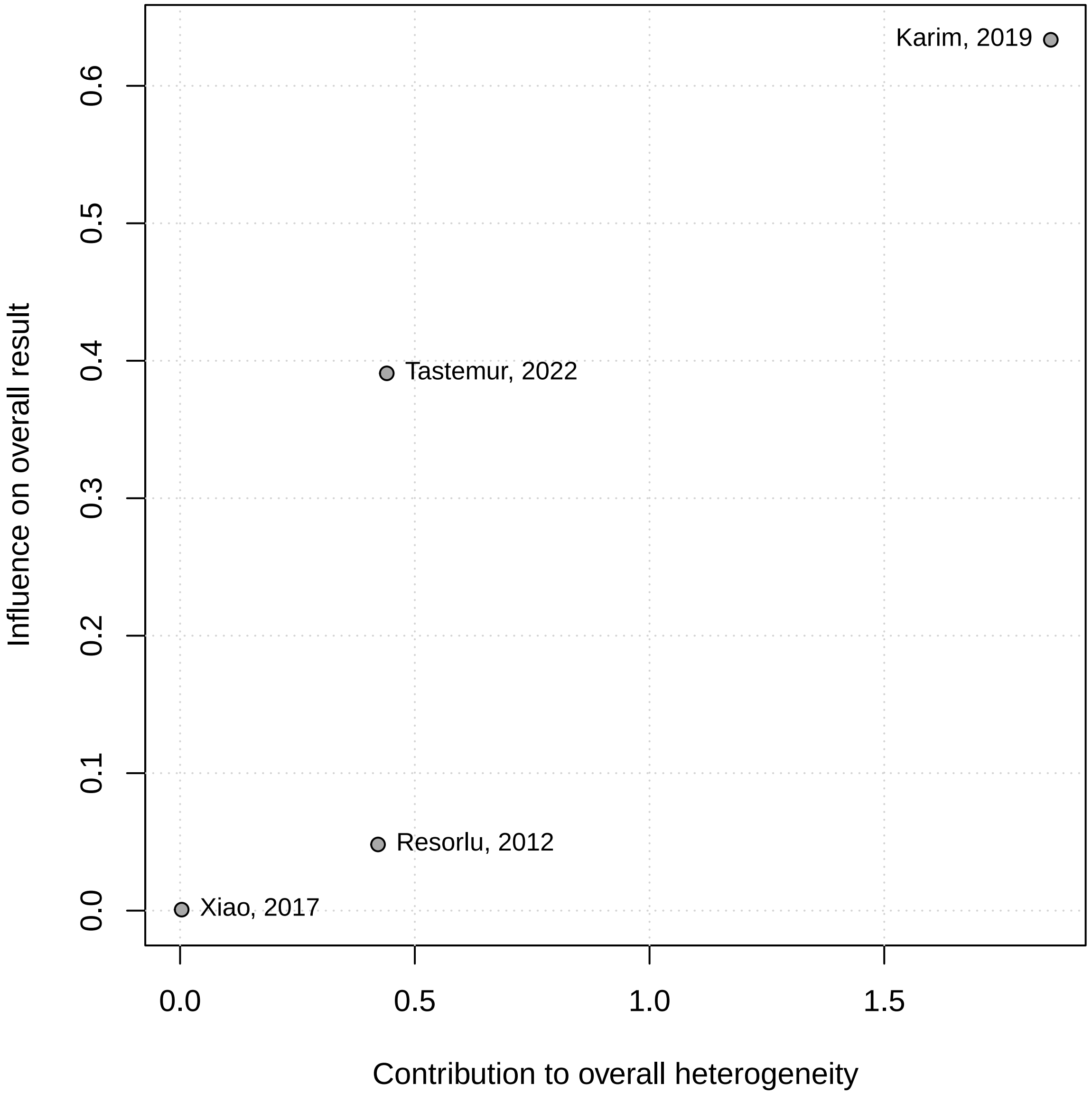

10 SF for LP stones <2cm

10.1 Sort Data

```
sf_data_2cm <- sf_data %>%
  subset(stones_2mm == "no") %>% as_tibble()



sf_data_2cm_nsf <- sf_data_2cm %>% filter(outcome !="SF") %>% mutate("mean_angle_nsf" = mean_angle,
                                                       "mean_angle_sd_nsf" = mean_angle_sd,
                                                       "n.e" = number_in_study)
sf_data_2cm_sf <- sf_data_2cm %>% filter(outcome !="NSF") %>% mutate("mean_angle_sf" = mean_angle,
                                                       "mean_angle_sd_sf" = mean_angle_sd,
                                                       "n.c" = number_in_study)

sf_data_2cm_reconfig <- cbind(sf_data_2cm_nsf, sf_data_2cm_sf)
sf_data_2cm_reconfig$mean_angle_nsf<-as.numeric(sf_data_2cm_reconfig$mean_angle_nsf)
sf_data_2cm_reconfig$mean_angle_sd_nsf<-as.numeric(sf_data_2cm_reconfig$mean_angle_sd_nsf)
sf_data_2cm_reconfig$mean_angle_sf<-as.numeric(sf_data_2cm_reconfig$mean_angle_sf)
sf_data_2cm_reconfig$mean_angle_sd_sf<-as.numeric(sf_data_2cm_reconfig$mean_angle_sd_sf)
sf_data_2cm_reconfig$n.e<-as.numeric(sf_data_2cm_reconfig$n.e)
sf_data_2cm_reconfig$n.c<-as.numeric(sf_data_2cm_reconfig$n.c)
```

10.1.1 Overall Number of patients

```
sum(sf_data_2cm$number_in_study)
```

```
## [1] 232
```

10.1.2 SF - Number of patients

```
sum(sf_data_2cm_sf$number_in_study)
```

```
## [1] 208
```

10.1.3 NSF - Number of patients

```
sum(sf_data_2cm_nsf$number_in_study)
```

```
## [1] 24
```

10.2 Summary forestplot for SF

Above 67o all patients are stone free Below 30o no patients are stone free

These cut-offs fit with Kilicarslan (>70) and Geavlete (<30) respectively (see below)

By excluding Sari et al., we move the angle at which all patients become SF to ~60 Need to check how SF defined - is definition accounting for difference in SFR between Sari and remainder?

```
sf_data_2cm <- sf_data_2cm %>% mutate("lower" = mean_angle - mean_angle_sd,
                              "upper" = mean_angle + mean_angle_sd,
                              "angle" = mean_angle)

sf_data_2cm$lower <- as.numeric(sf_data_2cm$lower)
sf_data_2cm$upper <- as.numeric(sf_data_2cm$upper)
sf_data_2cm$angle <- as.numeric(sf_data_2cm$angle)
sf_data_2cm$outcome <- as.factor(sf_data_2cm$outcome)

sf_data_2cm <- sf_data_2cm %>% mutate(stud_lab = paste(author,
                                                  year,
                                                  sep = ", "))

sf_data_sf_2cm <- metamean(n = number_in_study,
                   mean = mean_angle,
                   sd = mean_angle_sd,
                   studlab = author,
                   data = subset(sf_data_2cm,
                                 outcome == "SF"),
                   sm = "MRAW",
                   fixed = FALSE,
                   random = TRUE,
                   method.tau = "REML",
                   hakn = TRUE,
                   title = "SF Mean")
sf_data_nsf_2cm <-metamean(n = number_in_study,
                   mean = mean_angle,
                   sd = mean_angle_sd,
                   studlab = author,
                   data = subset(sf_data_2cm,
                                 outcome == "NSF"),
                   sm = "MRAW",
                   fixed = FALSE,
                   random = TRUE,
                   method.tau = "REML",
                   hakn = TRUE,
                   title = "NSF Mean")

sf_data_2cm_minimised <- sf_data_2cm %>% subset(select = c(stud_lab,
                                                   outcome,
                                                   angle,
                                                   lower,
                                                   upper)) %>% rbind(c(
                                                     "Overall",
                                                     "SF",
                                                     round(sf_data_sf_2cm$TE.random),
                                                     round(sf_data_sf_2cm$lower.random),
```

```r
                                                    round(sf_data_sf_2cm$upper.random)
                                                  )) %>% rbind(c(
                                                    "Overall",
                                                    "NSF",
                                                    round(sf_data_nsf_2cm$TE.random),
                                                    round(sf_data_nsf_2cm$lower.random),
                                                    round(sf_data_nsf_2cm$upper.random)
                                                  ))

sf_data_2cm_minimised$outcome <- factor(sf_data_2cm_minimised$outcome,
                                    levels = c("SF",
                                               "NSF"))
sf_data_2cm_minimised$angle<- as.numeric(sf_data_2cm_minimised$angle)
sf_data_2cm_minimised$lower<- as.numeric(sf_data_2cm_minimised$lower)
sf_data_2cm_minimised$upper<- as.numeric(sf_data_2cm_minimised$upper)

sf_data_2cm_minimised %>% group_by(outcome) %>% forestplot(
  mean = angle,
  lower = lower,
  upper = upper,
  labeltext = stud_lab,
  fn.ci_norm = c(fpDrawNormalCI, fpDrawCircleCI),
  zero = 67,
  vertices = TRUE,
  cex = 2,
  lineheight = "auto",
  xlab = "Infundibulopelvic Angle (Degrees)",
  xticks = c(0, 10, 20, 30, 40, 50, 60, 70, 80, 90, 100),
) %>% fp_set_style(
  box = c("blue", "darkred"),
  line = "black",
  txt_gp = fpTxtGp(
    ticks = gpar(fontfamily = "", cex = 1),
    xlab  = gpar(fontfamily = "", cex = 1)
  )
) %>% fp_add_lines("steelblue") %>%
  fp_add_header("Outcome") %>% fp_set_zebra_style("#EFEFEFEF")
```

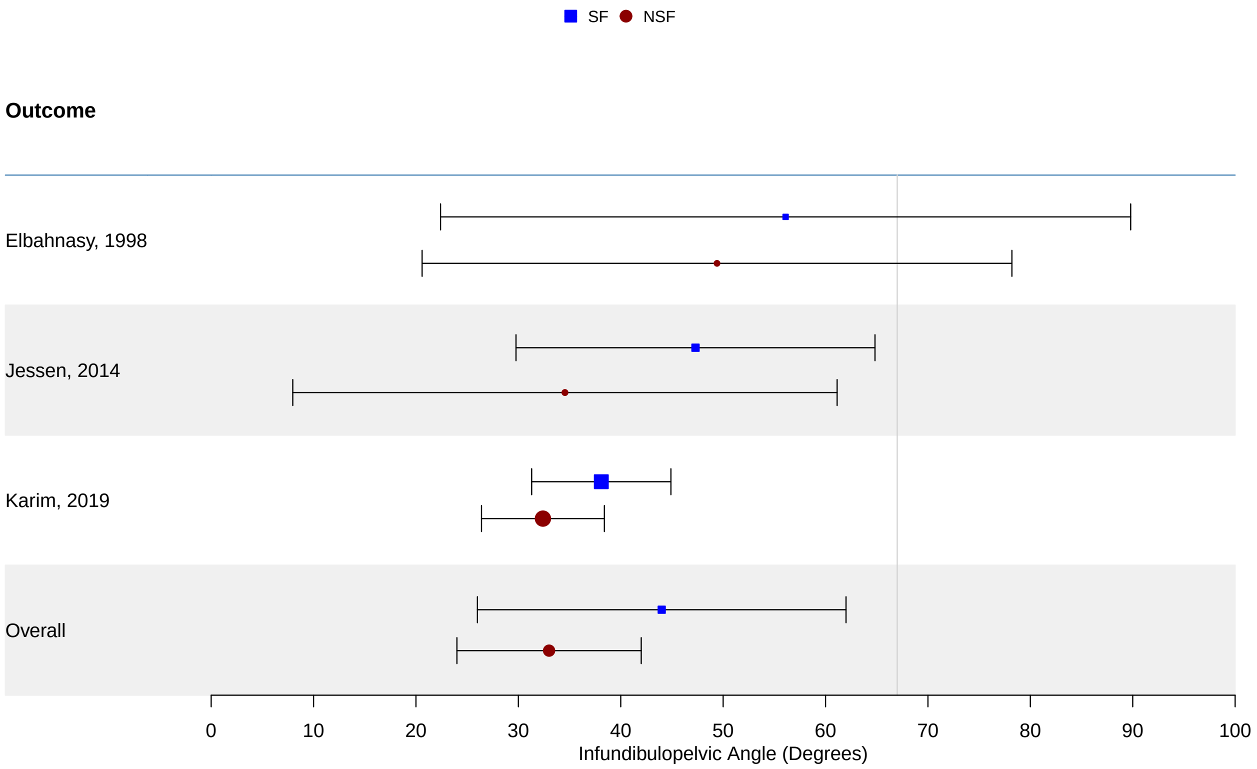
SF
NSF
Outcome
Elbahnasy, 1998
Jessen, 2014
Karim, 2019
Overall
0
10
20
30
40
50
60
70
80
90
100
Infundibulopelvic Angle (Degrees)

10.3 Meta-Analysis

10.3.1 Result

```
sf_2cm_rma <- metacont(data = sf_data_2cm_reconfig,
                   mean.e = mean_angle_nsf,
                   sd.e = mean_angle_sd_nsf,
                   n.e = n.e,
                   mean.c = mean_angle_sf,
                   sd.c = mean_angle_sd_sf,
                   n.c = n.c,
                   studlab = paste(author, year, sep = ", ")
                   )
sf_2cm_rma
```

```
## Number of studies: k = 3
## Number of observations: o = 232
##
##                            MD              95%-CI     z p-value
## Common effect model  -6.4169 [-11.0941; -1.7398] -2.69  0.0072
## Random effects model -6.4169 [-11.0941; -1.7398] -2.69  0.0072
##
## Quantifying heterogeneity:
##  tau^2 = 0 [0.0000; >100.0000]; tau = 0 [0.0000; >10.0000]
##  I^2 = 0.0% [0.0%; 89.6%]; H = 1.00 [1.00; 3.10]
##
## Test of heterogeneity:
##     Q d.f. p-value
##  0.78    2  0.6779
##
## Details on meta-analytical method:
## - Inverse variance method
## - Restricted maximum-likelihood estimator for tau^2
## - Q-Profile method for confidence interval of tau^2 and tau
```

10.3.2 Forest plot

```r
forest(sf_2cm_rma,
       sortvar = TE)
```

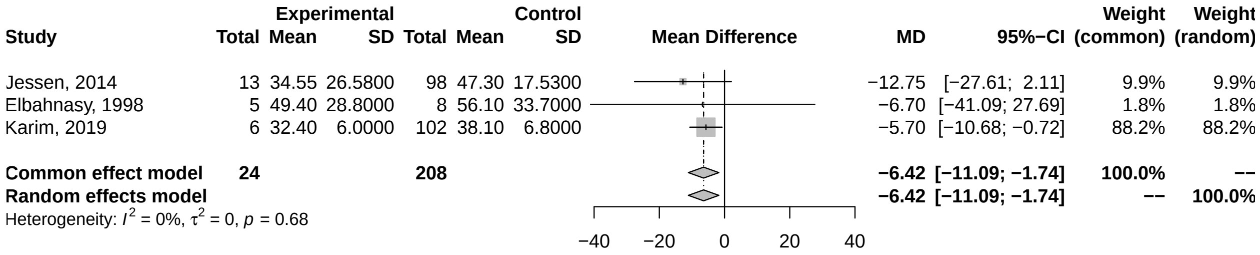

10.3.3 Trim and Fill

```r
trimfill(sf_2cm_rma)
```

```
## Number of studies: k = 5 (with 2 added studies)
## Number of observations: o = 356
##
##                              MD               95%-CI     z p-value
## Random effects model -5.7000 [-10.1243; -1.2757] -2.53  0.0116
##
## Quantifying heterogeneity:
##  tau^2 = 0 [0.0000; >100.0000]; tau = 0 [0.0000; >10.0000]
##  I^2 = 0.0% [0.0%; 79.2%]; H = 1.00 [1.00; 2.19]
##
## Test of heterogeneity:
##     Q d.f. p-value
##  1.74    4  0.7842
##
## Details on meta-analytical method:
## - Inverse variance method
## - Restricted maximum-likelihood estimator for tau^2
## - Q-Profile method for confidence interval of tau^2 and tau
## - Trim-and-fill method to adjust for funnel plot asymmetry (L-estimator)
```

```r
funnel(trimfill(sf_2cm_rma))
```

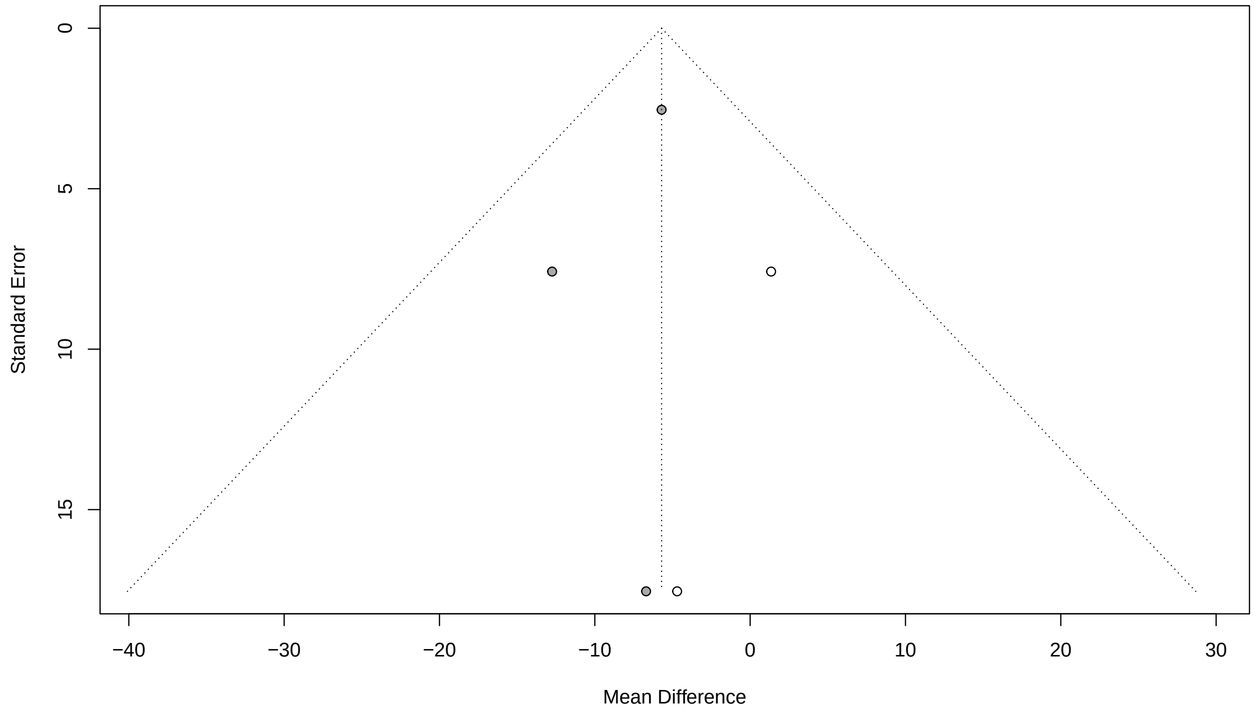


```r
forest(trimfill(sf_2cm_rma),
       sortvar = TE)
```

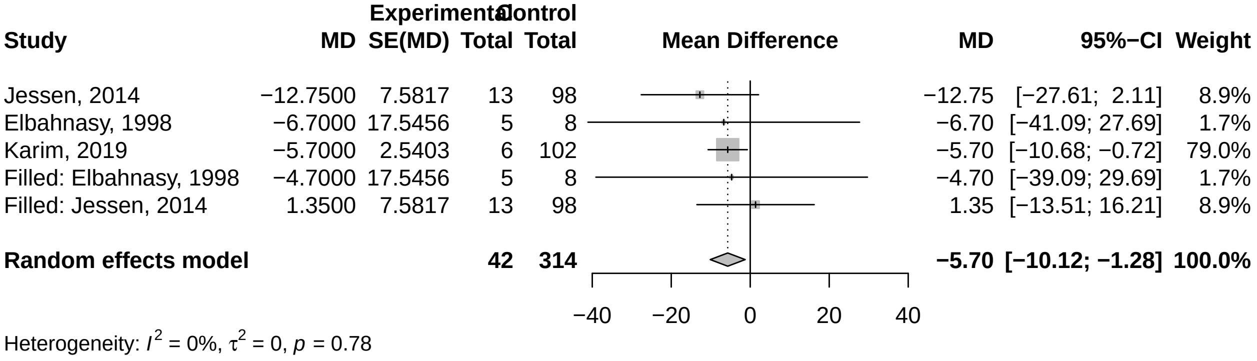

10.3.4 Baujat

```
baujat(sf_2cm_rma)
```

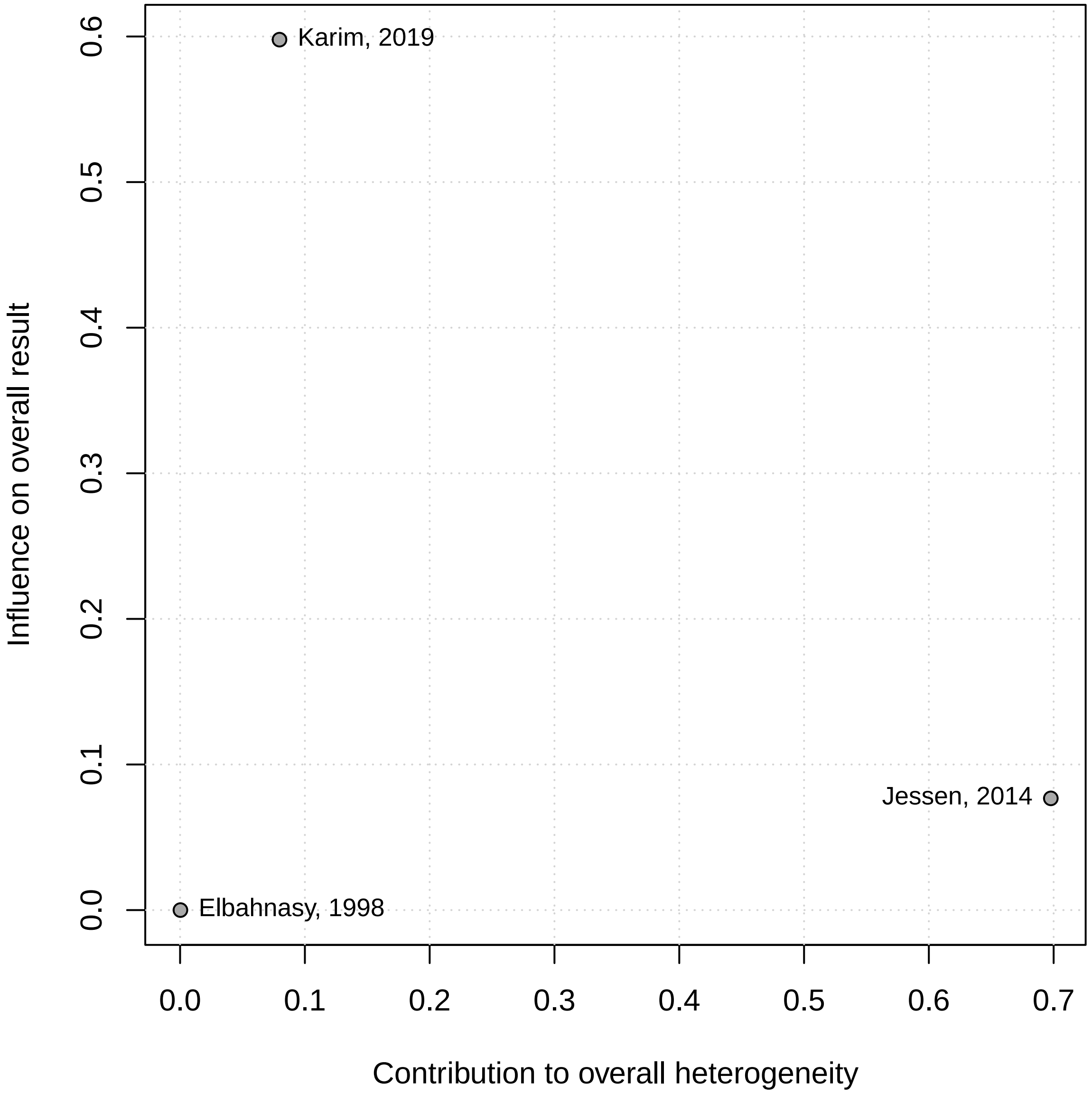

11 SF for LP stones >2cm

11.1 Sort Data

```
sf_data_more2cm <- sf_data %>%
  subset(stones_2mm == "yes") %>% as_tibble()



sf_data_more2cm_nsf <- sf_data_more2cm %>% filter(outcome !="SF") %>% mutate("mean_angle_nsf" = mean_an
                                                        "mean_angle_sd_nsf" = mean_angle_sd,
                                                        "n.e" = number_in_study)
sf_data_more2cm_sf <- sf_data_more2cm %>% filter(outcome !="NSF") %>% mutate("mean_angle_sf" = mean_ang
                                                        "mean_angle_sd_sf" = mean_angle_sd,
                                                        "n.c" = number_in_study)

sf_data_more2cm_reconfig <- cbind(sf_data_more2cm_nsf, sf_data_more2cm_sf)
sf_data_more2cm_reconfig$mean_angle_nsf<-as.numeric(sf_data_more2cm_reconfig$mean_angle_nsf)
sf_data_more2cm_reconfig$mean_angle_sd_nsf<-as.numeric(sf_data_more2cm_reconfig$mean_angle_sd_nsf)
sf_data_more2cm_reconfig$mean_angle_sf<-as.numeric(sf_data_more2cm_reconfig$mean_angle_sf)
sf_data_more2cm_reconfig$mean_angle_sd_sf<-as.numeric(sf_data_more2cm_reconfig$mean_angle_sd_sf)
sf_data_more2cm_reconfig$n.e<-as.numeric(sf_data_more2cm_reconfig$n.e)
sf_data_more2cm_reconfig$n.c<-as.numeric(sf_data_more2cm_reconfig$n.c)
```

11.1.1 Overall Number of patients

```r
sum(sf_data_more2cm$number_in_study)
```

```
## [1] 980
```

11.1.2 SF - Number of patients

```
sum(sf_data_more2cm_sf$number_in_study)
```

```
## [1] 697
```

11.1.3 NSF - Number of patients

```
sum(sf_data_more2cm_nsf$number_in_study)
```

```
## [1] 283
```

11.2 Summary forestplot for SF

Above 67o all patients are stone free Below 30o no patients are stone free

These cut-offs fit with Kilicarslan (>70) and Geavlete (<30) respectively (see below)

By excluding Sari et al., we move the angle at which all patients become SF to ~60 Need to check how SF defined - is definition accounting for difference in SFR between Sari and remainder?

```
sf_data_more2cm <- sf_data_more2cm %>% mutate("lower" = mean_angle - mean_angle_sd,
                              "upper" = mean_angle + mean_angle_sd,
                              "angle" = mean_angle)

sf_data_more2cm$lower <- as.numeric(sf_data_more2cm$lower)
sf_data_more2cm$upper <- as.numeric(sf_data_more2cm$upper)
sf_data_more2cm$angle <- as.numeric(sf_data_more2cm$angle)
sf_data_more2cm$outcome <- as.factor(sf_data_more2cm$outcome)

sf_data_more2cm <- sf_data_more2cm %>% mutate(stud_lab = paste(author,
                                              year,
                                              sep = ", "))

sf_data_sf_more2cm <- metamean(n = number_in_study,
                   mean = mean_angle,
                   sd = mean_angle_sd,
                   studlab = author,
                   data = subset(sf_data_more2cm,
                                 outcome == "SF"),
                   sm = "MRAW",
                   fixed = FALSE,
                   random = TRUE,
                   method.tau = "REML",
                   hakn = TRUE,
                   title = "SF Mean")
sf_data_nsf_more2cm <-metamean(n = number_in_study,
                   mean = mean_angle,
                   sd = mean_angle_sd,
                   studlab = author,
                   data = subset(sf_data_more2cm,
                                 outcome == "NSF"),
                   sm = "MRAW",
                   fixed = FALSE,
                   random = TRUE,
                   method.tau = "REML",
                   hakn = TRUE,
                   title = "NSF Mean")

sf_data_more2cm_minimised <- sf_data_more2cm %>% subset(select = c(stud_lab,
                                                   outcome,
                                                   angle,
                                                   lower,
                                                   upper)) %>% rbind(c(
                                                     "Overall",
                                                     "SF",
                                                     round(sf_data_sf_more2cm$TE.random),
                                                     round(sf_data_sf_more2cm$lower.random),
```

```
                                                    round(sf_data_sf_more2cm$upper.random)
                                                  )) %>% rbind(c(
                                                    "Overall",
                                                    "NSF",
                                                    round(sf_data_nsf_more2cm$TE.random),
                                                    round(sf_data_nsf_more2cm$lower.random),
                                                    round(sf_data_nsf_more2cm$upper.random)
                                                  ))

sf_data_more2cm_minimised$outcome <- factor(sf_data_more2cm_minimised$outcome,
                                    levels = c("SF",
                                               "NSF"))
sf_data_more2cm_minimised$angle<- as.numeric(sf_data_more2cm_minimised$angle)
sf_data_more2cm_minimised$lower<- as.numeric(sf_data_more2cm_minimised$lower)
sf_data_more2cm_minimised$upper<- as.numeric(sf_data_more2cm_minimised$upper)

sf_data_more2cm_minimised %>% group_by(outcome) %>% forestplot(
  mean = angle,
  lower = lower,
  upper = upper,
  labeltext = stud_lab,
  fn.ci_norm = c(fpDrawNormalCI, fpDrawCircleCI),
  zero = 67,
  vertices = TRUE,
  cex = 2,
  lineheight = "auto",
  xlab = "Infundibulopelvic Angle (Degrees)",
  xticks = c(0, 10, 20, 30, 40, 50, 60, 70, 80, 90, 100),
) %>% fp_set_style(
  box = c("blue", "darkred"),
  line = "black",
  txt_gp = fpTxtGp(
    ticks = gpar(fontfamily = "", cex = 1),
    xlab  = gpar(fontfamily = "", cex = 1)
  )
) %>% fp_add_lines("steelblue") %>%
  fp_add_header("Outcome") %>% fp_set_zebra_style("#EFEFEFEF")
```

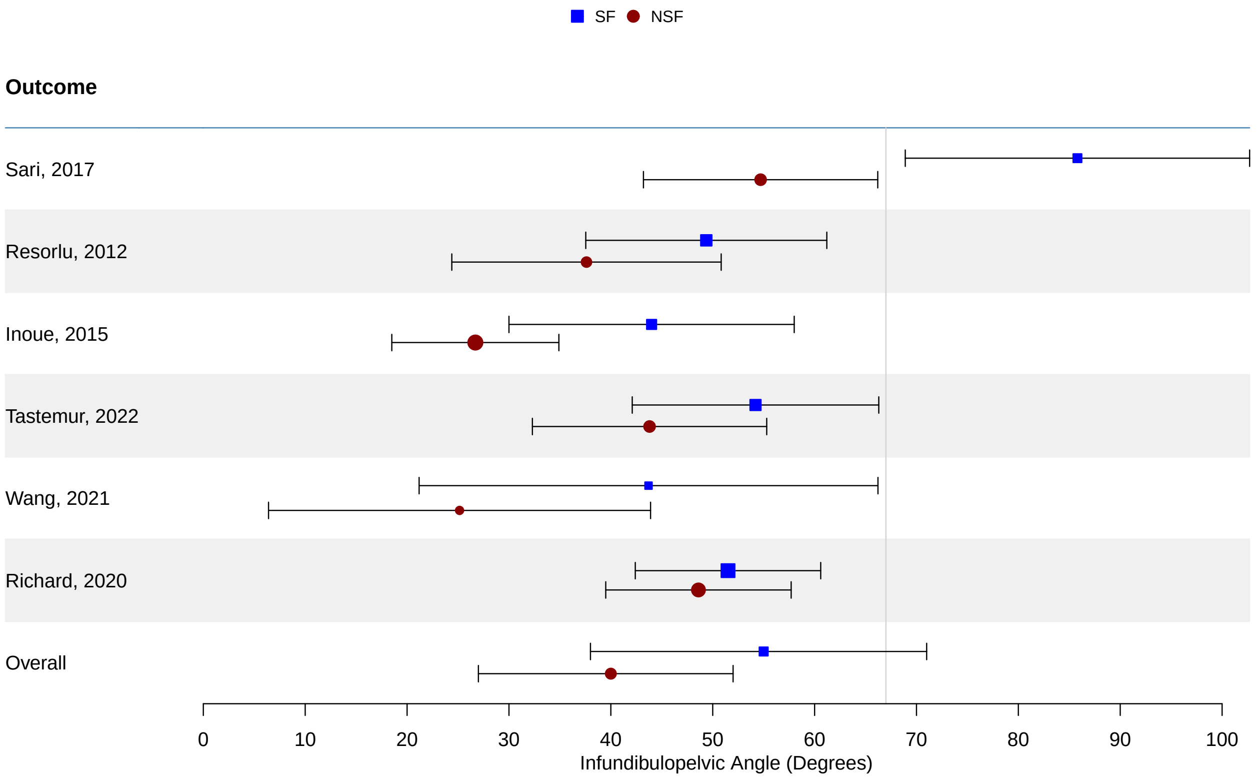
SF
NSF
Outcome
Sari, 2017
Resorlu, 2012
Inoue, 2015
Tastemur, 2022
Wang, 2021
Richard, 2020
Overall
0
10
20
30
40
50
60
70
80
90
100
Infundibulopelvic Angle (Degrees)

11.3 Meta-Analysis

11.3.1 Result

```
sf_more2cm_rma <- metacont(data = sf_data_more2cm_reconfig,
                   mean.e = mean_angle_nsf,
                   sd.e = mean_angle_sd_nsf,
                   n.e = n.e,
                   mean.c = mean_angle_sf,
                   sd.c = mean_angle_sd_sf,
                   n.c = n.c,
                   studlab = paste(author, year, sep = ", ")
                   )
sf_more2cm_rma
```

```
## Number of studies: k = 6
## Number of observations: o = 980
##
##                              MD               95%-CI      z  p-value
## Common effect model   -7.9059 [ -9.4045; -6.4072] -10.34 < 0.0001
## Random effects model -15.1382 [-23.0976; -7.1787]  -3.73   0.0002
##
## Quantifying heterogeneity:
##  tau^2 = 89.6278 [30.2744; 541.4006]; tau = 9.4672 [5.5022; 23.2680]
##  I^2 = 95.7% [92.9%; 97.4%]; H = 4.84 [3.76; 6.22]
##
## Test of heterogeneity:
##       Q d.f.  p-value
##  117.11    5 < 0.0001
##
## Details on meta-analytical method:
## - Inverse variance method
## - Restricted maximum-likelihood estimator for tau^2
## - Q-Profile method for confidence interval of tau^2 and tau
```

11.3.2 Forest plot

```r
forest(sf_more2cm_rma,
       sortvar = TE)
```

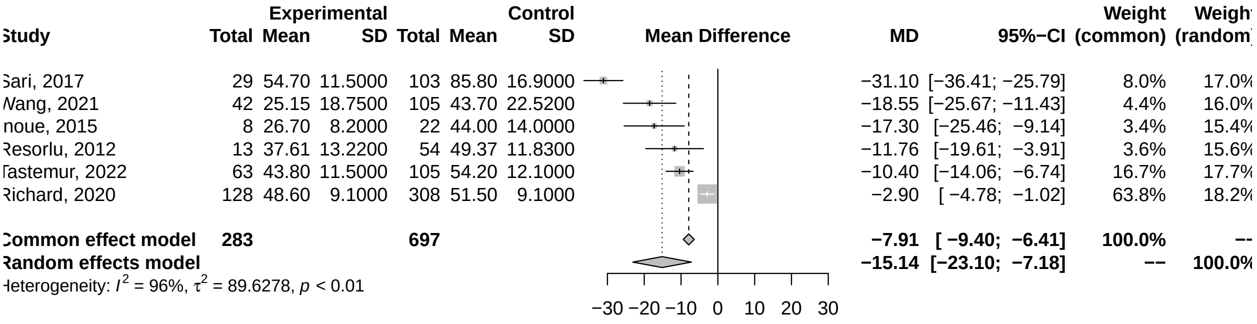

11.3.3 Trim and Fill

```r
trimfill(sf_more2cm_rma)
```

```
## Number of studies: k = 9 (with 3 added studies)
## Number of observations: o = 1289
##
##                              MD              95%-CI     z p-value
## Random effects model -5.9607 [-16.7017; 4.7804] -1.09  0.2767
##
## Quantifying heterogeneity:
##  tau^2 = 259.7384 [112.8579; 976.7624]; tau = 16.1164 [10.6235; 31.2532]
##  I^2 = 96.8% [95.4%; 97.8%]; H = 5.61 [4.68; 6.73]
##
## Test of heterogeneity:
##       Q d.f.  p-value
##  251.84    8 < 0.0001
##
## Details on meta-analytical method:
## - Inverse variance method
## - Restricted maximum-likelihood estimator for tau^2
## - Q-Profile method for confidence interval of tau^2 and tau
## - Trim-and-fill method to adjust for funnel plot asymmetry (L-estimator)
```

```r
funnel(trimfill(sf_more2cm_rma))
```

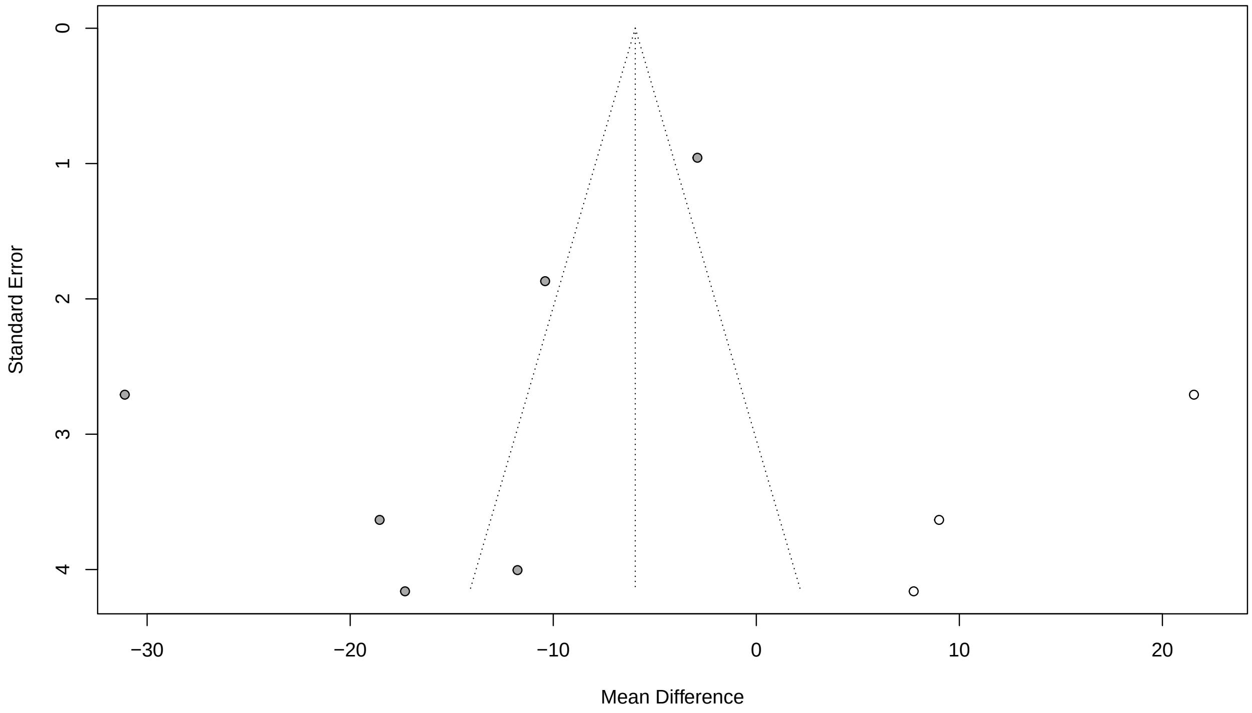


```r
forest(trimfill(sf_more2cm_rma),
       sortvar = TE)
```

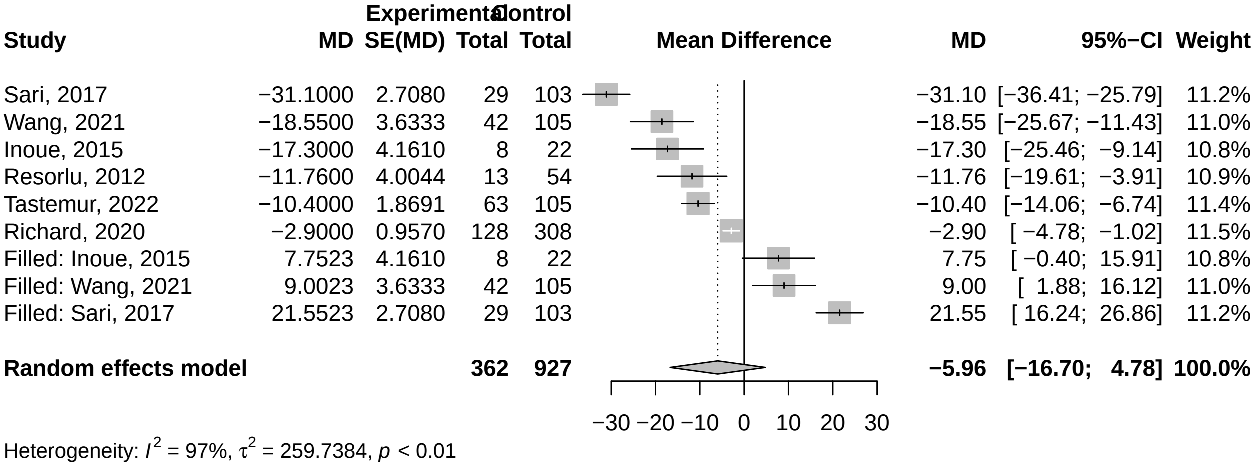

11.3.4 Baujat

```
baujat(sf_more2cm_rma)
```

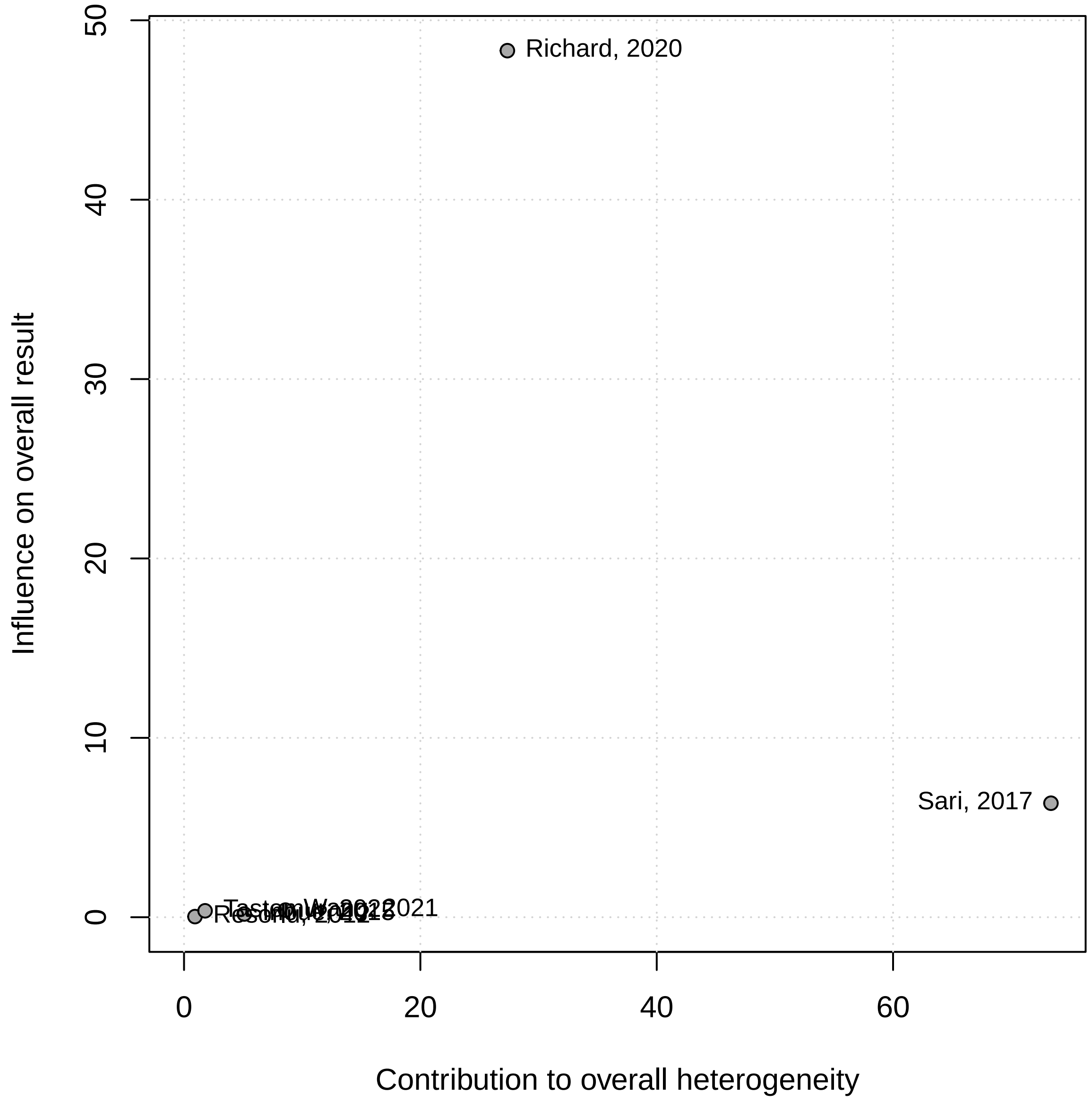
Richard, 2020
Sari, 2017
Influence on overall result
Contribution to overall heterogeneity
0
10
20
30
40
50
0
20
40
60

12 Summary Forest plot for Overall Findings

```r
sf_data_minimised_overall <- rbind(
  subset(sf_data_minimised,
         stud_lab == "Overall"),
  subset(sf_data_minimised_exc_Sari,
         stud_lab == "Overall"),
  subset(sf_data_x2_minimised,
         stud_lab == "Overall"),
  subset(sf_data_non_x2_minimised,
         stud_lab == "Overall"),
  subset(sf_data_no_frags_minimised,
         stud_lab == "Overall"),
  subset(sf_data_frags_minimised,
         stud_lab == "Overall"),
  subset(sf_data_2cm_minimised,
         stud_lab == "Overall"),
  subset(sf_data_more2cm_minimised,
         stud_lab == "Overall")
) %>% cbind(
  "analysis" = c(
    "All",
    "All",
    "All excluding Sari",
    "All excluding Sari",
    "Flex X2",
    "Flex X2",
    "Non Flex X2",
    "Non Flex X2",
    "No fragments",
    "No fragments",
    "Fragments <4mm",
    "Fragments <4mm",
    "Studies with Only Stones <20mm",
    "Studies with Only Stones <20mm",
    "Studies Including Stones >20mm",
    "Studies Including Stones >20mm"
  )
) %>% cbind("n_studies" = c((nrow(sf_data_minimised)/2)-1,
                            (nrow(sf_data_minimised)/2)-1,
                            (nrow(sf_data_minimised_exc_Sari)/2)-1,
                            (nrow(sf_data_minimised_exc_Sari)/2)-1,
                            (nrow(sf_data_x2_minimised)/2)-1,
                            (nrow(sf_data_x2_minimised)/2)-1,
                            (nrow(sf_data_non_x2_minimised)/2)-1,
                            (nrow(sf_data_non_x2_minimised)/2)-1,
                            (nrow(sf_data_no_frags_minimised)/2)-1,
                            (nrow(sf_data_no_frags_minimised)/2)-1,
                            (nrow(sf_data_frags_minimised)/2)-1,
                            (nrow(sf_data_frags_minimised)/2)-1,
                            (nrow(sf_data_2cm_minimised)/2)-1,
                            (nrow(sf_data_2cm_minimised)/2)-1,
                            (nrow(sf_data_more2cm_minimised)/2)-1,
```

```
                            (nrow(sf_data_more2cm_minimised)/2)-1
                            ))

sf_data_minimised_overall$outcome <- factor(sf_data_minimised_overall$outcome,
                                    levels = c("SF",
                                               "NSF"))
sf_data_minimised_overall$angle<- as.numeric(sf_data_minimised_overall$angle)
sf_data_minimised_overall$lower<- as.numeric(sf_data_minimised_overall$lower)
sf_data_minimised_overall$upper<- as.numeric(sf_data_minimised_overall$upper)

sf_data_minimised_overall %>% group_by(outcome) %>% forestplot(
  mean = angle,
  lower = lower,
  upper = upper,
  labeltext = c(analysis,
                n_studies),
  fn.ci_norm = c(fpDrawNormalCI, fpDrawCircleCI),
  vertices = TRUE,
  cex = 2,
  lineheight = "auto",
  xlab = "Infundibulopelvic Angle (Degrees)",
  xticks = c(0, 10, 20, 30, 40, 50, 60, 70, 80, 90),
) %>% fp_set_style(
  box = c("blue", "darkred"),
  line = "black",
  txt_gp = fpTxtGp(
    ticks = gpar(fontfamily = "", cex = 1),
    xlab  = gpar(fontfamily = "", cex = 1)
  )
) %>% fp_add_lines("steelblue") %>%
  fp_add_header("Analysis", "Studies, n") %>% fp_set_zebra_style("#EFEFEFEF") %>%
  fp_decorate_graph(grid = structure(c(35, 62),
                                     gp = gpar(lty = 2, col = "#CCCCFF")))
```

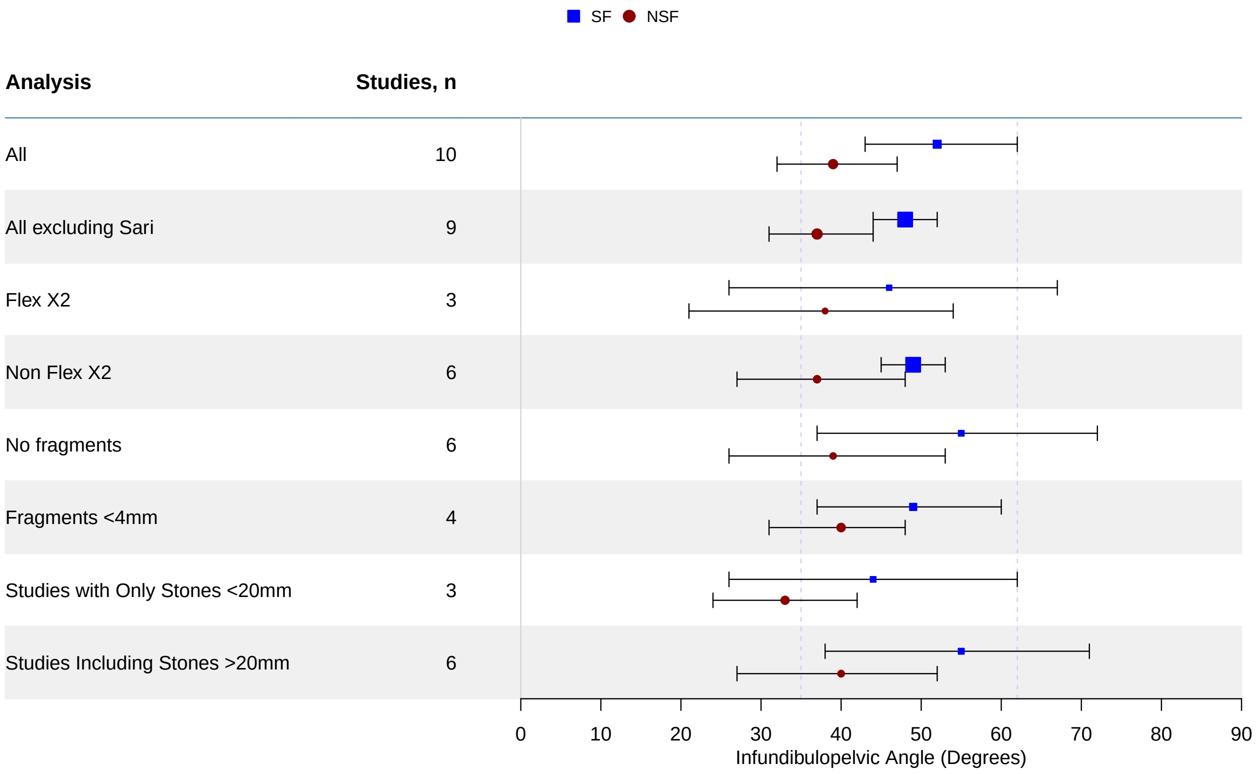

13 Angle cut-off

13.1 Sort data

```
angle_data1 <- lpa_data %>% filter(outcome != "SF",
                                   outcome != "NSF")
angle_data1$angle_definition <- as.factor(angle_data1$angle_definition)
```

13.2 Summary table

```
angle_data1 <- angle_data1 %>% mutate(
  sfr = (sf_n / number_in_study * 100))
  angle_data1$sfr <- round(as.numeric(angle_data1$sfr), digits = 0)

  angle_data1 %>% subset(select = c(author,
                                   year,
                                   angle_definition,
                                   number_in_study,
                                   stones_2mm,
                                   sfr)) %>% group_by(angle_definition) %>% gt() %>% gt_theme_espn(
                                   )
```

author	year	number_in_study	stones_2mm	sfr
<45				
Resorlu	2012	69	no	28
Richard	2020	114	yes	62
>45				
Resorlu	2012	19	no	184
Richard	2020	322	yes	74
<30				
Geavlete	2008	4	no	0
30-90				
Geavlete	2008	35	no	74
>90				
Geavlete	2008	8	no	88
< 70				
Kilicarslan	2015	19	yes	58
>70				
Kilicarslan	2015	17	yes	100

14 Operative Time

14.1 How many studies detail Operative time?

```
lpa_data %>% subset(!is.na(operative_time)) %>% subset(select = c(author,
                                                                   year,
                                                                   outcome,
                                                                   operative_time,
                                                                   operative_time_sd)) %>% gt()
```

author	year	outcome	operative_time	operative_time_sd
Sari	2017	SF	45.3	12.9
Sari	2017	NSF	47.4	12.3
Elbahnasy	1998	SF	93.4	43.3
Elbahnasy	1998	NSF	93.4	43.3
Tastemur	2022	SF	48.1	14.8
Tastemur	2022	NSF	58.7	18.2
Karim	2019	SF	47.9	26.7
Karim	2019	NSF	74.7	35.7
Xiao	2017	SF	50.0	20.0
Xiao	2017	NSF	60.0	35.0
Kilicarslan	2015	Steep	72.6	49.2
Kilicarslan	2015	Shallow	48.2	26.5

14.2 Analyse Operative time

```
op_time_data <- lpa_data %>% subset(!is.na(operative_time))
op_time_data <- op_time_data %>% subset(author != "Kilicarslan")

op_time_data_nsf <- op_time_data %>% filter(outcome !="SF") %>% mutate("mean_operative_time_nsf" = opera
                                                             "mean_operative_time_nsf_sd" = operative_time_sd
                                                             "n.e" = number_in_study)
op_time_data_sf <- op_time_data %>% filter(outcome !="NSF") %>% mutate("mean_operative_time_sf" = operat
                                                             "mean_operative_time_sf_sd" = operative_time_sd,
                                                             "n.c" = number_in_study)

op_time_data_reconfig <- cbind(op_time_data_nsf, op_time_data_sf)
op_time_data_reconfig$mean_operative_time_nsf<-as.numeric(op_time_data_reconfig$mean_operative_time_nsf)
op_time_data_reconfig$mean_operative_time_nsf_sd<-as.numeric(op_time_data_reconfig$mean_operative_time_n
op_time_data_reconfig$mean_operative_time_sf<-as.numeric(op_time_data_reconfig$mean_operative_time_sf)
op_time_data_reconfig$mean_operative_time_sf_sd<-as.numeric(op_time_data_reconfig$mean_operative_time_sf
op_time_data_reconfig$n.e<-as.numeric(op_time_data_reconfig$n.e)
op_time_data_reconfig$n.c<-as.numeric(op_time_data_reconfig$n.c)
```

14.2.1 Overall Number of patients

```r
sum(op_time_data$number_in_study)
```

```
## [1] 566
```

14.2.2 SF - Number of patients

```
sum(op_time_data_sf$number_in_study)
```

```
## [1] 387
```

14.2.3 NSF - Number of patients

```r
sum(op_time_data_nsf$number_in_study)
```

```
## [1] 179
```

14.3 Summary forestplot for SF

Above 67o all patients are stone free Below 30o no patients are stone free

These cut-offs fit with Kilicarslan (>70) and Geavlete (<30) respectively (see below)

By excluding Sari et al., we move the angle at which all patients become SF to ~60 Need to check how SF defined - is definition accounting for difference in SFR between Sari and remainder?

```
op_time_data <- op_time_data %>% mutate("lower" = operative_time - operative_time_sd,
                              "upper" = operative_time + operative_time_sd,
                              "operative_time" = operative_time)

op_time_data$lower <- as.numeric(op_time_data$lower)
op_time_data$upper <- as.numeric(op_time_data$upper)
op_time_data$operative_time <- as.numeric(op_time_data$operative_time)
op_time_data$outcome <- as.factor(op_time_data$outcome)

op_time_data <- op_time_data %>% mutate(stud_lab = paste(author,
                                              year,
                                              sep = ", "))

op_time_data_sf_1 <- metamean(n = number_in_study,
                   mean = operative_time,
                   sd = operative_time_sd,
                   studlab = author,
                   data = subset(op_time_data,
                                 outcome == "SF"),
                   sm = "MRAW",
                   fixed = FALSE,
                   random = TRUE,
                   method.tau = "REML",
                   hakn = TRUE,
                   title = "SF Mean")
op_time_data_nsf_1 <-metamean(n = number_in_study,
                   mean = operative_time,
                   sd = operative_time_sd,
                   studlab = author,
                   data = subset(op_time_data,
                                 outcome == "NSF"),
                   sm = "MRAW",
                   fixed = FALSE,
                   random = TRUE,
                   method.tau = "REML",
                   hakn = TRUE,
                   title = "NSF Mean")

op_time_data_minimised <- op_time_data %>% subset(select = c(stud_lab,
                                                    outcome,
                                                    operative_time,
                                                    lower,
                                                    upper)) %>% rbind(c(
                                                      "Overall",
                                                      "SF",
                                                      round(op_time_data_sf_1$TE.random),
                                                      round(op_time_data_sf_1$lower.random),
```

```
                                                     round(op_time_data_sf_1$upper.random)
                                                   )) %>% rbind(c(
                                                     "Overall",
                                                     "NSF",
                                                     round(op_time_data_nsf_1$TE.random),
                                                     round(op_time_data_nsf_1$lower.random),
                                                     round(op_time_data_nsf_1$upper.random)
                                                   ))

op_time_data_minimised$outcome <- factor(op_time_data_minimised$outcome,
                                    levels = c("SF",
                                               "NSF"))
op_time_data_minimised$operative_time<- as.numeric(op_time_data_minimised$operative_time)
op_time_data_minimised$lower<- as.numeric(op_time_data_minimised$lower)
op_time_data_minimised$upper<- as.numeric(op_time_data_minimised$upper)

op_time_data_minimised %>% group_by(outcome) %>% forestplot(
  mean = operative_time,
  lower = lower,
  upper = upper,
  labeltext = stud_lab,
  fn.ci_norm = c(fpDrawNormalCI, fpDrawCircleCI),
  zero = 67,
  vertices = TRUE,
  cex = 2,
  lineheight = "auto",
  xlab = "Operative Time (Minutes)",
  xticks = c(0, 10, 20, 30, 40, 50, 60, 70, 80, 90, 100),
) %>% fp_set_style(
  box = c("blue", "darkred"),
  line = "black",
  txt_gp = fpTxtGp(
    ticks = gpar(fontfamily = "", cex = 1),
    xlab  = gpar(fontfamily = "", cex = 1)
  )
) %>% fp_add_lines("steelblue") %>%
  fp_add_header("Outcome") %>% fp_set_zebra_style("#EFEFEFEF")
```

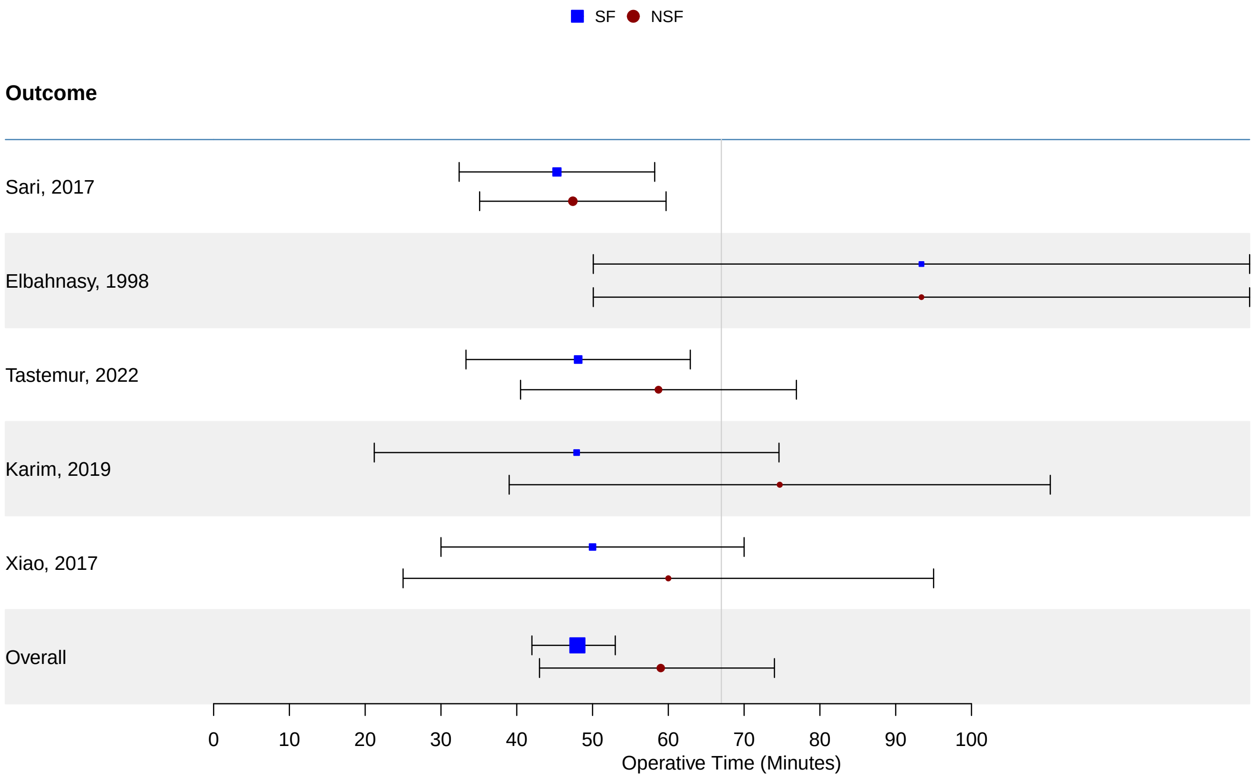
SF
NSF
Outcome
Sari, 2017
Elbahnasy, 1998
Tastemur, 2022
Karim, 2019
Xiao, 2017
Overall
0
10
20
30
40
50
60
70
80
90
100
Operative Time (Minutes)

14.4 Meta-Analysis

14.4.1 Result

```
op_time_rma <- metacont(data = op_time_data_reconfig,
                   mean.e = mean_operative_time_nsf,
                   sd.e = mean_operative_time_nsf_sd,
                   n.e = n.e,
                   mean.c = mean_operative_time_sf,
                   sd.c = mean_operative_time_sf_sd,
                   n.c = n.c,
                   studlab = paste(author, year, sep = ", ")
                   )
op_time_rma
```

```
## Number of studies: k = 5
## Number of observations: o = 566
##
##                            MD            95%-CI    z  p-value
## Common effect model  6.9662 [3.5766; 10.3557] 4.03 < 0.0001
## Random effects model 7.8197 [1.9713; 13.6682] 2.62   0.0088
##
## Quantifying heterogeneity:
##  tau^2 = 17.5573 [0.0000; >175.5729]; tau = 4.1901 [0.0000; >13.2504]
##  I^2 = 47.1% [0.0%; 80.6%]; H = 1.37 [1.00; 2.27]
##
## Test of heterogeneity:
##     Q d.f. p-value
##  7.56    4  0.1093
##
## Details on meta-analytical method:
## - Inverse variance method
## - Restricted maximum-likelihood estimator for tau^2
## - Q-Profile method for confidence interval of tau^2 and tau
```

14.4.2 Forest plot

```r
forest(op_time_rma,
       sortvar = TE)
```

Study	Experimental Total	Experimental Mean	Experimental SD	Control Total	Control Mean	Control SD	MD	95%−CI	Weight (common)	Weight (random)
Elbahnasy, 1998	5	93.40	43.3000	8	93.40	43.3000	0.00	[−48.38; 48.38]	0.5%	1.4%
Sari, 2017	29	47.40	12.3000	103	45.30	12.9000	2.10	[−3.02; 7.22]	43.8%	36.5%
Xiao, 2017	76	60.00	35.0000	69	50.00	20.0000	10.00	[0.82; 19.18]	13.6%	22.6%
Tastemur, 2022	63	58.70	18.2000	105	48.10	14.8000	10.60	[5.29; 15.91]	40.7%	35.8%
Karim, 2019	6	74.70	35.7000	102	47.90	26.7000	26.80	[−2.23; 55.83]	1.4%	3.8%
**Common effect model**	**179**			**387**			**6.97**	**[3.58; 10.36]**	**100.0%**	**−−**
**Random effects model**							**7.82**	**[1.97; 13.67]**	**−−**	**100.0%**

Heterogeneity: $I^2 = 47\%$, $\tau^2 = 17.5573$, $p = 0.11$

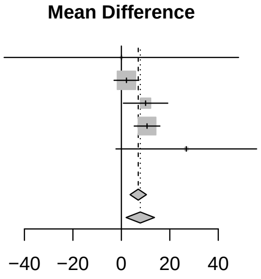

14.4.3 Trim and Fill

```r
trimfill(op_time_rma)
```

```
## Number of studies: k = 5 (with 0 added studies)
## Number of observations: o = 566
##
##                           MD            95%-CI    z p-value
## Random effects model 7.8197 [1.9713; 13.6682] 2.62  0.0088
##
## Quantifying heterogeneity:
##  tau^2 = 17.5573 [0.0000; >175.5729]; tau = 4.1901 [0.0000; >13.2504]
##  I^2 = 47.1% [0.0%; 80.6%]; H = 1.37 [1.00; 2.27]
##
## Test of heterogeneity:
##     Q d.f. p-value
##  7.56    4  0.1093
##
## Details on meta-analytical method:
## - Inverse variance method
## - Restricted maximum-likelihood estimator for tau^2
## - Q-Profile method for confidence interval of tau^2 and tau
## - Trim-and-fill method to adjust for funnel plot asymmetry (L-estimator)
```

```r
funnel(trimfill(op_time_rma))
```

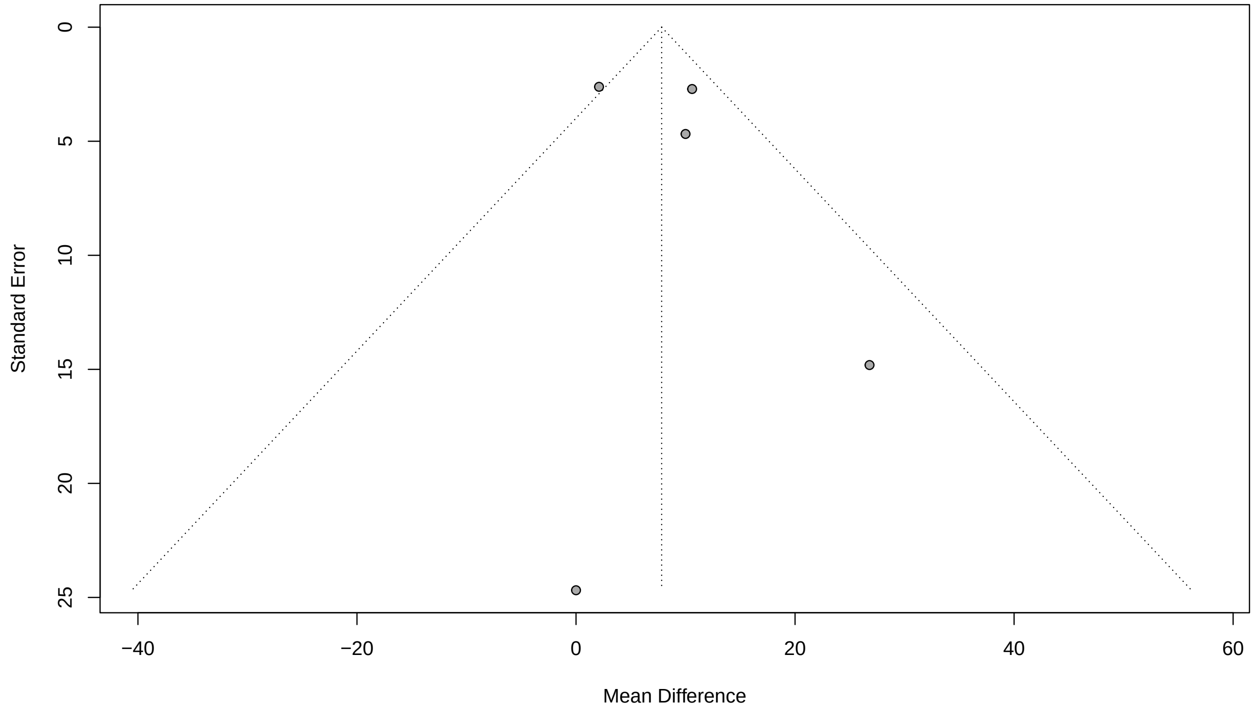


```r
forest(trimfill(op_time_rma),
       sortvar = TE)
```

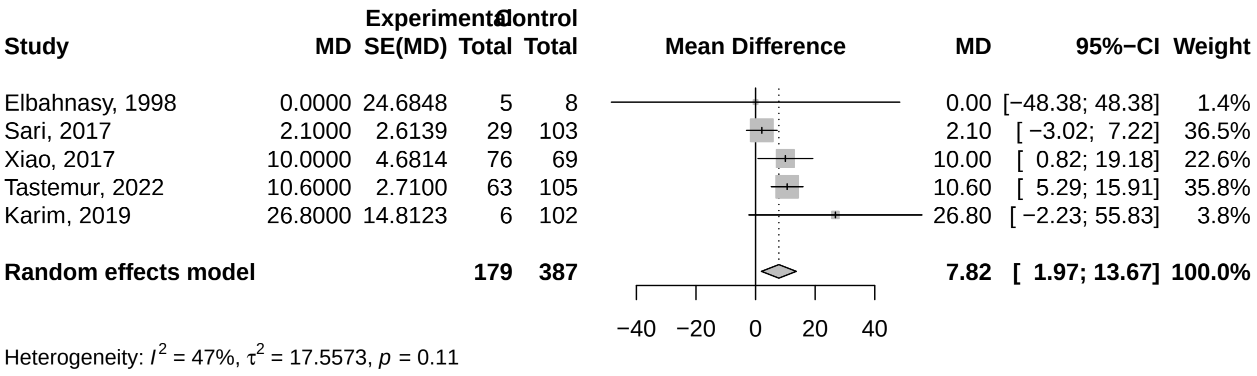

14.4.4 Baujat

```r
baujat(op_time_rma)
```

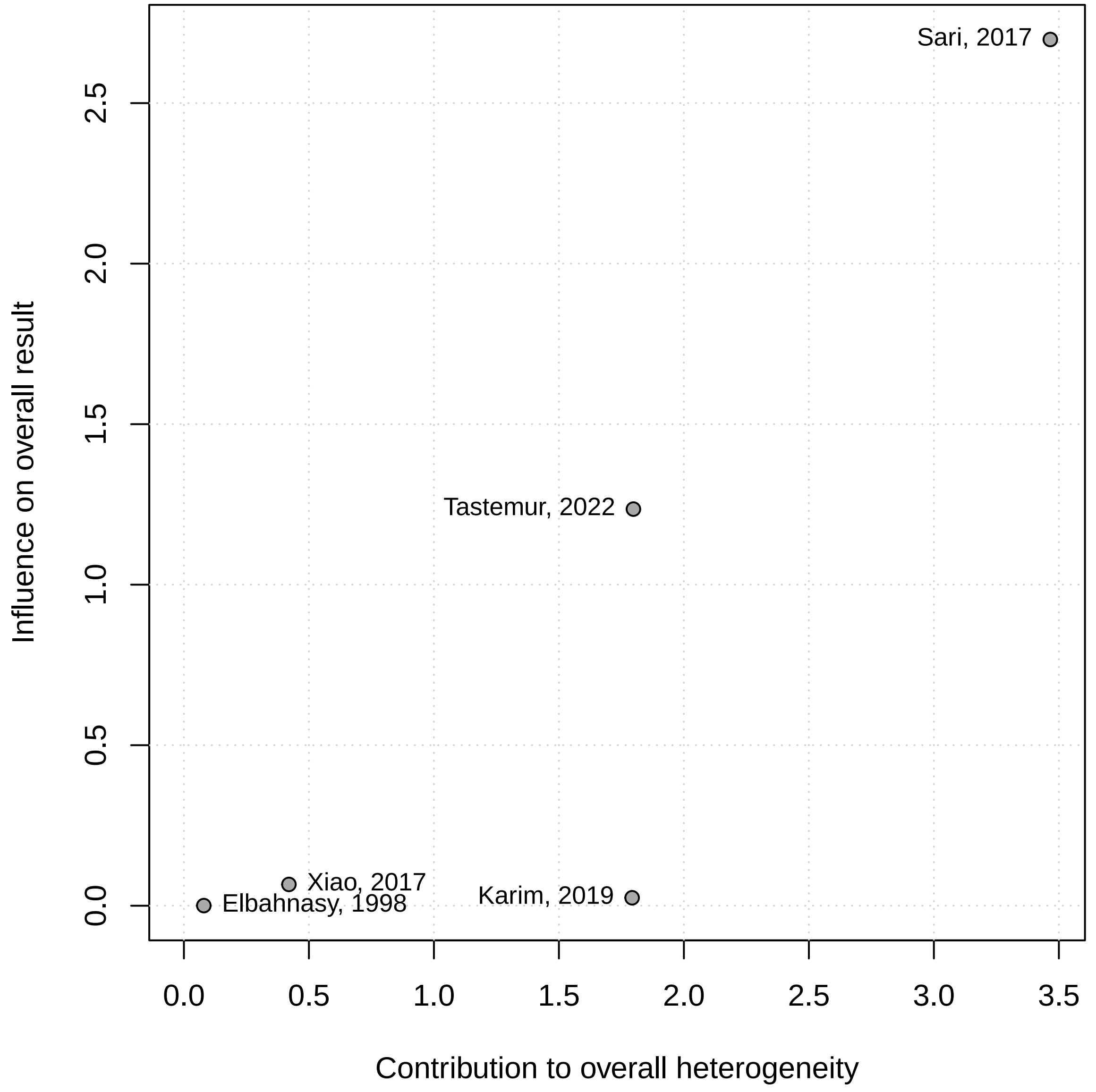

Supplement: Supplementary file 1 — Supplementary Material 1 [file 345_2024_5104_MOESM1_ESM.pdf]
