# Supplementary material for: Effect of infundibulopelvic angle on outcomes of ureteroscopy: a systematic review and meta-analysis
Source: World J Urol. 2024 Jul 16;42(1):413. doi: 10.1007/s00345-024-05104-z (PMC11252207; doi:10.1007/s00345-024-05104-z)
Supplement: Supplementary file 2 — Supplementary Material 2 [file 345_2024_5104_MOESM2_ESM.docx]

**

*Supplementary Figure 1. PRISMA flow diagram for study selection.*

|  | **Complications, n** | | | | |
| --- | --- | --- | --- | --- | --- |
| **Author** | **Overall, n (%)** | **CD I** | **CD II** | **CD III** | **CD IV/V** |
| Sari et al. 2017^11^ | NA | NA | NA | NA | NA |
| Resorlu et al. 2012 (a)^12^ | 20 (23%) | 4 (2%) | 13 (15%) | 2 (1%) | 1 (0.5%) |
| Elbahnasy et al. 1998^22^ | NA | NA | NA | NA | NA |
| Resorlu et al. 2012 (b)^13^ | NA | NA | NA | NA | NA |
| Jessen et al. 2014^14^ | 22 (20%) | 4 (4%) | 11 (10%) | 6 (6%) | 1 (1%) |
| Geavlete et al. 2008^19^ | NA | NA | NA | NA | NA |
| Inoue et al. 2005^15^ | 9 (13%) | 2 (3%) | 6 (9%) | 1 (2%) | 0 |
| Tastemur et al. 2022^16^ | NA | NA | NA | NA | NA |
| Wang et al. 2021^17^ | NA | NA | NA | NA | NA |
| Richard et al. 2020^18^ | 69 (10%) | 32 (5%) | 33 (5%) | 3 (0.5%) | 1 (0.2%) |
| Karim et al.2019^2^ | 2 (2%) | 1 (1%) | 0 | 1 (1%) | 0 |
| Kilicarslan et al. 2015^20^ | NA | NA | NA | NA | NA |
| Xiao et al. 2017^21^ | 13 (9%) | NA | NA | NA | NA |

*Supplementary Table 1. Complication rates for each study.*

| **Study Defined Angle Cut-off** | **Study** | **Patients, n** | **Stones >2cm included** | **SFR (%)** |
| --- | --- | --- | --- | --- |
| **<30** | Geavlete et al. 2008 | 4 | no | 0 |
| **<45** | Resorlu et al. 2012 (a) | 40 | no | 65 |
|  | Richard et al. 2020 | 114 | yes | 62 |
| **>45** | Resorlu et al. 2012 (a) | 46 | no | 91 |
|  | Richard et al. 2020 | 322 | yes | 74 |
| **30-90** | Geavlete et al. 2008 | 35 | no | 74 |
| **< 70** | Kilicarslan et al. 2015 | 19 | yes | 58 |
| **>70** | Kilicarslan et al. 2015 | 17 | yes | 100 |
| **>90** | Geavlete et al. 2008 | 8 | no | 88 |

*Supplementary Table 2. Details of the studies that examined stone free rates dependent on IPA.*

| **Author** | **Selection (/4)** | | | | **Comparability (/2)** | **Outcome (/3)** | | | **Overall (/9)** |
| --- | --- | --- | --- | --- | --- | --- | --- | --- | --- |
|  | **Representativeness of exposed cohort** | **Selection of non exposed cohort** | **Ascertainment of exposure** | **Demonstration that outcome of interest was not present at start of study** | **Comparability of cohorts** | **Assessment of outcome** | **Length of follow-up** | **Adequacy of follow-up cohorts** |  |
| Sari et al. 2017^11^ |  | ♦ | ♦ | ♦ | ♦ |  |  | ♦ | **5** |
| Resorlu et al. 2012 (a)^12^ |  | ♦ | ♦ | ♦ | ♦ |  | ♦ | ♦ | **6** |
| Elbahnasy et al. 1998^22^ | ♦ | ♦ | ♦ | ♦ | ♦ |  | ♦ | ♦ | **7** |
| Resorlu et al. 2012 (b)^13^ |  | ♦ | ♦ | ♦ | ♦♦ |  | ♦ | ♦ | **7** |
| Jessen et al. 2014^14^ | ♦ | ♦ | ♦ | ♦ | ♦ |  |  | ♦ | **6** |
| Geavlete et al. 2008^19^ |  | ♦ | ♦ | ♦ | ♦ |  |  | ♦ | **5** |
| Inoue et al. 2005^15^ |  | ♦ | ♦ | ♦ | ♦ |  | ♦ | ♦ | **6** |
| Tastemur et al. 2022^16^ |  | ♦ | ♦ | ♦ | ♦ |  | ♦ | ♦ | **6** |
| Wang et al. 2021^17^ |  | ♦ | ♦ | ♦ | ♦♦ | ♦ | ♦ | ♦ | **8** |
| Richard et al. 2020^18^ |  | ♦ | ♦ | ♦ | ♦ | ♦ | ♦ | ♦ | **7** |
| Karim et al.2019^2^ | ♦ | ♦ | ♦ | ♦ | ♦♦ | ♦ | ♦ | ♦ | **9** |
| Kilicarslan et al. 2015^20^ |  | ♦ | ♦ | ♦ | ♦ | ♦ |  | ♦ | **6** |
| Xiao et al. 2017^21^ |  | ♦ | ♦ | ♦ | ♦♦ |  | ♦ | ♦ | **7** |

*Supplementary Table 3. Bias Analysis*

| **Certainty assessment** | | | | | | | **№ of patients** | | **Effect** | | **Certainty** |
| --- | --- | --- | --- | --- | --- | --- | --- | --- | --- | --- | --- |
| **№ of studies** | **Study design** | **Risk of bias** | **Inconsistency** | **Indirectness** | **Imprecision** | **Other considerations** | **SF** | **NSF** | **Relative (95% CI)** | **Absolute (95% CI)** |  |
| 10 | non-randomised studies | not serious | serious^a^ | not serious | serious^a^ | publication bias strongly suspected strong association dose response gradient | 974 | 383 | - | MD **13 degrees more** (7.2 more to 18.7 more) | ⨁◯◯◯ Very low |
| 5 | non-randomised studies | not serious | not serious | not serious | not serious | none | 387 | 179 | - | MD **7.8 minutes more** (2 more to 13.7 more) | ⨁⨁◯◯ Low |

*Supplementary Table 4. GRADE Analysis,* ***CI:*** *confidence interval;* ***MD****: mean difference,* ***a****. High statistical heterogeneity and evidence of publication bias*
